# Secondary siRNAs in Plants: Biosynthesis, Various Functions, and Applications in Virology

**DOI:** 10.3389/fpls.2021.610283

**Published:** 2021-03-02

**Authors:** Neeti Sanan-Mishra, A. Abdul Kader Jailani, Bikash Mandal, Sunil K. Mukherjee

**Affiliations:** ^1^Plant RNAi Biology Group, International Centre for Genetic Engineering and Biotechnology, New Delhi, India; ^2^Advanced Center for Plant Virology, Division of Plant Pathology, Indian Agricultural Research Institute, New Delhi, India

**Keywords:** RNA silencing, synthetic tasiRNA induced gene silencing (SIGS), heterochromatic siRNA, VsiRNA, artificial tasiRNA constructs, virus resistance, microRNA induced gene silencing vector (MIGS)

## Abstract

The major components of RNA silencing include both transitive and systemic small RNAs, which are technically called secondary sRNAs. Double-stranded RNAs trigger systemic silencing pathways to negatively regulate gene expression. The secondary siRNAs generated as a result of transitive silencing also play a substantial role in gene silencing especially in antiviral defense. In this review, we first describe the discovery and pathways of transitivity with emphasis on RNA-dependent RNA polymerases followed by description on the short range and systemic spread of silencing. We also provide an in-depth view on the various size classes of secondary siRNAs and their different roles in RNA silencing including their categorization based on their biogenesis. The other regulatory roles of secondary siRNAs in transgene silencing, virus-induced gene silencing, transitivity, and *trans*-species transfer have also been detailed. The possible implications and applications of systemic silencing and the different gene silencing tools developed are also described. The details on mobility and roles of secondary siRNAs derived from viral genome in plant defense against the respective viruses are presented. This entails the description of other compatible plant–virus interactions and the corresponding small RNAs that determine recovery from disease symptoms, exclusion of viruses from shoot meristems, and natural resistance. The last section presents an overview on the usefulness of RNA silencing for management of viral infections in crop plants.

## Introduction

RNA silencing is a sequence-specific RNA degradation and inactivation mechanism, operative in most eukaryotes. It has also been implicated in the epigenetic events resulting in suppression of repetitive sequences including transposable elements (TEs) and imprinted genes. RNA silencing is now used as an umbrella term to encompass suppression of gene expression by all kinds of 21 to 24-nucleotide (nt) small RNAs (sRNAs), generated primarily due to the activity of enzymes like Dicers or Dicer-like proteins (DCLs). The sRNAs can be broadly categorized as small interfering RNAs (siRNAs) and microRNAs (miRNAs) in plant systems. This is grouped based on the mechanism of their biogenesis from precursor double-stranded RNAs (dsRNAs) or hairpin RNAs, respectively. The functional sRNAs are incorporated in the argonaute (AGO) protein of the RNA-induced silencing complex (RISC) that can act as a site-specific endonuclease on the cytoplasmic transcripts to enable posttranscriptional gene silencing (PTGS). The sRNAs can also target the genomic DNA in the nucleus to facilitate transcriptional gene silencing (TGS).

RNA silencing can move or spread from the point of initiation in a process technically known as transitive and systemic silencing (SS). The spreading component involves intermediary steps of siRNA primed amplification or expansion of the silencing signals mediated by RNA-dependent RNA polymerases (RDRs or RdRPs). The spread of silencing was initially discovered by Palaqui and colleagues in transgenic tobacco bearing the nitrate reductase (*Nia*) transgene. This caused cosuppression of endo-*Nia* gene, resulting in lack of nitrogen availability and thus chlorosis (Palauqui et al., 1996). Chlorosis was observed almost randomly in a few areas of leaves, which expanded to form large clusters. Chlorosis also transmitted to newly emerging leaves, establishing the phenomenon of SS. This was observed later in many cosuppressed plants. Breakthrough observation in SS came from grafting experiments, which demonstrated 100% transmission of *Nia*-silencing from the silenced rootstock to non-silenced transgenic scions. Since then, similar spread of silencing has been documented, in many plants including *Arabidopsis*, cucurbits, sunflowers, *Medicago*, and ferns (Voinnet, 2005). The role of RDRs in amplification of silencing signals and other aspects of transitive silencing will be elaborated in subsequent sections.

*Arabidopsis* represents one of the best-studied plant species for sRNA biogenesis and function. It possesses four DCLs, each associated with a specific function. DCL1 is involved in production of miRNAs, which play a major role in regulating processes related to growth, development, and stress response (Kurihara and Watanabe, 2004). The miRNAs are considered to be mobile and can act as local and distal signals, although direct evidence for this is too little (Pant et al., 2008; Carlsbecker et al., 2010; Skopelitis et al., 2017).

The DCL2, DCL3, and DCL4 are involved in production of 22-, 24-, and 21-nt siRNAs, respectively (Henderson et al., 2006; Mukherjee et al., 2012). The siRNAs can target endogenous sequences as well as exogenous sequences such as viruses and transgenes serving as the first line of host defense (Agrawal et al., 2003). DCL2 is responsible for synthesis of 22-nt siRNAs that contribute to the secondary siRNA biogenesis, antiviral defense, and plant development (Bouché et al., 2006; Chen D. et al., 2010; Garcia-Ruiz et al., 2010; Qin et al., 2017). The DCL4 enzyme processes the formation of 21-nt siRNAs from dsRNA to initiate primary silencing in plant antiviral defense (Xie et al., 2005; Qu et al., 2008; Chen H. M. et al., 2010; Wang et al., 2011) and protect plants from invasion of transgenes. The intricate functions of DCL2 and DCL4 are partially redundant in plant antiviral defense, and they are also involved in processing secondary transitive siRNAs and *trans*-acting siRNAs (tasiRNAs), as discussed below.

The DCL3 processes 24-nt siRNAs from transcripts that are initially transcribed from heterochromatic loci by RNA polymerase IV (Pol-IV) and then converted to dsRNA by RDR2 (Daxinger et al., 2008; Mosher et al., 2008; Matzke and Mosher, 2014). The 24-nt siRNAs are often associated with AGO4-containing RISC and less often with AGO6- and AGO9-containing RISC to direct methylation of DNA sequences resulting in chromatin modification and TGS (Zilberman et al., 2003; Henderson et al., 2006; Qi et al., 2006; Zheng et al., 2007; Wierzbicki et al., 2009; Havecker et al., 2010; Olmedo-Monfil et al., 2010; Melnyk et al., 2011a; Sarkies and Miska, 2014; Lewsey et al., 2016). TGS of repetitive DNA sequences including the TEs also occurs in a similar process requiring RNA Pol-V.

## Transitivity of sRNAs

One of the amazing characteristics of the silencing process is the requirement of catalytic amount of the trigger molecules that can cause silencing of numerous homologous transcripts (Hunter et al., 2006; Hinas et al., 2012). The silencing capability remains undiluted over cell divisions and can even silence the homologous genes in the untreated cells of whole organism, as evidenced in plants, nematodes, and other organisms. This indicates the presence of phenomenon-mediating amplification and/or spread of silencing signal. Genetic screens to search for the responsible factors identified RDR or RdRP as the major component responsible for the spread. But the most crucial and convincing biochemical evidence on the role of RDR in amplification of sRNAs during spread of silencing first came from the experiments of Lipardi et al. (2001); Sijen et al. (2001). Lipardi et al. (2001) showed that the siRNAs can also act as primers on ssRNA or dsRNA templates to continue polymerase-mediated chain reaction. In this way, a secondary set of dsRNA molecules appears, which eventually amplifies the siRNAs. This process can continue over several cycles of amplification, depending on the organism concerned. The amplified siRNAs are termed as secondary or transitive siRNAs.

Thus, the phenomenon of siRNA-mediated gene silencing involves both primary silencing and secondary or transitive silencing. In primary silencing, the trigger is an aberrant dsRNA that is processed into primary siRNAs mainly by DCL4. In transitive silencing, ssRNA templates are primed by primary siRNAs and are acted upon by RDR6 and SGS3 to produce dsRNA, which are processed by DCL2 or DCL4 to produce 22- or 21-nt transitive siRNAs, respectively (Nishikura, 2001), that degrade complementary mRNAs. The process can lead to the generation of siRNAs corresponding to sequences located outside the region of homology between the silencing inducer and the primary target, resulting in silencing of the secondary targets that are not homologous to the initial silencing trigger.

Historically, Schiebel et al. (1993a, b) showed that the purified RDR from tomato leaves catalyzed the synthesis of dsRNA from the primed or unprimed ssRNA template. Subsequently the RDR gene was successfully cloned (Schiebel et al., 1998), and a clear role of RDRs in the mechanism of silencing was established by the studies involving mutations in the putative homologs of RDR genes such as the *qde-1* gene of *Neurospora crassa* (Cogoni and Macino, 1999), the *sgs2/sde1/rdr6* gene of *Arabidopsis thaliana* (Dalmay et al., 2000; Mourrain et al., 2000; Yu et al., 2003), the *ego-1* and *rrf-1* genes of *Caenorhabditis elegans* (Smardon et al., 2000; Sijen et al., 2001), and the *rrpA* gene of *Dictyostelium discoideum* (Martens et al., 2002).

In plants, the phenomenon of transitivity was first observed when tomato plants transformed with *1-aminocyclopropane-1-carboxylate* (*acc*) *oxidase* (*aco1*) gene carrying an inverted repeat (IR) of the 5″ untranslated region (UTR) showed cosuppression of the transgene and endogenous *aco1* (Hamilton et al., 1998). In addition, it also silenced the endogenous *aco2*, which exhibited significant similarity to the transgene in the coding region. This implied a spread of silencing from the IR in the 5′ UTR to the upstream coding sequence (Hamilton et al., 1998). Subsequently, siRNAs corresponding to the region immediately upstream of the IR were detected (Han and Grierson, 2002). Another report demonstrated the spread of silencing in both 5′–3′ and 3′–5′ directions. It was described that the GFP-expressing *Nicotiana benthamiana* plants, bombarded with fragments complementary to 5′ (GF) or 3′ (P) of *gfp*, showed SS of the integrated *gfp.* These plants also exhibited silencing of nonoverlapping *gfp* sequences that were independently expressed from a potato virus X (PVX) virus–based vector (Voinnet et al., 1998), indicating that virus-induced gene silencing (VIGS) of transgenes is also associated with target-site spreading. It was also demonstrated that during cosuppression the endogenous gene was suppressed via amplification of silencing signal and target selection (Saunders et al., 2002).

The term *transitive silencing* was first adopted for *C. elegans*, after observing the presence of secondary siRNAs corresponding to regions upstream of the region targeted by the inducing dsRNA (Sijen et al., 2001). In an elegant experiment, Sijen et al. (2001) conducted RNase protection experiments to detect secondary siRNAs corresponding to muscle-specific *unc-22* or a germ line-specific *pos-1* gene. In one case, *unc-22-gfp* transgene and the endogenous *unc-22* gene were used as the primary and secondary targets, respectively. Worms injected with GFP-dsRNA exhibited the twitching phenotype indicating loss of *unc-22* expression. To examine whether the endogenous genes could act as primary and secondary targets, worms carrying deletion in one allele of *unc-22* were injected with dsRNA corresponding to the deleted region. This led to transitive silencing of both the wild-type (WT) and the deletion alleles. The abundance of these secondary siRNAs appeared to decrease as a function of the distance from the primary trigger. The presence of the primary target mRNA was essential for the transitive effect, and it targeted sequences located 5′ to the mRNA sequences homologous to the primary dsRNA (Alder et al., 2003).

To test whether these secondary siRNAs are capable of targeting degradation of homologous mRNAs, a transitive RNA-silencing assay was carried out by using two populations of target RNA (Sijen et al., 2001). The primary target consisted of nuclear-targeted green fluorescent protein (*gfp*)-*lacZ* fusion construct, and the secondary target was a mitochondrially targeted *gfp*. Worms carrying both transgenes, when injected with dsRNA segments from *lacZ*, showed reduction of both nuclear *gfp-lacZ* and mitochondrial *gfp*. It was observed that a trigger that was located 3′ to the *gfp-lac*Z junction was most potent in the assay. Finally, Sijen et al. (2001) also demonstrated the requirement for RRF-1 (homolog of RDR) in the generation of secondary siRNAs and detection of transitive RNA silencing.

In *Drosophila*, RDR primed with a synthetic 21-nt siRNA led to robust synthesis of a dsRNA of 690 bp in length in the embryo extracts (Lipardi et al., 2001). Additionally, it was shown that a broad range (22–40 nt) of short antisense RNAs (asRNAs) could also efficiently trigger RNA silencing in *C. elegans* when injected in close proximity to the target mRNA (Tijsterman et al., 2002). Consistently, modification of the 3′ ends of the asRNAs or siRNAs severely reduced their silencing efficiency (Lipardi et al., 2001; Tijsterman et al., 2002). Later, experiments on *N. crassa* provided the first evidence that purified recombinant QDE-1, a homolog of the plant RDR, possesses *de novo* and primer-dependent RNA polymerase activity that is required for the production of dsRNA. This leads to the production and amplification of siRNAs (Makeyev and Bamford, 2002; Catalanotto et al., 2004).

## Pathways for Formation of Transitivity and Their Roles

It is apparent that recruitment of RDR activity is an essential prerequisite for transitive sRNA synthesis. The underlying mechanisms that activate the production of transitive siRNAs are still not clear. Amplification of the RNA-silencing signal could occur either by replicating the dsRNA trigger or by expanding the initial pool of siRNAs. In order to support the latter possibility, it was proposed that, at specific cellular threshold concentrations, the antisense strands of siRNA may hybridize to the target mRNA and prime the RDR reaction. It was also likely that a fraction of newly synthesized dsRNA might be unwound by an RNA helicase activity or by RDR itself such that the sense and antisense strands of the newly synthesized dsRNA can be primed by the corresponding strands of siRNA, resulting an exponential amplification of not only siRNAs but also template RNAs (Lipardi et al., 2001).

The *Arabidopsis* genome encodes for six RDR proteins (RDR1 to RDR6) of which RDR1, RDR2, and RDR6 share a typical DLDGD signature motif sequence at the C-terminal catalytic site, and they belong to RDRα clade, whereas RDR3, RDR4, and RDR5 share an atypical DFDGD motif at the catalytic site, and they belong to RDRγ clade (Wassenegger and Krczal, 2006).

RDR6 synthesizes dsRNA by using target RNAs as template, either by priming of primary siRNAs (Lipardi et al., 2001; Sijen et al., 2001) or by a primer-independent mechanism that starts at the 3′ end of the target RNAs (Schiebel et al., 1993a; Vaistij et al., 2002; Tang et al., 2003; Petersen and Albrechtsen, 2005). The RDR2 is required for biogenesis of very diverse and abundant siRNA species involved in maintenance of genome integrity and transposon taming through heterochromatin formation (Xie et al., 2004). Recently, it was shown that RDR1 is also involved in the production of transitive virus-derived siRNAs and virus-activated siRNAs (vasiRNA) in the host (Cao et al., 2014). This process is adapted as antiviral defense to reinforce silencing.

The function of members of the RDRγ clade remains elusive, although there are few evidences suggesting their role in plant defense. The At-RDR3 expresses strongly at inflorescence apex, whereas At-RDR5 expresses uniformly in different parts of *Arabidopsis* plant (Willmann et al., 2011). RDR3 from *Salvia miltiorrhiza* was induced after cucumber mosaic virus (CMV) infection, suggesting its role in the antiviral silencing (Shao and Lu, 2014). Tomato *Ty1/Ty3* genes encode for RDRγ and are associated with antiviral resistance (Voorburg et al., 2020). Tomato cultivars with *Ty1/Ty3* displayed resistance against begomovirus and tomato yellow leaf curl virus and displayed enhanced viral siRNA (VsiRNA) generation and cytosine methylation of the begomovirus genome. However, these plants were susceptible to RNA virus-like CMV (Verlaan et al., 2013; Butterbach et al., 2014). This indicates that *Ty1/Ty3* locus may be involved in siRNA amplification required for TGS (specific for DNA genomes) rather than the PTGS pathway.

### RDR6-Dependent Transitivity in PTGS

Experiments using *rdr6* mutant suggested its role in the spreading and maintenance of the silencing (Vaistij et al., 2002; Himber et al., 2003). RDR6 contributes to the silencing pathway by initial signal perception (Melnyk et al., 2011b). In *Arabidopsis rdr6* mutant, which contain primary but not secondary siRNAs, the silencing could be initiated but not maintained.

The role of RDRs was initially investigated in *Arabidopsis* plants stably expressing GFP, in the background of functional and knocked-out *rdr6*. These plants were transformed with an IR corresponding to 5′ (GF) of *gfp* under the SUC2 promoter, such that movement of GFP silencing outside the phloem companion cell (CC) vasculature could be monitored. This transgene generated large amounts of both 21- and 24-nt siRNAs that complexed with AGO1 to cause PTGS of sense transgenes. These could also prime the aberrant transcripts to initiate a cascade of reactions in the RDR6 pathway to produce a host of secondary siRNAs. Some of the secondary siRNAs could be detected outside the incipient cells because of systemic spreading (Eamens et al., 2008). The plants with functional RDR6 were uniformly silenced, but in plants with mutated RDR6, the spread of GFP silencing affected only 10 to 15 cells beyond the veins. These plants contained primary but not secondary siRNAs, so the silencing could be initiated but not maintained. This clearly suggested RDR6 dependence for amplification of the transitive silencing signal in the recipient tissues (Vaistij et al., 2002; Himber et al., 2003). Similar experiments also identified the role for SDE3, a putative RNA helicase, in the same pathway.

Similar results were obtained in grafting experiments using transgenic plants expressing RNA-silencing constructs to knock down RDR6 (Schwach et al., 2005). When these plants were used as scions (or receivers) on a GFP-silenced rootstock (or inducers), SS spread was either completely abolished or restricted to veins. However, the systemic signal was transmitted to the scions indicating that RDR6 is necessary for the perception of the signal (Melnyk et al., 2011b).

RDR6 also plays an important role during VIGS by utilizing the VsiRNAs, derived from the stem–loop structures within ssRNA or viral dsRNA templates, thereby amplifying the VIGS response (Schwach et al., 2005; Wassenegger and Krczal, 2006). Mlotshwa et al. (2008) have elegantly demonstrated that the DCL2 (and not DCL4) protein plays a very dominant role in production of secondary siRNAs either from hp-transgene or sense-transgene. In plants, RDR6 plays a major role in producing special classes of transitive sRNAs from various loci, which will be discussed later.

### RDR2-Dependent Transitivity in PTGS

RDR2a along with NRPD1a and DCL3 was found to affect TGS via the heterochromatic sRNA pathway (Dunoyer et al., 2007; Smith et al., 2007). The NRPD1a encodes the largest subunit of the putative plant-specific DNA-dependent RNA Pol-IV, which might act as a silencing-specific RNA polymerase as its transcripts are converted into 24-nt siRNAs by the actions of RDR2 and DCL3 to direct DNA methylation (RdDM).

Grafting experiments involving *dcl3*, *ago4*, and *rdr2* mutants exhibited compromised transitive silencing, indicating that these proteins were required in the perception but not the production of the signal (Brosnan et al., 2007). Later, it was shown that *nrpd1a* mutants used as scions (or receivers) in grafting experiments also exhibited reduced amounts of 24-nt siRNAs and DNA methylation, indicating its role in the production of the silencing signal (Molnar et al., 2010). Another screen identified a role for Classy1 (CLSY1), an SNF2 domain–containing protein, besides NRPD1a and RDR2. CLSY1 contains a DNA-binding region, and mutations in this region affect the short range (SR, details in the following section) silencing (Smith et al., 2007). Involvement of, JMJ14, a histone-H3K4 demethylase-promoting non-CG methylation, in SR RNA silencing was also demonstrated. It was proposed that JMJ14 acts downstream to the AGO- effector complex to demethylate histone H3K4 at the targeted DNA (Searle et al., 2010). Recently, it was demonstrated that JMJ14 acts by reducing transcription levels of transgenes, thus preventing the triggering of S-PTGS (Masson et al., 2012). It was also shown that mutants for HUA enhancer 1 (HEN1), which is responsible for methylation of plant sRNAs, are defective in silencing spread. The heterochromatic sRNAs are very important components of TGS and will be discussed more in details later.

## Spread of sRNAs and Consequent Silencing

Silencing is a non–cell-autonomous event, initiated by a few dsRNA molecules (Hunter et al., 2006; Hinas et al., 2012) in one cell with eventual expansion of silencing of homologous sequences in a group of neighboring cells or even throughout the whole organism. The silencing even persists for a long time after the original source of silencing has been eliminated (Voinnet, 2005; Sarkies and Miska, 2014; Mermigka et al., 2015). Intercellular and long-distance movement of non–cell-autonomous RNA silencing involves mobile signals and various genetic components (Molnar et al., 2010; Melnyk et al., 2011b). The sequence specificity of the mobile signal indicated that it was nucleic acid, and later it was recognized as sRNA. This indicated that RNA transcripts could serve not only as targets but also as amplifiers of the initial silencing signal. These observations also highlighted the importance of sRNA amplification in defining the potency, transitivity, and propagation of RNA silencing.

The mobile nature of RNA silencing was first demonstrated by the identification of sRNAs in the phloem sap of cucurbits (Yoo et al., 2004). It was proposed that all classes of siRNAs (21–24 nt) are capable of movement; however, grafting experiments and agroinfiltration assays in GFP-expressing plants pointed toward 24-nt siRNAs as the probable systemic signal (Palauqui et al., 1997; Voinnet and Baulcombe, 1997; Himber et al., 2003; Dunoyer et al., 2010a; Molnar et al., 2010; Melnyk et al., 2011a). The DCL3-generated 24-nt siRNAs have been shown later to direct systemic TGS. A functional role of mobile siRNAs was suggested with the use of heterografts in *Nicotiana tabacum* plants (Zhang et al., 2014). Transgenic root stocks (lower part) expressing an IR of *disrupted meiotic cDNA 1* (*dmc1*), a meiosis-specific cell cycle factor, caused suppression of the gene in the anthers of the first flowers of the WT scions.

Silencing can manifest spontaneously up to approximately 10–15 cells from the point of initiation to form a zone of silencing (Himber et al., 2003; Schwach et al., 2005; Kalantidis et al., 2006; Dunoyer et al., 2007; Smith et al., 2007). In addition, plants show two types of transmission of the silencing signal. Depending on the pattern of silencing spread, it may include local or SR silencing and SS. SS spread requires a mechanical pathway for transmission as well as a mechanism to perceive and amplify the incoming signal.

### SR Spread of RNA Silencing

Short range spread of RNA silencing extends in a limited and defined area of cells beyond the silencing initiating zone. This generally involves limited cell-to-cell movement of the silencing signal in an apoplastic or symplastic manner. The movement across cells through cell walls and intercellular space is referred as apoplastic movement. It is likely that the plasmadesmatal channels are also used during the cell-to-cell spread (Lucas et al., 2009; Maule et al., 2011; Choudhary et al., 2019), and this is referred to as symplastic movement. This process does not require amplification of the silencing signal, and the host factors that assist in this kind of spread are still elusive. Recently, receptor-like kinases, known as BAM1/BAM2, have been identified from *N. benthamiana* and *Arabidopsis* (Rosas-Diaz et al., 2018), which assist in the spread of VsiRNAs (described later). It would be interesting to know the involvement of the same factor in spreading of siRNAs generated in other silencing pathways.

Short range silencing is induced mainly with transgenes, and initially, it was proposed that dsRNA might serve as the mobile silencing signal (Dunoyer et al., 2010a, b). Genetic screens identified the involvement of 21-nt siRNAs in SR silencing, indicating a pathway involving DCL4 and AGO1. The primary evidence toward this came from the demonstration of loss of SR spread in *A. thaliana dcl4* mutant lines. Complementation of *dcl4* mutants with DCL4 was able to reestablish SR signaling. The experiments also ruled out the role of other DCLs in spread of silencing over SR (Dunoyer et al., 2007; Smith et al., 2007). However, experiments using viral suppressors of silencing (VSRs) suggested the involvement of DCL3 processed 24-nt siRNAs (Hamilton et al., 2002). Later multiple findings supported the role of DCL4 produced 21-nt siRNAs as an SR signal (Dunoyer et al., 2010b).

Using biolistically delivered fluorescent 21- and 24-nt siRNAs, it was shown that both could spread locally to the anticipated range of 10–15 cells from the spot of insertion, but only 21-nt siRNAs lead to silencing outside the bombarded area (Dunoyer et al., 2010b). Expression of VSR, p19 of carnation Italian ringspot virus, which shows higher affinity for 21-nt over 24-nt siRNAs, prevented SR spread of silencing (Dunoyer et al., 2010b). The roles of 21- and 24-nt siRNAs in mobile PTGS and TGS are under deliberation (Sarkies and Miska, 2014). Recent studies in *Arabidopsis* pollen have led to the hypothesis that heritable epigenetic silencing of transposons may require the movement of 24-nt heterochromatic siRNAs from the vegetative nucleus to the sperm cells (Slotkin et al., 2009). Similarly, AGO9-dependent transport of transposon-derived 24-nt siRNAs out of the somatic CCs has been shown to play a crucial role in the specification of gametic cells by repressing transposon expression in the female ovules (Olmedo-Monfil et al., 2010). Mobile heterochromatic 24-nt siRNAs have been found to participate in the imprinting of paternal or maternal alleles during embryo development (Calarco et al., 2012).

Besides siRNAs, miRNAs and tasiRNAs (explained later) can also spread cell-to-cell. SR movement of miRNAs has been demonstrated in *Arabidopsis* (Brosnan and Voinnet, 2011). For instance, miR165/166 moves from the root endodermis to the central vascular cylinder, to trigger the degradation of the HD-ZIPIII transcription factor, PHABULOSA. This prevents the differentiation of protoxylem to xylem and ensures the correct development of the root cylinder (Carlsbecker et al., 2010). Similarly, miR394 moves from its expression zone, in the epidermal cell layer of the shoot apical meristem (SAM), to the internal meristem cell layers. This causes down-regulation of the F-box coding *leaf curling responsiveness* gene, to ensure the functionality of WUSCHEL, a homeodomain transcription factor required to promote stem cell formation (Benkovics and Timmermans, 2014).

The mobility of tasiRNAs has also been demonstrated during development of leaf polarity and lateral root growth (Chitwood et al., 2009; Schwab et al., 2009; Felippes et al., 2010; Marin et al., 2010; Benkovics and Timmermans, 2014). The evidence supports a role of miR390-mediated and TAS3-produced siRNAs for the regulation of auxin response factor 2 (ARF2), ARF3, and ARF4.

### Systemic Silencing

The transmission of silencing signal, leading to the suppression of the targeted gene in recipient cells (or sink cells), which are far away from the silencing originating cells (or source cells), constitutes SS. The first indication for SS came from the observations on spontaneous yet gradual propagation of PTGS from a localized area on a single leaf to the whole plant (Boerjan et al., 1994; Palauqui et al., 1996). Onset of silencing spread was initially in the veins of the systemic leaves, which gradually covered the whole leaf lamina. Further systemic spread of silencing was observed in acropetal direction from the lower silenced leaves to the upper non-silenced leaves, adopting mainly a phloem-based movement (Kehr and Buhtz, 2007).

Direct evidence for a SS was obtained by grafting experiments (Palauqui et al., 1997; Palauqui and Vaucheret, 1998; Voinnet et al., 1998). Grafting of the scion (upper part) of non-silenced plants onto the root stock of silenced plants has demonstrated that the silencing signal can be transmitted across a graft junction to induce silencing in the scion. Multiple lines of evidence support a symplastic movement of the SS signal from source to sink tissues. This involves cell-to-cell movement though plasmodesmata for reaching the phloem tissue, through which fast communication of distant organs is achieved. The nature of this SS mechanism was demonstrated by analysis of phloem sap content (Yoo et al., 2004; Buhtz et al., 2010; Rodriguez-Medina et al., 2011) and by assessing the movement of silencing signal across grafts (Palauqui et al., 1997; Voinnet and Baulcombe, 1997). In agroinfiltration assays using transgenic plants, it was observed that the silencing signal moved from mature leaves to young emerging leaves but not to fully expanded leaves (Voinnet et al., 1998). A relatively recent study has suggested that signal movement from roots to shoots in *Arabidopsis* might occur through plasmodesmata and not through the phloem (Liang et al., 2012), in a manner analogous to SR silencing spread. It was also observed that the root-to-shoot silencing spread expanded from the base to the tip of the leaf and not through the veins (Brosnan et al., 2007; Liang et al., 2012).

Signal amplification through the translocation stream is not an obligatory requirement for the long-distance movement of the SS signal. Suppression of the transgene in triple grafts, in which a WT stem separated a transgenic line serving as the inducer of silencing, and another as the recipient of the silencing signal (Palauqui et al., 1997; Voinnet et al., 1998) demonstrated that the silencing signal can move systemically even in the absence of homologous sequences in the recipient tissues.

*Arabidopsis* genetic screens were employed to understand the mechanism of spread in SS. In two independent experiments, the CC-specific SUC2 promoter was used to drive an IR fragment of target RNA, *phytoene desaturase* (PDS) and *sulfur* (SUL), respectively. The siRNAs were produced in the CC, whereas the chlorotic phenotype indicated SR spread of silencing (Dunoyer et al., 2007; Smith et al., 2007). Evidence supporting the involvement sRNAs in the establishment of SS in recipient cells also comes from studies using VIGS. It has been shown that viruses that are unable to move systemically and contain part of a host gene can induce silencing in systemic leaves (Voinnet et al., 2000; Kanazawa et al., 2010; Bond and Baulcombe, 2015), suggesting that a mobile signal produced during viral infection moves systemically to activate the antiviral mechanism in the recipient cells. This mechanism has been used to explain the cross protection against related viruses.

The different experimental approaches identified a role for DCL3 produced 24-nt siRNAs and the miRNAs in transmitting the SS signal over long distances. This was unequivocally demonstrated by grafting experiments involving tissues deficient in the biosynthetic pathway of 24-nt siRNAs (Melnyk et al., 2011a). Subsequent grafting experiments using the *dcl3* mutant background as inducers of the signal reported its requirement for the production of the signal (Melnyk et al., 2011a). It was also shown that DCL3 is necessary for the production but not the perception of the silencing signal, as WT silencing inducing scions could efficiently silence rootstocks carrying mutations in *dcl2, dcl3* and *dcl4* (Molnar et al., 2010; Melnyk et al., 2011a). Grafting experiments also suggested a role for NRPD1A and RDR6 in the spreading and maintenance of the silencing state (Vaistij et al., 2002; Himber et al., 2003).

The most accepted hypothesis suggests that DCL4 and DCL2 act hierarchically, as to produce 21- and 22-nt siRNAs, respectively, which guide the SS. Interestingly, DCL2 plays a central role in response to mobile signals for systemic PTGS in distal recipient cells. DCL2 expression in leaf vascular tissues enhances PTGS in surrounding cells in *Arabidopsis*. DCL2 produces 22-nt siRNAs that stimulate biogenesis of 21-nt secondary siRNAs (tasiRNAs) via RDR6 and DCL4, resulting in increased cell-to-cell spread of PTGS (Parent et al., 2014).

The systemic mobility of miRNAs such as miR399, miR395, miR172, and miR156 through the graft union has been observed in several studies (Lin et al., 2008; Pant et al., 2008; Buhtz et al., 2010; Kasai et al., 2010; Bhogale et al., 2013). Shoot-to-root translocation of miR395 in WT/*hen1* grated plants has been shown to down-regulate ATP sulfurylase 4 (Buhtz et al., 2010). In similar experiments, using miR399-overexpressing scions and WT stocks, shoot-to-root translocation of miR399 was shown to down-regulate PHO2, a critical component for Pi homeostasis (Lin et al., 2008; Pant et al., 2008). Grafting scions of transgenic plants overexpressing miR156 on stocks of WT potato on showed morphological alterations similar to those observed in miR156 overexpressing plants implying a role of miR156 as a systemic signal (Bhogale et al., 2013). Increased levels of miR395, miR398, and miR399 in both non-vascular tissues and the phloem sap have been measured during sulfate, copper, and Pi starvation, respectively (Buhtz et al., 2008; Buhtz et al., 2010). Further experiments are needed to assess if the increase in miRNA level in the phloem sap could regulate plant development.

Much less information is available on the form by which siRNAs and miRNAs move systemically. In cucurbits, a phloem protein, phloem small RNA-binding protein 1 (CmPSRP1), having high affinity to single-stranded sRNAs was identified (Yoo et al., 2004; Buhtz et al., 2008). However, genetic evidence supporting a role for CmPSRP1 in the transport of mobile systemic sRNAs is still lacking.

Mobile sRNAs transfer epigenetic changes in recipient tissues, through both PTGS and TGS pathways. PTGS-mediated establishment of silencing in the recipient tissues was reported by two independent studies (Brosnan et al., 2007; Liang et al., 2012), because they could detect cleaved transcript of the silenced targets but no epigenetic modifications in the coding region. Several other studies, however, detected changes in the methylation status of promoter or downstream regions in the recipient tissues, indicating the operation of a TGS-mediated pathway (Dunoyer et al., 2010a; Molnar et al., 2010; Bai et al., 2011; Melnyk et al., 2011b).

The silencing spread and consequent mobile RNA silencing are regulated by many internal and environmental factors, for example, hydrogen peroxide (H_2_O_2_). Using an elegant screen of *Arabidopsis* mutants impaired in the movement of root-to-shoot silencing, but not the production or effectiveness of the RNA-silencing signal, it was shown that the *rci3* gene, coding for H_2_O_2_, contributes to mobility of the silencing signal (Liang et al., 2014). The defect in mobile silencing in rci3 plants could be complemented by exogenous H_2_O_2_. However, there are several biochemical enzymes such as catalase, which can scavenge H_2_O_2_ and thus reduce mobility of silencing in WT plants. It was thus postulated that dynamic interaction between endogenous H_2_O_2_ and reactive oxygen species might control silencing spread by altering PD permeability through remodeling of local cell wall structure (Liang et al., 2014).

## Biogenesis and Function of Secondary siRNAs

### miRNA-Mediated Origin of Secondary siRNAs

RDR6 plays an important role in regulating leaf development and juvenile- to adult-phase transitions through the synthesis of endogenous tasiRNAs and phased siRNAs (phasiRNAs) that function by promoting cleavage of target transcripts (Peragine et al., 2004; Vazquez et al., 2004; Allen et al., 2005). Their biogenesis combines features and genetic requirements of siRNAs and miRNAs. The miRNA-mediated cleavage site determines the phase and is critical for the production of specific siRNAs (Allen et al., 2005).

#### tasiRNAs

##### Biogenesis

The 21-nt tasiRNAs are derived from non–protein-coding TAS transcripts that are capped as well as poly-adenylated and contain a binding site mostly for 22-nt miRNA. The tasiRNAs can methylate the TAS DNA but do not influence production of TAS transcripts (Wu et al., 2012).

The miRNA cleavage products are stabilized by suppressor of gene silencing 3 (SGS3) (Yoshikawa et al., 2005) and converted into a dsRNA form by RDR6 (Allen et al., 2005). The dsRNA intermediate is then processed by DCL4 and dedicated dsRNA-binding protein 4 (DRB4) to phased 21-nt siRNAs in a “head-to-tail” phased pattern. The transitive siRNAs are incorporated into AGO-RISC for targeting complementary sequences (Vazquez et al., 2004; Allen et al., 2005; Gasciolli et al., 2005; Yoshikawa et al., 2005). AGO1 and AGO7 proteins have been found to be associated with function of tasiRNAs (Peragine et al., 2004; Vazquez et al., 2004; Allen et al., 2005; Yoshikawa et al., 2005; Fahlgren et al., 2006; Montgomery et al., 2008a, b).

In *A. thaliana*, eight tasiRNA-producing loci have been identified that fall into four TAS groups (TAS1–TAS4). Among these, the TAS3 loci seem to be conserved in land plants. The tasiRNA production from TAS1a, b, c and TAS2 RNAs is initiated by the 22-nt-long miR173::AGO1 RISC and from TAS4 by the 22-nt-long miR828::AGO1 RISC (Montgomery et al., 2008a, b; Cuperus et al., 2010). A notable exception concerns TAS3, from which tasiRNA production is initiated by two 21-nt-long miR390::AGO7 RISCs. However only the 3′ proximal site of TAS3 can be cleaved, whereas the 5′ site does not have cleavage ability because of the higher degree of mismatch with miR390 (Axtell et al., 2006; Montgomery et al., 2008a). Interestingly the 3′ miR390 targeting site seems not to be essential for tasiRNA biogenesis because phasiRNAs can still be generated if other miRNAs replace miR390 at this site, as long as cleavage takes place (Montgomery et al., 2008a). The TAS3 5′ region is indispensable for triggering tasiRNAs because a change in this miR390-binding site to another miRNA entirely blocks secondary siRNA generation (Montgomery et al., 2008a). Another important requirement to trigger TAS3 tasiRNAs is the recruitment of miR390::AGO7 complex at the 5′ targeting site (Axtell et al., 2006; Montgomery et al., 2008a). This is believed to generate an intermediate aberrant poly (A)-less substrate that facilitates the amplification by RDR6 (Baeg et al., 2017).

Besides the four families of TAS genes in *Arabidopsis*, other TAS genes are reported in different plant species that spawn tasiRNAs by similar mechanisms. TAS5 was first reported in tomato, three TAS6 genes are reported in Moss, and TAS7-10 genes could be present in grapevine. The tasiRNAs from TAS7-10 genes are not well characterized, but all the non-coding TAS transcripts give rise to tasiRNAs in presence of the initiator miRNAs. Other than these, a few tasi-like sRNAs are also reported from rice (Deng et al., 2018).

##### Function

The TAS1 tasiRNAs target pentatricopeptide repeat containing genes (PPR), as well as few others (Allen et al., 2005), whereas targets for TAS2-tasiRNAs are exclusively the PPR genes. *Arabidopsis* has approximately 500 PPR genes, many of which are abiotic stress related; however, only a few are targeted by TAS1-tasiRNAs. The biological relevance of this selective regulation needs to be ascertained. Li et al. (2014) demonstrated that TAS1 is involved in thermotolerance of plants through regulation of several heat stress–related transcription factors. Heat treatment reduces TAS1-tasiRNA levels and consequently increases the levels of “heat-induced TAS1 target1 (HTT1)” and HTT2, thus enhancing thermotolerance of *Arabidopsis*. Similarly, other targets responsible for chilling tolerance were identified. It was observed that, at 4°C, TAS1-derived tasiRNAs accumulated in low amounts, and as result, expressions of the targets such as At151670, At4g29760, and At5g18040 were higher, which helped to cope up with the chilling effects (Kume et al., 2010).

The conserved TAS3-tasiRNAs control a wide spectrum of biology by targeting the ARFs. These are the transcription activators that mediate the control of developmental pathways by plant hormone auxin. The ta-siRNA-ARF module is also one among the most conserved sRNA-target regulatory pathways in plants being present in the simple organisms, such as liverworts and ferns, to monocots and eudicots (Xia et al., 2017). It is interesting to note that the number of TAS3 loci depends on the plant species and can range between two and hundreds, and this fact is a pointer to the long evolutionary history of miR390-TAS3-ARF cascades in distinct land plants.

In *Arabidopsis*, TAS3 can spawn at least nine tasiRNAs, and of these, two tasiRNAs target two ARFs, namely, ARF3 and ARF4. It is likely that other tasiRNAs might target other ARFs. This targeting of ARF3 determines the abaxial fate of *Arabidopsis* leaves. It was shown that the biogenesis of conserved but low abundant tasiRNAs targeting ARF3 is restricted to the adaxial side, by the localized expression of AGO7 and TAS3. This generates an ARF3 protein–deficient zones that marks the adaxial side. However, the processed tasiRNAs move from the adaxial side to the abaxial side of leaf lamina, thus creating a gradient of sRNAs and its target ARF3. As the targeting is incomplete, it still keeps ARF3 protein visible and detectable to pattern the abaxial determinant (Chitwood et al., 2009).

The tasiRNA-ARF module of *Arabidopsis* was shown to determine leaf morphology, flower and root architecture, developmental transition, embryo development, abiotic and biotic stress responses, phytohormone cross-talks, and so on. Any defects in the TAS3-tasiRNA biogenesis lead to aberrant floral morphogenesis and accelerated juvenile to adult-phase transition (Chitwood et al., 2009). Recently, it has been revealed that the same module is responsible for adaptations to extreme environments in several plants such as *Medicago truncatula*, *Lotus japonicus*, *Zea mays*, *Dimocarpus longan Lour*, and *Pyrus serotina* (Deng et al., 2018). The tasiRNAs-ARF module in moss *Physcomitrella patens* is responsible in auxin signaling and nitrogen sensitivities, implying that such module is coopted in lower plant evolution (Xia et al., 2016). The other targets of TAS3-tasiRNAs are found in *Bruguiera gymnorrhiza* (Wen et al., 2016) and are responsible for stress adaptation. Similarly, tasiRNAs are found to target AP2 transcription factors in bryophytes.

Approximately 5,047 active tasiRNAs are known in *A. thaliana*, and approximately 70% of them are non-canonical, which give rise to a phenomenon known as cascading effect. One prominent example could be drawn from at-TAS1c locus, which produces tasiRNAs initiated by the 22-nt miR173. One of the 22-nt tasiRNAs, named as “athTAS1c-D6(−),” targets not only its parent transcripts but also other unrelated ones to produce secondary phasiRNAs (Allen et al., 2005). The TAS2-3′D6(-) can target two PPR genes, namely, At1g12770 and Atg63130 (Allen et al., 2005). The cascading effect thus spawns secondary and tertiary phasiRNAs to expand the domain of complex control by a single initiator 22-nt miRNA (Vargas-Asencio and Perry, 2020). TAS2 transcripts harbor several short reading frames of peptides, which are translated and get associated with TAS2 transcripts in the polysome fractions. Such associations are speculated to enhance RDR6 amplification, resulting in higher accumulation of TAS2-tasiRNAs (Yoshikawa et al., 2016).

Approximately nine tasiRNAs are spawned by the TAS4 in *Arabidopsis* when triggered by 22-nt miR828, to down-regulate the *myb* genes. The most dominant tasiRNAs, namely, TAS4-tasiRNA 81(-), cleaves MYB-90 (PAP2), MYB-75 (PAP1), and MYB-113. All of these targets are involved in anthocyanin accumulation pathway and initiation of trichome in *Arabidopsis* leaves. Thus, these tasiRNAs act as a negative regulator of trichome initiation (Shi and Xie, 2014). Cotton MYB2D/MYB2A genes are also regulated by miR828 (as well as miR858), and these genes also spawn tasiRNAs, which inhibit cotton fiber production and *Arabidopsis* trichome (Guan et al., 2014). Similar miR828-TAS4 module is reported to be present in several dicots (but not in monocots), and its presence also controls trichome development in apple (Zheng et al., 2015).

#### Phased Transitive siRNAs

The dsRNAs produced from TAS or other loci can also be diced by DCL2 enzymes in collaboration with SGS3 and RDR6 to initiate 22-nt phased transitive siRNA production. However, such siRNAs might have larger cascading effects. These are also called non-canonical tasiRNAs, and they in turn can target their parent mRNA in-*cis* or in-*trans* mRNAs to initiate synthesis of further rounds of dsRNAs. In this way, many genes can be coregulated simultaneously by one initiator miRNA, and such effects are known as cascading effects. It is interesting to note that *dcl1/dcl4* and *dcl1/dcl3/dcl4* plants grow poorly in greenhouse conditions, but *dcl1/dcl2/dcl4* and *dcl1/dcl2/dcl3/dcl4* plants are healthy and viable. It is speculated that in *dcl1/dcl4* and *dcl1/dcl3/dcl4* genotypes, the DCL2 might overproduce 22-nt siRNAs, which could then trigger massive overproduction of secondary siRNAs (cascading effects), resulting in explosive posttranscriptional silencing and subsequent poor growth of plants (Chen D. et al., 2010).

As another interesting exception, the regulation of auxin signaling homeostasis was found to depend on a network of sec-siRNAs, termed siTAARs (Windels and Vazquez, 2011), which are processed after 22-nt miR393b guided cleavage of the TIR/AFB2 auxin receptor (TAAR). The siTAARs were shown to act in-*cis* on their own source transcripts as well as in*-trans* on homologous TAAR transcripts and on unrelated transcripts (Si-Ammour et al., 2011). The siTAARs were shown to be important for specific aspect of leaf development, but their other roles need to be investigated.

#### PhasiRNAs

The 21-nt (or 24-nt) phasiRNAs are also generated from protein coding as well as other genes with the help of initiator miRNAs following mechanisms similar to biogenesis of tasiRNAs. In fact, tasiRNAs could be deemed as a subset of phasiRNAs. However, phasiRNAs act in-*cis*; i.e., these can degrade the progenitor mRNAs and control varieties of activities in plants. PhasiRNA-producing genes are collectively called “PHAS” genes. Generally, PHAS genes occur in clustered families, and phasiRNAs arising from a member might target all the family members as well as unrelated genes. Most of the land plants and lower plants code for phasiRNAs that control several biological pathways. Most of the “PHAS” loci are hit by 22-nt miRNAs, but only a few of them require 21-nt miRNAs, which hit twice in the PHAS genes (Feng et al., 2019). According to an old estimate, 23 plant species are found to encode 3300 PHAS loci, among which 1,600 are protein-coding genes. The rest are non-coding introns, intergeneic loci, transposons, etc. (Zheng et al., 2015). The phasiRNAs that are spawned from these loci are responsible for plant development (Guan et al., 2014; Guo et al., 2018), proper functioning of reproductive tissues (Zhang et al., 2020), defense responses (Wang T. et al., 2018; Hou et al., 2019), and abiotic stress tolerance (Sosa-Valencia et al., 2017).

The NBS-LRR motif–containing “R” genes, which protect plants against pathogens, are probably the largest family of protein–genes that spawn the 21-nt (or 24-nt) phasiRNAs. These sRNAs negatively control expressions of the R genes in normal (uninfected) conditions to avoid the associated fitness cost. The *Arabidopsis* miR825-5p targets MIST1 gene in the sequence coding for a highly conserved functional amino acid motif (TIR2) within the TIR domain of the receptor. As a result, *trans*-acting phasiRNAs are generated that, in turn, down-regulate a wide network of TIR-NBS-LRR (TNL) genes. Regulation through *MIST1* affects disease resistance against the bacterial pathogen *Pseudomonas syringae* (Buscaill et al., 2020). For biogenesis of phasiRNAs, the *Medicago* NBS-LRR resistance genes are hit by 22-nt miRNAs, namely, miR1507, miR2109, and miR2118 (Zhai et al., 2011). Similarly, citrus resistance genes are targeted by miR472 and miR482 (Song et al., 2012a), whereas the soybean NBS-LRR genes are controlled by miR482, miR1507, miR1510, etc. (Zhao et al., 2015a). It was interesting to note that although miR2109 is present in both *Medicago* and soybean, it works only in *Medicago* but not in soybean. The reason was attributed to its size as *Medicago* miRNA is 22 nt, but soybean one is only 21 nt. Thus, the same miRNA differing in length can have different functions through distinct mechanisms (Deng et al., 2018). The phasiRNAs do not allow expression of NBS-LRR genes in the absence of pathogens, but their levels drop following pathogen invasion as the initiator miRNA becomes limiting in amount, resulting in expression of R proteins that protect plants against infections.

The phasiRNAs were identified in diverse set of dicot species but not in monocots such as rice, indicating presence of different mechanism of pathogen resistance in monocots. This may also be attributed to the differential diversification of NBS-LRR genes between dicots and monocots (Zhang et al., 2020), as indicated by the presence of TIR domain in most dicot proteins, whereas it is absent in monocots. Interestingly, very few miRNAs were known to slice the NB-LRR genes in monocots, until the recent identification of several miRNA families, such as miR9863, miR3117, miR3084, miR5071, and miR7757, in wheat. These were found to target NBS-LRR transcripts and trigger the production of phasiRNAs (Zhang et al., 2020). It was interesting to note that miR2118 in eudicots is involved in plant immunity response, whereas in monocots, miR2118 plays an important role in anther development. The variation in miR2118 function could be attributed to the target sequence (TS) variation (Zhang et al., 2020).

The PPR genes are perhaps the second largest family to give rise to phasiRNAs. The apple and grapevine PPRs are targeted by miR7122 to spawn phasiRNAs. The soybean and *Medicago* PPR transcripts spawn secondary siRNA cascades, which are generated by an initial cleavage by miR1509 (Xia et al., 2017). The MYB genes are the third largest family that spawns miRNA-triggered phasiRNAs. In apple, soybean, and cotton, the miR828 and miR858 target MYB motifs directly to produce phasiRNAs, which ultimately control MYB transcription factors. Such controls are relevant in secondary metabolism, seed development, and cotton fiber growth. The F-box proteins encoding SCF (i.e., S phase kinase-associated protein1–Cullin–F-box) ubiquitin ligases are another large family that generates phasiRNAs to control numerous biological processes (Deng et al., 2018). One-third of strawberry F-box genes are targeted by a 22-nt miRNA to generate a phasiRNA network that contributes to strawberry flower and fruit shape (Xia et al., 2015).

To have appropriate levels of RNA silencing, the silencing factors should be controlled in their expressions. In this context, the phasiRNAs derived from the transcripts of the factors such as DCL, SGS3, RDR, and AGOs render great biological activities in the system-specific modes. The loci of phasiRNAs have been pinpointed to DCL2 in *Medicago* and soybean (triggered by miR1507 and miR1515 in *Medicago* and soybean, respectively), SGS3 in peaches and soybean (triggered by miR2118 in soybean), and AGO2 in peaches. Reports of DCL-derived 21-nt phasiRNAs from papaya, sweet orange, tomato, and tobacco; AGO-derived 21-nt phasiRNAs from *A. thaliana*, *Arabidopsis lyrata*, monkey flower, tomato, and sorghum; and RDR-derived 21-nt phasiRNAs from foxtail millet and *Amborella trichopoda* are available in literature (Zheng et al., 2015). Thus, the phasiRNAs may work in a feedback mechanism to control expression of RNA-silencing genes, and this mechanism may be prevalent in a wide variety of land plants.

In addition to the genes mentioned above, some low-copy gene transcripts are also progenitors of phasiRNAs that play a very important role in plant development. These PHAS loci include those encoding wound response proteins, hormone response factors, transcription factors, proteins involved in signal transduction, transporters, protein translation machinery components, photosystem components, histone and DNA methylation proteins, the cytoskeleton and associated factors, intracellular trafficking machinery, kinases, and other enzymes involved in diverse metabolic pathways (Zheng et al., 2015). PhasiRNAs have been located from auxin signaling pathways, namely, from the transcripts of transport inhibitor response (TIR) and one of three genes of auxin receptor F-box (AFB2) where miR393 acts as an initiator miRNA. Similar to TIR/AFB, NAC domain–containing transcription factor transcripts also yield phasiRNAs in citrus and *Litchi* using the miR3954, and the roles of these phasiRNAs are implicated in flowering regulation (Liu et al., 2017).

So far, emphasis has been laid on eudicot phasiRNAs, and monocot phasiRNAs have been occasionally mentioned. The available data indicate that monocot-derived phasiRNAs play a great deal of roles in anther and inflorescence tissue formation. In grass family, two pathways form abundant phasiRNAs that are associated with meiosis. The miR2118 triggers one class of 21-nt phasiRNAs in premeiotic anther development, whereas miR2275 triggers another class of 24-nt phasiRNAs, which are required for pollen development (Zhai et al., 2015). The same miRNAs are also involved in generating allohexaploid wheat phasiRNAs. Zhang and colleagues have identified abundant phasiRNAs in the reproductive tissues such as young spikes and anthers, whereas very few loci from leaf, stem, root, spikelet, seed, etc., were found to generate phasiRNAs (Zhang et al., 2020). Inflorescence tissue–derived phasiRNAs are also reported. In rice inflorescence, 828 and 35 (of 21 and 24 nt) PHAS loci have been identified that produce 21- and 24-nt phasiRNAs, respectively. The number of PHAS genes is also dependent on the rice varieties. In maize, 463 and 176 of 21-PHAS and 24-PHAS loci are identified. In the flower of *Litchi*, 178 of 21-PHAS loci are detected. These generation and regulation mechanisms are much conserved in grasses (Zhang et al., 2020).

A recent report describes that *Cuscuta*, an obligate parasitic plant that absorbs water and nutrients from the host plants, also accumulates high levels of 22-nt miRNA. These miRNAs can target *Arabidopsis* and tobacco mRNAs and produce secondary siRNAs (Shahid et al., 2018). In this way, *Cuscuta* suppresses gene expression and can parasitize in hosts, suggesting that phasiRNAs provide an important mechanism of *trans*-species gene regulation. Collectively, the phasiRNAs are emerging as very important regulators of plant biology.

A class of 24-nt phasiRNAs has been recently discovered during the reproductive stage in rice, and a DCL3 protein, DCL3b, rather than DCL4 processes these sRNAs for their biogenesis. Their precursor dsRNAs are amplified by the SGS3 and RDR6 module as canonical 21-nt phasiRNAs (Johnson et al., 2009; Song et al., 2012b, c; Komiya, 2017). The phasiRNAs from TEs, identified in the vegetative nucleus of pollen grains, in dedifferentiated plant cell cultures and in DNA methylation mutants, are proposed to provide an alternate pathway to posttranscriptionally silence TEs, to allow them to evade long-term heterochromatic silencing (Creasey et al., 2014).

### *cis*-Natural Antisense Transcript siRNAs

#### Biogenesis

Many plant loci transcribe from both strands of genomic DNA, i.e., transcribe in opposite directions and a substantial fraction (95%) of such pairs form hybrids (H), which are termed as 3′-3′ H, 3′-5′ H, or enclosed H (Zhang et al., 2012). Such pairs are known as *cis*-natural antisense transcript siRNAs (*cis*-NATS). In *Arabidopsis*, 9% of the whole genome is devoted for making *cis*-NATS, and almost all eukaryotic genomes encode *cis*-NATS. Each component of *cis*-NATS is coregulated, and their transcriptions are either environmentally or plant-developmentally controlled (Axtell, 2013). The mechanisms for gene regulation by *cis*-NATS are of four different types, and one of them involves siRNA formation predominantly from one of the strands (Zhang et al., 2013). Approximately 6% and 16% of the *cis*-NATS in *Arabidopsis* and rice, respectively, are engaged in siRNA biogenesis, respectively. Thus, only small portions of *cis*-NATS are controlled by RNA-silencing machinery.

The enzymes such as DCL1 and DCL3 dice the hybrids of RNA in 21- and 24-nt siRNAs, respectively, and a subset of those 21-nt are further amplified by RDR6, whereas most of the 24-nt are amplified by RDR2. In the generation of siRNAs, the role of Pol IV has also been demonstrated. Many times, the siRNAs are reported to be generated from introns also and are thought to be generated in the plant nucleus (Zhang et al., 2012). Some nat-siRNAs regulate the expression of their cognate NAT mRNAs in-*cis*. In *Arabidopsis*, DCL1 regulates more siRNA-associated *cis*-NATS (23.5%) than total *cis*-NATS (7.9%). This fact is strongly suggestive of down-regulation of sRNA producing *cis*-NATS by DCL1-dependent nat-siRNAs. However, DCL3-dependent nat-siRNAs may not be directly involved in the expression regulation of the NAT transcripts (Zhang et al., 2012).

#### Function

The functions of the majority of siRNAs of *cis*-NATS are not assigned yet, although the roles of a few of those reveal that they work in stress alleviation and plant development. The first siRNA reported for functionality was from the *Arabidopsis* NAT-pair of *deltapyrroline-5-carboxylate dehydrogenase* (*p5cdh*) transcript and *similar to radicle induced cell death one 5* (*sro5*) transcript. Salt treatment of plant induces *sro5* mRNA, which forms dsRNA at its 3′ end with the constitutively expressed P5CDH transcript. The DCL-processed siRNAs, namely, nat-siRNA-sro5, is further amplified by RDR6 and Pol IV. This siRNA then directs the cleavage of *P5CDH* transcripts, resulting in reduction of proline degradation and increase in salinity tolerance (Borsani et al., 2005). Similarly, the bacteria *P. syringae* DC3000 strain carrying an effector gene *avrRpt2* induce the *ATGB2* transcript from a GTP-binding protein gene that base pairs with the constitutively expressed antisense transcript of PPR-like protein (PPRL). The resulting siRNA, namely, nat-siRNAATGB2, the biogenesis of which is dependent on DCL1, RDR6, SGS3, and NRPD1 (the largest subunit of Pol IV), down-regulates PPRL in its turn. As PPRL is the negative regulator of RSP-mediated resistance, siRNAATGB2 derepresses the pathway of effector-triggered immunity (Katiyar-Agarwal et al., 2006).

Besides these, the role of *cis*-NATS in regulation of plant developmental gene expression has also been clearly demonstrated in the case of down-regulation of ARI14 whose expression in sperms inhibits the fertilization of plants. Similarly, the *cis*-NATS coming out of the overlapping region of the sense and antisense transcripts of the Shooting (*sho*) gene locus in *petunia × hybrida* can be found in all tissues except roots, thus allowing the *sho* gene to direct cytokinin synthesis in the correct root locations (Zhang et al., 2013). Other reports of functions of the natsiRNAs associated with plant development and environmental cues can be found in literature (Chen H. M. et al., 2010; Zhang et al., 2012).

### Heterochromatic siRNAs

The predominantly 24-nt heterochromatic siRNA (hcsiRNA) or *cis*-acting siRNAs promote DNA or histone modifications at the loci that generate them (Xie et al., 2004). These siRNAs correspond to several endogenous silent loci, including retrotransposons, 5S rDNA, and centromeric repeats and also to genomes of extrachromosomal elements such as viruses and virods (Chan et al., 2005; Rosa et al., 2018). The hcsiRNAs are methylated by HEN1 and act through a complex similar to the RNA-induced transcriptional silencing complex, RITS. This complex likely contains AGO4 as indicated by the mutants’ phenotypes, which overlap with those of *rdr2*, *dcl3*, *nrpd1a*, and *nrpd2* (Zilberman et al., 2003). The RDR2–DCL3–NRPD1–AGO4 pathway has clear roles in silencing transposons for maintaining the genome integrity in plants (Zilberman et al., 2003; Xie et al., 2004) and heterochromatinization of centromeric repeats (Volpe et al., 2002). The 24-nt siRNAs can also act to silence intronic transposons as exemplified by the silencing of FLC (flowering control locus C), the key negative regulator of flowering (Liu et al., 2004).

Most of the hcsiRNAs are secondary siRNAs as their biogenesis is highly dependent on RDR2 and Pol IV. Although classically the 24-nt siRNAs are used to be regarded as the ones associated with these kinds of secondary siRNAs, plenty of 21/22-nt siRNAs have been identified for the similar functional activities in *Arabidopsis* (Zhao et al., 2016). Approximately 80% of all sRNAs are hc-siRNAs, and thousands of such unique siRNAs are present in *Arabidopsis*. The participation of various factors in their biogenesis and mechanism of action have been described in many reviews (Axtell, 2013; Won et al., 2014; Rosa et al., 2018).

The hcsiRNAs act in-*cis*, and these can cause epigenetic modifications also, in “*trans*,” depending on the sequence similarities of the target DNA. The processes of establishing methylation and their maintenance by specific enzymes resulting in TGS have been well described (Won et al., 2014). The expansion of RdDM in the region downstream of primary methylation in the *Arabidopsis* genome that occurs in stepwise pathways involving 24-nt secondary siRNAs has also been reported (Daxinger et al., 2008). Chromatin methylation can also occur where p4-siRNA mediates the process (Hudzik et al., 2020).

The hcsiRNAs are very important in maintaining genome integrity and gene regulation as they guide epigenetic modification at repeats. The latter elements occupy large portions of the plant genomes (Lisch, 2009), and some of them can jump to other regions or can amplify themselves, resulting in disruption of functional genes. But plants have evolved several protective mechanisms to stop such mobilization of transposons. The hcsiRNA-mediated epigenetic modifications come handy to prevent such mobilizations. This hypothesis has been supported by facts that the loss of function of RdDM pathway mutants cause derepression of the expression of transposons and repeats. Similar derepression has also been observed with loss of MET1 and DDM1 function. Such depression in *Arabidopsis* displays various sorts of phenotypic abnormalities such as delayed flowering, stunting, and sterility.

Sometimes, repeats are also located in the promoter regions of protein-coding genes that generate 24-nt sRNAs. These regulatory elements are controlled by RdDM, and their methylation level affects the expression of nearby genes (Won et al., 2014). Because of the on and off status of transcription of these loci, depending on absence and presence of methylation, respectively, these loci are also called epialleles. One such epiallele is the suppressor of ddc (*sdc*) locus with a direct tandem repeat in its promoter. Presence of methylation results in the WT phenotype, whereas absence of methylation causes the expression of the locus, resulting in the dwarf phenotype. Another example comes from the epialleles of flowering locus T (FT) where methylation of two enhancers located 5 kb upstream and 1 kb downstream of the gene can repress FT expression and results in delayed flowering phenotype. The third example of flowering Wageningen (FWA) locus, which determines correct flowering time, has been widely worked upon. It has two tandem SINE3-like retroposon repeats at its promoter, and transcribing sequence remains highly CG methylated and transcriptionally inactive throughout the vegetative development. FWA gets activated only in female gametophyte and endosperm by maternal imprinting. The FWA locus also is active in FWA mutant where hypomethylation occurs in the promoter of locus, resulting in delayed flowering (Srikant et al., 2019).

The hc-siRNAs contribute greatly in the reproductive growth of the plants. The methylation programs of the gamete cells of both types and their CCs differ in an opposing manner. Similar differences are also observed between endosperm and the zygote (Castel and Martienssen, 2013). The male gamete cells contain two sperm cells and one enlarged vegetative cell as the CC, whereas the female gamete contains the egg cell, central cell, and five accessory cells. The double fertilization of the egg cell and the central cell leads to the formation of embryo (zygote) and the endosperm. As CCs, the accessory cells and the vegetative cell support the development of their adjacent cells, namely, the egg cell and the sperm cells, respectively. Similarly, the endosperm supports the development of the zygote (Won et al., 2014). DDM1 and MET1 enzymes are underrepresented in the vegetative cell and the nursing, respectively, causing global decrease in cytosine methylation. In the CCs of both gametes, DEMETER (DME), an active demethylase enzyme, further reduces the level of methylation through the demethylation of methylated cytosines. Such hypomethylation results in derepression of transposons and expression of 24-nt siRNAs. These siRNAs get transported in the gamete cells, reinforcing silencing of transposons to protect the genomic integrity of gametes. Similarly, the transposons of endosperms are also mobilized because of lack of methylation, giving rise to 24-nt hc-siRNAs, which are transported, in turn, to zygotes. This process protects the genomic integrity of zygotes, which can be passed down to the next generation without any harm (Won et al., 2014).

## Biological Features of Secondary siRNAs

### Transgene Silencing

Silencing of transgenes is required to keep host genomic integrity against the invasive DNA elements, so the transgenes are made silent both at the TGS and PTGS levels, although the exact mechanisms are not fully understood (Guo et al., 2016). In instances when multiple copies of the sense transgenes are inserted in the plant chromosomes, Pol IV generates aberrant transcripts from such arrays that are then channeled through the RDR2 and DCL3 pathway to trigger TGS, by RdDM of transgenes and their promoters (Eamens et al., 2008). Besides Pol IV, Pol II can also generate the aberrant transcripts of sense transgenes, but these transcripts are channeled in a route requiring RDR6, SGS3, DCL4/DCL2, and AGO1 to initiate PTGS in the cytosol (Eamens et al., 2008; Martínez, de Alba et al., 2013). The details of RDR6 and RDR2 pathways have been discussed in *Pathways for Formation of Transitivity and Their Roles*. This transgenic PTGS can be inhibited by some of virus-encoded suppressors of RNA silencing such as P19, P38, and P15 proteins from tomato bushy stunt virus (TBSV), turnip crinkle virus (TCV), and peanut clump virus, respectively (Moissiard et al., 2007).

### Virus-Induced Gene Silencing

The VIGS response is compromised in *rdr6* or *sgs3* mutant backgrounds as against a robust response seen in the *rdr6*^+^ and *sgs3*^+^ background of the host (Muangsan et al., 2004; Vaistij and Jones, 2009). When PVX-based VIGS vector lacking the movement protein (MP) is introduced in plants, the vector gets localized, but the silencing signal remains systemic (Voinnet, 2005). The usage of VSRs has also indicated differences in the manipulation of silencing. The VSR proteins such as P19, P38, Hc-Pro (*turnip mosaic virus*) could strongly reduce the production of secondary siRNAs, whereas P15, P25 (PVX), and so on, could not affect secondary siRNA production (Moissiard et al., 2007). These evidences point toward a strong role of secondary siRNAs in VIGS-mediated silencing. Movement defective mini-geminivirus carrying host gene such as PCNA is efficient in carrying out VIGS of the corresponding host gene throughout the plant. This indicates that mobility of PCNA-siRNAs (most of which are probably secondary siRNAs) across the plant is good enough to silence the replicative host protein PCNA in the absence of any virus movement (Pasumarthy et al., 2011).

### Transitivity and Systemic Spread of siRNAs in Silencing

The transitive siRNAs amplify the amplitude of silencing (PTGS) in the incipient cell and also get transported to systemic regions to cause silencing (PTGS) in the recipient cells. The mechanism of cell-autonomous RNA silencing (CARS) is well established, but the mechanism of non–cell-autonomous RNA silencing (non-CARS) is still emerging (Zhang et al., 2019). In CARS, DCL4 plays a major role over DCL2, but in non-CARs, DCL4 plays an inhibitory role, whereas DCL2 plays the super role in SS. The dcl4 mutant plants are more efficient in systemic spread than the WT, whereas dcl2 mutants fail in systemic spreading (Vazquez and Hohn, 2013). In the incipient cells, DCL2 facilitates RDR6-mediated silencing process and for the systemic spread DCL2 acts as a receiver of silencing signal in the recipient cells (Taochy et al., 2017). Taochy et al. invented a novel screening method to identify mutants that fail to transmit PTGS signal from root to plant shoot and thus identified the dcl2 mutant of *Arabidopsis*. They also observed that dcl4 rootstocks generated more DCL2-dependent 22-nt siRNAs than the wild *Arabidopsis* and showed enhanced systemic movement of PTGS to the grafted shoots (Taochy et al., 2017). Besides DCL2, several cellular factors including SNF2, a JmjC domain protein JMJ14, and the THO/TREX mRNA export complex are found to be associated with intercellular non-CARS. It is noteworthy that amplification of signals such as siRNA (mostly 22-nt) is also essential for transmission of cell-to-cell RNA silencing in *Arabidopsis* (Zhang et al., 2019). But in solanace plants such as tomato, *Nicotiana*, etc., systemic spread might involve two different mechanisms (Vazquez and Hohn, 2013).

The transitive siRNAs can also be used to silence the endogenes. Van Houdt and colleagues employed a three-tier XYZ transgenic system and showed that the expression of transcripts (tertiary target, Z) bearing no homology to the silencing-inducing locus (primary target, X) can also be decreased dramatically via transitive RNA silencing (Bleys et al., 2006). This phenomenon requires some homology between primary target RNAs and secondary target RNAs (Y). Sequences upstream from the region homologous to the silencing inducer in the primary target transcripts give rise to approximately 22-nt sRNAs, and these target secondary transcripts for silencing. Similarly, the sRNAs emanating from the homologous region between the secondary and tertiary RNAs (Z) can silence the Z transcripts, whereas the primary (X) and tertiary transcripts (Z) will have no homology at all.

Using the similar system, it was shown that the length of sequence homology determines the frequency and efficiency of endogene suppression by transitive silencing signals (Bleys et al., 2006). Others have also used this system in *Nicotiana* and concluded that transitivity-mediated silencing can affect endogenes only to a limited extent, but transgenes can be silenced at will with much higher efficiency. Vermeersch et al. (2013) showed that the transitive siRNAs can methylate the cytosines of transgene DNA but fail to do so on the endogenes. Transitive RNA silencing has also been used to do targeted forward mutagenesis in *Arabidopsis*. This approach can be employed to target a subset of the transcriptome in order to identify genes responsible for a particular localized process, such as photosynthesis (Petsch et al., 2010).

A few facts of transitivity are still hard to explain with certainty. For example, endogenes cannot be as easily silenced as transgenes using transitivity. A possible speculation is the high transcription rate of transgenes, thus generating more aberrant transcripts and siRNAs. But endogenes fail to do so. Another guess could be that endogenes contain introns, but transgenes are cDNAs, devoid of introns. In support of this hypothesis, Christie et al. introduced an intron into a transgene, which resulted in suppressing the strength of silencing of the transgene in splicing-dependent manner. Thus, intron splicing could be a suppressive factor for transitivity-mediated silencing (Christie et al., 2011). Another fact to consider is that the transitivity in the 5′ to 3′ direction is more frequent than the same in the 3′ to 5′ direction. This preferential direction of transitivity could lie in the nature of RNA fragment resulting from the initial dicing. If the slice point is within the 5′ UTR and coding region of the RNA, the 5′ diced fragment might not be a good substrate of RDR6 because of the presence of scanning and translating ribosome. But the 3′ RNA fragment will be free for RDR6-mediated extension, generating transitive siRNA from that region only and not from the other half (Molnar et al., 2010).

### Transspecies Transfer of Secondary siRNAs

Plants exchange sRNAs with the invading pathogens and pests. Here we would stick to transfer of siRNAs only. In the majority of events, such siRNAs are in the form of secondary siRNAs. Plants transport siRNAs in pathogens to down-regulate their mRNAs to boost defense against pathogens. Pathogens, in their turn, weaken host defense by channeling pathogen-specific siRNAs in the hosts.

The two of the tasiRNAs derived from *Arabidopsis* TAS1 and TAS2 non-coding genes target the fungal genes involved in vesicle trafficking in *Botrytis cinerea*, resulting in resistance of *Arabidopsis* to the fungus *B. cinerea* (Cai et al., 2018). When *Arabidopsis* is invaded by *Phytophthora capsici*, a few of the pentatricopeptide-repeat protein (PPR) genes produce a pool of siRNAs, which are transported to the fungus and inactivate the fungal genes responsible for pathogen development and colonization (Hou et al., 2019). The PPR gene–derived siRNAs have also been predicted to target genes in another fungal pathogen, *Verticillium dahliae*, indicating that these PPR genes can confer broad-spectrum resistance to a wide variety of pathogens (Hudzik et al., 2020). Consistent with the notion that secondary siRNAs are at work in transport between plants and pathogens, the rdr6-mutants of *Arabidopsis* exhibit hypersusceptibility to fungal pathogens such as *B. cinerea*, *P. capsici*, and *V. dahliae*.

The reverse flow, i.e., transport of siRNAs from pathogens to plants, has also been evidenced in many cases. The fungus *B. cinerea* accumulates several siRNAs following plant infection. A few of these siRNAs match the transcripts of host immunity genes in a complementary manner and down-regulate host immunity (Weiberg et al., 2013). These siRNAs require host AGO1 to exert their functions, and accordingly, the *Arabidopsis* ago1 hypomorphic mutant is quite resistant to *B. cinerea*. On the flipside, *Arabidopsis* also becomes resistant to the dcl1/dcl2 mutant of *B. cinerea*, which fails to generate the siRNAs. The oomycete *Plasmopara viticola* is predicted to generate siRNAs that can silence grapevine (*Vitis* species) mRNAs during infection (Brilli et al., 2018).

The mechanisms of *trans*-kingdom transfer of sRNAs are being intensively researched now. The role of extracellular vehicles from the donor to the recipient cells has been suggested by many investigators (Hudzik et al., 2020). It is noteworthy that not all pathosystems can cause *trans*-kingdom RNA silencing, but a vast majority of them do. Thus, the secondary siRNAs are extremely important tools not only to cause RNA-silencing to the systemic regions of the same plant but also to execute *trans*-kingdom RNA silencing.

## Applications of Secondary siRNAs

Based on the aforementioned features, many different gene silencing tools have been developed. The plasmid vectors for sense/antisense transgene silencing, dsRNA/hairpin RNA constructs and virus vectors derived from RNA and DNA viruses for inducing host chromosomal gene silencing (VIGS) have been adequately described in literature, and these also have been extensively used for engineering agronomic traits in crops (Eamens et al., 2008; Guo et al., 2016). Here, we briefly mention a few others.

### Transitivity-Based Silencing Constructs

In this approach, the target transgene gene for silencing (say X) is placed upstream of an element bearing an IR of 3′ UTR having heterologous sequence. The IR element is transcriptionally fused with X gene. It is better that a splice-able intron is sandwiched between the repeats. This cassette is then introduced in a binary vector and made ready for transformation in a plant system bearing the target gene X as a resident one. When introduced in plant, siRNAs are generated from the IR using the plant dicing activities. These siRNAs are then extended toward the 5′ end of the X-transcripts making use of transitivity. The average processivity of such extension by RDR6 enzyme is approximately 750 nt. In this process, the dsRNAs of X-transcripts are generated, which in turn are diced further, eventually destroying the transcripts of both transgene and endogene X. This transitivity-based approach is an efficient silencer but is weaker in efficiency compared to the hp-RNA–based constructs.

Filichkin et al. (2007) have successfully silenced three genes of *Arabidopsis*, namely, *ap1* (encoding a MADS domain transcription factor that specifies floral meristem identity), *ettin* (encoding ARF3), and *ttg1* (encoding a WD40 repeat protein regulating trichome and root hair development) using the transitivity-based vector. They have used the IR of octopine synthase (*ocs*) terminator as a source of heterologous siRNAs in their transitivity vector. They have also found that hp-RNA vectors are superior to transitivity vectors in silencing the target genes (Filichkin et al., 2007). Nizampatnam and Kumar (2011) produced male sterile tobacco plants by overexpressing open reading frame (ORF) H522 (sterility inducer) in the tapetum cell layer, and they also restored the sterility by silencing the sterility inducer gene by transitivity vector. Similar application has also been made in strawberry fruits (Härtl et al., 2017). Thus, using this approach, mutant of any gene of a plant, which is amenable to transformation, can be made.

### Artificial tasiRNAs/MIGS Vectors

These vectors generate secondary siRNAs directly and are not dependent on induction of primary siRNAs. The *Arabidopsis* TAS DNA sequences can be engineered to silence sequences of interest. Some of the phased tasiRNA-producing sequences of TAS DNA can be replaced by single or multiple siRNAs of different sequences but of equivalent length of base pairs. When the replacing siRNAs are processed in the tasiRNA pathway from the engineered vectors as desired, these are called the artificial tasiRNAs (atasiRNAs), which consequently silence their targets in a usual manner using the RNA-silencing machinery of the host plants.

The engineered TAS construct expresses the TAS transcript containing antisense target gene sequences in the first place, and the hybrid transcript gives rise to phasiRNAs to target the cognate gene of interest. These atasiRNAs have been shown to be effective to induce silencing of endogenous genes (de la Luz Gutiérrez-Nava et al., 2008; Montgomery et al., 2008a, b). The *TAS1c* locus in *A. thaliana* was engineered by de la Luz Gutierrez-Nava et al. to silence the *FAD2* gene by replacing a single native tasiRNA with an siRNA targeting *FAD2* (siFAD2) or replacing five native siRNAs with siFAD2. Such strategies showed silencing phenotypes akin to that of a fad-2-1 null mutant. Multiplexing of several atasiRNAs can also lead to simultaneous silencing of several related or unrelated genes. The construct retaining the binding site of miR173 but including a large part of gene of interest by removing the bulk of TAS1c sequences is also effective in inducing silencing. The atasiRNAs can be computationally designed with user-friendly web tools such as P-SAMS^1^ (Fahlgren et al., 2016) so that these are highly specific and do not generate the so-called off-target effects. These days, the atasiRNA constructs have emerged as a powerful tool for studies in plant biology and overall crop improvement (Zhang, 2014).

Not all 22-nt miRNAs or siRNAs are the progenitors of tasi-/phasi-RNA pathways. The reasons for selection of a few particular progenitor sRNAs to spawn the tasiRNAs are not clear at the moment. When any gene (say X) is placed under the control of such peculiar miRNAs and allowed to transcribe in plants, phased 21-nt siRNAs (phasiRNAs-like) are generated, and the silencing constructs harboring the mentioned elements are known as miRNA-induced gene silencing (MIGS) vectors. The MIGS term was first coined by de Felippes and colleagues when they used the 22-nt miR173 binding site upstream of many *Arabidopsis* genes separately and showed elegantly the loss of function of the endogenous genes. They used cDNA fragments of AGAMOUS (AG), EARLY FLOWERING 3 (ELF3), FLOWERING LOCUS T (FT), and LEAFY (LFY) and demonstrated that the MIGS constructs were capable of mimicking the phenotypes *ag2*, *elf3-9*, *ft-10*, and *lfy-12* null mutants (Felippes et al., 2012). When the hybrid cDNAs were used in MIGS constructs, the silencing of the gene closet to miR173 was most predominant. Many plants such as tobacco do not encode miR173; hence, these types of plants need to be cotransformed with the *Arabidopsis* pre-miR173 gene along with the MIGS constructs to achieve the silencing effects of corresponding genes. In *Petunia*, CHS (chalcone synthase) and PDS gene-silencing phenotypes were achieved when the *Arabidopsis* miR173 precursors were cotransformed along with the MIGS constructs. Here the mature miR173 was processed in a manner different from *Arabidopsis*, and both phased and out-of-phase siRNAs resulted in the plant tissues. Silencing of the targeted endogenes was very efficient, but the molecular mechanisms of silencing between *Arabidopsis* and petunia plants were different (Han et al., 2015). Similar miR173 coexpression with MIGS transgenes has been reported in *M. truncatula* (Imin et al., 2013), soybean (Jacobs et al., 2016), and rice (Zheng et al., 2018). Despite having been widely used, the MIGS approach is beset with a significant risk of off-target effects due to (i) the numerous tasiRNAs being generated from the MIGS construct, (ii) the generation of out-of-phase siRNAs from MIGS constructs as observed in *Petunia*, and (iii) the possibility that MIGS-derived tasiRNAs can induce transitivity as reported (Han et al., 2015). Finally, loading of MIGS-derived tasiRNA into particular AGOs cannot be controlled at all as various tasiRNAs will have various 5′ ends (Carbonell, 2019).

### Host-Induced Gene Silencing

Previously, we have seen that plants transport secondary siRNAs in invading pathogens and pests such as fungi, nematodes, insects, etc. Although the specific mechanism of transfer is not fully revealed, this principle can be exploited to develop antipathogen strategies. The siRNAs, which can specifically and crucially target developmental genes of pathogens, can be expressed in plants in a transgenic manner. When these siRNAs are passed on to the invading organisms, they inactivate the target pathogen genes using the RNA-silencing machineries of the pathogen, thus crippling the growth of pathogens.

This principle has been successfully utilized first to target and inactivate the root-knot nematode; *Meloidogyne incognita*. The nematode secretes a small signaling peptide, namely, 16D10, in the plant cells that causes pathogenicity by promoting accelerated and enlarged root growth. Huang et al. expressed dsRNA as well as hp-RNA of 16D10 in *Arabidopsis* and challenged the roots of transgenic plant by *M. incognita*. As a result, the numbers of galls and *M. incognita* eggs on transgenic *A. thaliana* plants were greatly reduced because of reduction in secretion of peptide (Huang et al., 2006). Expression of dsRNA in cotton that targets the cytochrome P450 gene in cotton bollworm (Mao et al., 2007) significantly reduces bollworm infestation (Baum et al., 2007). Similarly, the dsRNA designed to inactivate a vacuolar ATPase gene in coleopteran insect pests are expressed in maize to protect the plant against the insect pests *Helicoverpa armigera* (Baum et al., 2007). Host-induced gene silencing (HIGS) is also good in controlling parasitic weeds (Hudzik et al., 2020).

Host-induced gene silencing has been extensively employed to control the fungal and oomycete pathogens. Increased resistance against hemibiotrophic oomycete pathogen *Phytophthora infestans* was successfully achieved by silencing the targeted genes in potato plants that were allowed to express siRNA from a hairpin construct (Jahan et al., 2015). The HIGS approach of expressing siRNAs in plant has been used against various plant fungal pathogens, viz., *Puccinia striiformis*, *Uromyces appendiculatus*, *Blumeria graminis*, *Fusarium oxysporum*, *Fusarium graminearum*, *Fusarium culmorum*, *Bremia lactucae*, *Verticillium dahlia*, *Rhizoctonia solani*, and *Sclerotinia sclerotiorum*, which cause important diseases in various crop plants (Singh et al., 2020). Recently, we have also have demonstrated prevention of wilting of tomato plants by *F. oxysporum* by expressing dsRNA of the fungal-specific ODC gene in tomato. This fungal pathogen is totally dependent on the ODC gene for its own polyamine biosynthesis that is used by *Fusarium* for its growth and development. Plants and other organisms have salvage pathways for polyamine synthesis, but the fungal pathogen lacks the salvage pathways (Singh et al., 2020).

A non-transgenic expression of dsRNAs targeted to pathogens and pests in plants can also be achieved through virus-based vectors. This approach may be called virus-induced HIGS and has been successfully used to silence plant pathogens and insect pests in a number of different plant species (reviewed in Ghag, 2017). The fungus *U. appendiculatus* causes rust disease in common bean, and this disease is widespread internationally. But common bean is hard to transform. Recently, bean pod mottle virus (BPMV) is made recombinant with 258-bp-long sequence of the rust fungus, and subsequently common bean was allowed to infect with recombinant virus followed by challenge with the fungus. The 258-nt RNA contained five *U. appendiculatus* candidate effector mRNAs. Four of the five effectors targeted rust disease symptoms, and the BPMV-infected beans showed less rust disease symptoms and less of fungal RNA. The virus-based HIGS has also been applied to resist insects of various kinds and has been summarized well by Rosa et al. (2018).

### Silencing by Externally Applied dsRNA

Expression of dsRNA in plants is usually executed by agrotransformation or viral routes. But the products of such procedures do not often meet consumers’ approval, and the regulatory bodies always encourage genetically modified organism (GMO)-free, virus-free procedures. Fortunately, plants internalize externally applied dsRNA and process those as siRNAs to effect silencing. Externally applied dsRNA, hp-RNA, siRNAs, etc., have been shown effective to cause silencing of endogenes and transgenes of plants, as well as genes of invading organisms such as viruses, fungi, insects, nematodes, etc. (Dubrovina and Kiselev, 2019). The exogenously applied RNAs can spread locally and systemically within plant, get transported into the pathogens, and induce RNA interference–mediated plant pathogen resistance. As the external silencing agents need to cross the cellulose-rich tough cell wall, which pose physical barrier to the entry in cellular compartments, and they themselves are not very stable following external applications on plant organs, various procedures such as tagging RNA with cell-penetrating peptide like (KH)_9_, conjugating RNA with various forms of polymer-nanoparticles, or bombarding dsRNA using an airgun, etc., are adopted.

The first report of silencing a plant gene, namely, PDS, with bleached leaves was made by Sammons et al. (2011). They sprayed (at 2.5 bar) with *in vitro* transcribed 685-bp dsRNA and/or chemically synthesized 21-nt sRNAs targeting the endogenous PDS on the leaves of *N. benthamiana* plants, which were pretreated with Silwet L-77 surfactant. As a result, the endogenous PDS mRNA displayed extensive PDS RNA silencing (Sammons et al., 2011; Dalakouras et al., 2019) with a clear bleached leaf phenotype. After this, several similar reports emerged. The recent studies showed encouraging results in imparting resistance in plant against plant viruses, fungi, and insects following exogenous application of various types of dsRNA and siRNAs. The RNAs were directly delivered in to the plant leaves by spraying or rubbing or through nanocarriers; additionally, trunk injection and root or petiole absorption also have been shown as the successful mode of delivery of dsRNA or siRNA in the woody and herbaceous plants (Dubrovina and Kiselev, 2019). Several studies also showed the down-regulation of plant transgenes and endogenes by exogenous RNAs conjugated with nanoparticles or a protein carrier (Dubrovina and Kiselev, 2019).

Not all sRNAs can cause transitive and SS following application in plant leaves. Dalakouras et al. found that only 22-nt sRNAs are capable of SS, whereas others cause only local silencing (Dalakouras et al., 2019). The 22-nt sRNAs can recruit the amplification machinery (RDR6-like enzymes) to the ss-RNA template, resulting in the biogenesis of secondary siRNAs, and the population of siRNA builds up to a certain threshold level that is required for the onset of SS. Thus, transitivity is seemingly connected to generation of SS signals in the source tissues. The reception of silencing signal in the sink tissues is also dependent on RDR6 processing, and the silencing spread is efficient on the target gene devoid of any introns. Taking these facts into consideration, it can be postulated that systemic RNA silencing, upon exogenous application of ds-RNAs, may significantly increase when the trigger is 22-nt sRNA, and the target gene is intronless (Dalakouras et al., 2019).

Since its inception in 2011, the field has expanded enormously generating lots of mechanistic questions, which are yet to be answered. The delivery of the external silencing agents is cost-effective and less time-consuming and does not involve the tangles of GMO. The success of such types of silencing can overcome the red flags waived by regulatory bodies and can also meet consumers’ consent. The success and failures of the use of ds-RNA applications on plants to checkmate viruses, viroids, fungi, insects, mites, nematodes, etc., have been nicely reviewed in many articles (Rosa et al., 2018; Dalakouras et al., 2019; Dubrovina and Kiselev, 2019). The external application of RNA silencing agents has huge promise, but in order to make field-level success against the pathogens, huge amount of RNA materials will be needed. There are a few companies who are engaged in producing fermentor loads of bacteria and yeast to meet such demands. For delivery of dsRNAs in trunks of plants in industrial scale, many innovative drilling machines are also being discovered. Finally, the external silencers suffer from less of off-target effects compared to any other silencing techniques known so far (Dalakouras et al., 2019). In short, scientific community is optimistic about industrial success of dsRNA applications in plants.

## Viruses and Secondary siRNAs

Approximately 1,500 viruses are known to infect numerous plant species (Sastry et al., 2019), and the global annual loss due to plant viral diseases is tantamount to trillion dollars. Hence, the knowledge of virus infection process is important for the management of viruses. Viruses have diverse genetic makeup, and they are classified into positive-sense RNA, negative-sense RNA, double-stranded-RNA viruses, and single-stranded or double-stranded DNA viruses. The differences in the genome organization reflect differences in the strategies of replication, systemic spreading, and handling host RNA-silencing processes. In plants and other organisms, the silencing response is always associated with amplification of the responses, which are taken care of by the host encoded RDRs. The amplified response causes RNA silencing to spread from the originating cell to the neighboring cells in a systemic manner.

The virus-infected plant cell defends itself from virus by generating siRNAs from all over the viral genome that are known as VsiRNAs. The viral transcripts are generally processed by host RNA-silencing machinery. First, the transcripts are converted to dsRNAs by a variety of mechanisms including the involvement of RDRs. The other silencing proteins, i.e., Dicers, namely, DCL4/DCL2/DCL3, dice the viral dsRNAs in 21-, 22-, and 24-nt siRNAs, respectively, in collaboration with their cognate RNA-binding proteins. The 21/22-nt VsiRNAs form RISC complexes and eventually slice the viral transcripts resulting in protecting the hosts from the viral infection. These primary VsiRNAs are also amplified by host RDR1/RDR6 proteins to generate a huge pool of secondary VsiRNAs that spread from the incipient to the distant recipient cells. This spread occurs presumably to prevent the viral infection front from advancing. The viruses have also evolved to counterprotect themselves by encoding proteins, named as RNA-silencing suppressors. Depending on the nature of viral suppressors, they are capable of inhibiting RNA-silencing reactions at every stage of RNA silencing. Such defense and counterdefense have been reviewed in several occasions (Agrawal et al., 2003; Csorba et al., 2015; Sanan-Mishra et al., 2017), and here, we will focus on few facts associated with secondary VsiRNAs.

### Mobility of VsiRNAs

It is well-established by now that the 22-nt siRNAs are in the center of intercellular spread, whereas other siRNAs are generally localized in their activities (Dalakouras et al., 2019; Zhang et al., 2019). Hence, spread of antiviral RNA silencing is presumably mediated by 22-nt VsiRNA. This spread is influenced by many host factors, as well as viral factors. The host factors such as ALA1, ALA2, and AVi2, etc., help both in biogenesis and spread of VsiRNAs (Guo et al., 2018). It has also been demonstrated that DCL4 inhibits the process of spreading, whereas DCL2 promotes spreading (Zhang et al., 2019). One more factor that is localized in plasmodesmata, namely, Bam1 and its homolog Bam2, acting as the receptor-like kinase, is a positive regulator of systemic spread of RNA silencing. The Bam1 activity, in turn, is negated by C4 RNA-silencing suppressor protein of TYLCV. The TYLCV-C4 protein complexes with the intracellular domain of Bam1 at the plasma membrane or plasmodesmata and interferes in the VsiRNA-mediated silencing spreading activity (Rosas-Diaz et al., 2018). Interference or blockage in the spread of antiviral spreading helps virus move from cell to cell.

Plant viruses employ dual strategies for their own survival: they encode proteins to promote spread of RNA silencing, and they have also evolved to encode RNA-silencing suppressors to prevent systemic spread of VsiRNAs. The TYLCV-C4 protein is an example that prevents spread of VsiRNA. Besides, there are many other similar proteins that prevent antiviral immunity. The TGBp1 protein is a part of a triple-gene block of MPs of potato virus M (PVM). This protein has been shown to inhibit antiviral silencing spread (Senshu et al., 2011). Prevention of systemic spread of VsiRNAs is a form of viral strategy to weaken host defense and allow establishing infection of the host. On the other hand, there are MPs of other viruses that help promote spread of antiviral silencing. Tobacco mosaic virus (TMV) encodes an MP of 30 kDa. This protein has been shown to promote spreading of antiviral RNA silencing (Vogler et al., 2008). Promoting spread of VsiRNA will keep the TMV viral titer low in the recipient cells, which are necessary for cellular health. The presence of high viral titer will implicate cellular death, resulting restricted persistence of the virus. Thus, promoting spread of anti-TMV silencing helps sustain the viral population in the host cells (Vogler et al., 2008).

### Role of Secondary VsiRNAs in VIGS

There are several evidences that point out that without formation of secondary VsiRNAs, the silencing efficiency of VIGS is either minimal or nil. Vaistij and Jones (2009) compared the efficiency of VIGS-mediated silencing while using the *Potexvirus* PVX and the potyvirus plum pox virus (PPV) as the vehicle of VIGS in the WT and *rdr6*-deficient *N. benthamiana* plants. The VIGS efficiency was seriously dampened in *rdr6* mutant plants compared to the WT, although the accumulation of VsiRNA was as similar in *rdr6* mutants as that in the WT. It was concluded that the high accumulation of primary siRNAs of PVX and PPV in *rdr6* mutant plants was ineffective in causing VIGS-mediated silencing (Vaistij and Jones, 2009). The VIGS mediated by begomoviruses provides supporting evidence. Aregger et al. (2012) showed that the begomovirus, cabbage leaf curl virus (CaLCV), VsiRNAs of all three size classes are mostly derived from primary siRNAs, and RDR6 does not contribute in accumulating secondary VsiRNAs (Aregger et al., 2012). This finding can be explained by the observations made by our laboratory. We showed that the begomoviruses: mungbean yellow mosaic India virus (MYMIV) and tomato leaf curl New Delhi virus (ToLCNDV)–encoded AC2 protein interacts with and inactivates RDR6 (Kumar et al., 2015), and thus perhaps nil or very little secondary VsiRNAs can accumulate in presence of the begomovirus-AC2 protein. However, the silencing efficiency of begomovirus-VIGS increases several folds with the introduction of null mutation in the AC2 gene (Pandey et al., 2009). Thus, it appears that secondary VsiRNAs contribute to effective and enhanced silencing. The VIGS-mediated silencing by citrus leaf blotch virus holds up similar notion. The viral vector is almost equally permissive in *N. benthamiana* and citrus plants. But the VIGS-mediated silencing efficiency in *N. benthamiana* is much weaker compared the same in citrus. It appeared that the silencing weakness was caused by lesser accumulation of secondary VsiRNAs in tobacco (Agüero et al., 2014).

### Changes in the PhasiRNAs Profiles Following Virus Infection

Host miRNA profiles are often changed in the virus-infected plant cells. The virus-encoded RNA-silencing suppressors are the major contributory factors in this process. As a result, the PhasiRNA-initiator miRNAs (22 nt) are also altered following virus infection. Some miRNAs are overexpressed, resulting in overproduction of PhasiRNAs, whereas in other cases, PhasiRNAs disappear altogether. Such alterations lead to fine-tuning of the host genes related to resistance, susceptibility, or tolerance toward the infecting virus (Vargas-Asencio and Perry, 2020). Zheng et al. (2015) examined the PhasiRNA loci in uninfected papaya and papaya ringspot virus (PRSV)-infected papaya. Approximately 40 and 93 PHAS loci were found in healthy and infected leaf libraries, respectively. Among them, 13 were shared, whereas many others showed different expression patterns. Six disease resistance PHAS loci showed reduced production of 21-nt siRNAs upon PRSV infection. ARF3 suffered the highest degree of total siRNA reduction in infected leaves, and two auxin signaling F-box genes (AFB2 and TIR1/AFB) produced four times less siRNAs in infected leaves. Similarly, in the rice infected with rice stripe virus (RSV) and rice dwarf virus (RDV) and *Arabidopsis* plants infected with turnip mosaic virus (TuMV), a large number of PHAS loci showed substantial changes in PhasiRNAs following viral infections. The ARF gene in rice showed more than 8- and 3.5-fold changes in phasiRNA generation upon RSV and RDV infections, respectively. The authors suggested that viral infection-induced or -suppressed expression of selective phasiRNAs may act as a novel mechanism to regulate the expression of selective host genes (Zheng et al., 2015). In another example, changes in phasiRNAs of tomato infected with potato virus Y (PVY) were examined. Approximately 500 phasiRNA-generating loci were differentially expressed, and the phasiRNAs were shown to be mostly active in PVY-infected tissues at 21 dpi. These data demonstrated that phasiRNA accumulation mostly regulates R- and disease-responsive genes. The phasiRNAs accumulation dropped at 21 dpi, suggesting the sRNA-mediated defenses in the recovery phenotype (Prigigallo et al., 2019). Similar examples can also be found in *Arabidopsis* plants infected with CMV (Vargas-Asencio and Perry, 2020).

## Silencing Pathways in Plant–Virus Interactions

### Recovery From Virus Disease Symptoms

Earlier studies of virus-infected plants revealed that in some cases of compatible plant–virus interactions, plants recover from disease, and the emerging upper leaves of the plants become asymptomatic, whereas the lower leaves remain systemically infected. The asymptomatic leaves are immune to superinfection by the same or similar viruses, which caused the initial systemic infection but are quite susceptible by viruses that are unrelated with the first inoculum. This phenomenon was first described by Erwin Baur in 1906, who reported recovery of *Abutilon* plants from the infective variegation by a virus, which is known today as *Abutilon* mosaic virus (AbMV) (Pennazio et al., 1999). Later on, a plethora of reports appeared supporting the observation of recovery phenomena (Wingard, 1928; Ratcliff et al., 1999). Later part of these studies showed adequately that the recovery or the acquired immunity against the viruses was RNA sequence based. In 1929, McKinney reported another specific antiviral response in tobacco and showed that plants infected with mild forms of TMV, which caused light green mosaic, can resist super infection by more virulent forms of the same virus causing yellow mosaic (McKinney, 1929). This phenomenon subsequently gave birth to the process of biotechnological use, named as cross-protection. As with recovery, these plants remained sensitive to subsequent infections by unrelated viruses. Thus, in both cases (recovery and cross-protection), there was a specific response toward the infecting virus, and the observed immunity was homology dependent (Rosa et al., 2018). Until the end of the past century, the mechanism of these two apparently different phenomena remained obscure. But by now, it is well established that both processes are linked to induction and spreading of antiviral RNA silencing in a non–cell-autonomous manner. The recovery phenotype may not be used directly for control of virus disease but serves as a great resource for unveiling the mechanistic pathways of antiviral silencing. Here we would focus on the recovery process.

The experiments with transgenic plants overexpressing CP of TEV provided the first hint that RNA silencing is at work for plant recovery (Lindbo et al., 1993). Such transgenic plants when challenged with TEV are supposed to be virus-resistant according to pathogen-derived resistance (PDR) postulate. But the plants showed initial systemic disease symptoms and later recovered. This kind of symptom recovery was also found with the untranslatable viral genes (Dougherty et al., 1994). Later on, it was revealed that high expression of transgenes in plant cytoplasm leads to recovery phenomena, whereas very low level of expression ensures high resistance against viruses. The *in vitro* and genetic analyses with the recovered leaves showed that PTGS-mediated degradation of viral mRNAs occurs frequently, resulting in lowering of viral titer in the recovered leaves. Both VsiRNAs and viral genomic RNAs exist in low quantum, and thus, the disease expression is minimized (or nil) in recovered leaves (Ghoshal and Sanfaçon, 2015). However, in some cases, for example, infection of ToRSV in tobacco, the recovered asymptomatic leaves do not lose viral titer, but the translation of viral RNA template is inhibited (Ghoshal and Sanfaçon, 2015). Both the processes of PTGS and TGS have also been shown to occur in the symptom recovery associated with DNA viruses, such as CaMV and geminiviruses (Ghoshal and Sanfaçon, 2015). The virus-encoded RNA-silencing suppressors also contribute a lot to the recovery, and it is in fact a product of fine balance between RNA silencing and its suppression. Recovery can be reversed by ectopic expression of suppressors such as PVY-Hc-Pro, PVX-P25, etc. It implicates that the suppressor activity is kept low in recovery, and the recovery-type RNA viruses cannot counteract RNA silencing. The latter notion is consistent with the observation that certain non-recovery type of viruses shows recovery characteristics when they are made deficient of their suppressors. Supporting examples could be found in geminiviruses lacking the AL2/AC2 protein, potyviruses with mutations of HC-Pro, cucumoviruses with deletions of 2b, and tombusviruses with mutations or deletions of p19 (Ghoshal and Sanfaçon, 2015). In summary, the recovery process establishes an adequate equilibrium between the host antiviral RNA silencing and viral counterdefense mechanisms so that the disease expression is nil or minimal despite the presence of viral titer, albeit in a low quantity.

In a recent recovery report of a RNA virus, namely, oil rapeseed mosaic virus infecting *Arabidopsis* plants, participation of various antiviral silencing pathways was examined, and the detailed mechanisms of recovery process were revealed. Following viral infection, few leaves were systemically infected, and asymptomatic upper leaves at later stages of infection were observed. A couple of intermediate leaves known as transition leaves exhibiting characteristics of symptoms at leaf tip and edges, but disease-free features at the base of leaves, was also observed. The level of viral RNA of the recovered leaves was almost similar to that of the systemically infected leaves, but the activities of RNA-silencing suppressor proteins vanished altogether in recovered leaves, while the same in symptomatic leaves were highly prevalent. Upon ectopic delivery of RNA-silencing suppressor in recovered leaves, the recovery was reversed, indicating that loss of suppressor activity was the crucial determinant of recovery. The recovery process depended on the 21- to 22-nt siRNA-mediated PTGS pathway and also on some components of TGS pathway involving non–cell-autonomous silencing signaling. The symptom recovery was independent of biogenesis factors of miRNA and tasiRNA pathways and also did not depend on hormone signaling (SA/JA/ethylene, etc.) or siRNA-dependent RdRM pathways. However, the *Arabidopsis* mutants of secondary VsiRNA-degrading enzymes, namely, xrn4-3 and ein-5, showed enhanced recovery, supporting the role of secondary VsiRNA in recovery. Both RDR2 and RDR6 along with SGS3 and DCL4 were very much necessary for recovery. Taking all observations together, it appears that recovery process establishes a state of tolerance in the infected tissues and occurs following robust mobility of antiviral secondary siRNAs from source to sink tissues. The recovery also establishes a sufficient dosage of VsiRNAs that can block the VSR activity involved in the formation of disease symptoms (Kørner et al., 2018).

### Shoot Apical Meristem Exclusion of Viruses

The apical growing points are the strong photosynthetic sinks and hence the viruses are supposed to accumulate at SAM. However, most of the viruses are excluded from SAM barring a few, and these SAM-breaching viruses are eventually seed-borne. There are no general exclusion principles, but a majority of the reports claim that the viruses cannot enter SAM because of RNA silencing. There are some viruses such as TMV, which cause apical necrosis, and these are highly inhibited by overexpression of RDR1 or RDR6. The RDRs amplify silencing signals and thus stop those viruses to enter SAM, thereby establishing the key role of RNA silencing in SAM exclusion of viruses (Lee et al., 2016).

Even with RNA silencing, there are two different pathways for meristem exclusion. For viruses such as TRV, CMV, ToRSV, etc., meristem exclusion and recovery processes are concomitant. These viruses possess weak RNA-silencing suppressor activity, which helps the viruses to transiently invade SAM and initiate antiviral RNA silencing. This silencing requires host enzymes for amplification and eventually excludes the viruses from SAM in the long run. The amplified antiviral silencing also descends in the leaves that are derived from virus-free SAM, and as a result, these leaves show signs of recovery (Martín-Hernández and Baulcombe, 2008; Ghoshal and Sanfaçon, 2015). For CMV, CP also helps RNA silencing indirectly. CP has an arginine-rich zone and thus binds viral RNA tightly near SAM and neighboring leaves. This binding eventually recruits proteins such as RDR6, SGS3, etc., causing the viral templates to convert in dsRNA forms, which are diced producing antiviral sRNAs. Thus, CMV-CP enhances RNA silencing and causes SAM exclusion. The arginine less CP mutants of CMV can invade SAM easily and cause visible reduction in apical dominance (Zhang et al., 2017). On the other hand, the story with PVX and artichoke Italian latent virus (AILV-V, genus *Nepovirus*) is different, and here the meristem exclusion and recovery are quite independent of each other. The exclusion of PVX involves a silencing mechanism that initiates in lower uninfected tissues or systemically infected leaves and spreads ahead the viral infection front and depends on a long-range RNA-silencing signaling regulated by host RdRP RDR6 (Schwach et al., 2005). The recovery from PVX is also dependent on spreading of silencing and accumulating in the recovered leaves before viral infection can get there. Thus, recovery is not dependent on SAM-initiated antiviral silencing. An apparently similar process also occurs with AILV-V virus infecting tobacco. AILV-V invades SAM at 7 dpi and stays on until approximately 40 dpi. As this invasion occurs with a very low quantity of the virus, the RNA silencing–mediated response does not get sufficient time for its build-up. However, the top emerging leaves recover earliest at 21 dpi. For recovery to happen, viral RNA accumulates to a threshold level, which triggers the overexpression of RDR6 and DCL4 to initiate and maintain the antiviral silencing. Subsequently, the viral RNA decreases in the systemically infected leaves, and the gradual decrease above these leaves brings down the viral titer at the lowest concentration in the recovered leaves. Here AILV-V entry in SAM and activation of RNA silencing (RS) are two distinct processes as RS is triggered in fully expanded leaves after viral RNA reaches the threshold level and not by any RS build-up following virus entrance in SAM (Santovito et al., 2014).

The meristem exclusion of *Cymbidium* ringspot virus (CymRSV) infecting *N. benthamiana* highlights an altogether different pathway. By carefully inactivating the antiviral silencing machinery, the Burgyan group showed that the virus cannot enter tobacco SAM at all, thereby implicating that RNA silencing is not behind the SAM exclusion of CymRSV. The authors investigated the transcriptional changes following virus infection in the shoot and were of the opinion that these changes are the key factors behind tip necrosis and symptom recovery. They also observed that glyceraldehyde 3-phosphate dehydrogenase failed to express in tissues around the meristem, and absence of this factor is the principal cause of meristem exclusion as this protein is required for virus replication and growth (Medzihradszky et al., 2019). Thus, there are manifold pathways for viral exclusion and entry in SAM.

In a recent experiment, the stem cell regulator WUSCHEL protein has been shown as a protector of SAM–stem cells against invasion of CMV. WUSCHEL is induced in response to CMV infection, and it depresses viral accumulation in the meristem central and peripheral zones. WUSHEL does not allow viral protein synthesis as it represses synthesis of *S*-adenosyl-L-methionine(Sam)–dependent methyltransferases. SAM-methyltransferases are required for-RNA processing and ribosome stability. This finding also establishes WUSCHEL-mediated broad-spectrum innate antiviral immunity in plants (Wu et al., 2020).

### Natural Pathways of Virus Resistance

The invading viruses are never welcome in plants, and using several types of antiviral silencing pathways, plants defend themselves. These pathways are interconnected and RNA silencing factors along with associated processes dominate the silencing pathways and also influence other pathways (Wang T. et al., 2018). Knowledge of these natural pathways is important to engineer virus resistance. In these pathways, miRNAs and virus-responsive miRNAs are also intricately involved (Wang et al., 2016; Wang K. et al., 2018). Here, to keep things a bit simpler, we avoid discussing the roles played by miRNAs. Primary VsiRNAs, which are triggered as a result of replication of RNA viruses or convergent transcription of DNA viruses such as geminiviruses, may not play big roles in RNA silencing-mediated antiviral silencing. The RDR-deficient (especially RDR1 and RDR6) plants exhibit enhanced viral toxicity in *A. thaliana*, tobacco, tomato, pepper, rice, and maize, and conversely, the RDR-overexpressing plants of tomato, tobacco, and pepper well-protect themselves against viruses such as TMV, SHMV, TCV, CMV, etc. Thus, RNA silencing-mediated immunity depends on presence of amplified siRNAs or secondary VsiRNAs. It has shown that RDR1 and RDR6 divide their jobs in producing secondary VsiRNAs from distinct regions of the viral genomic components to protect the WT plants from viral attack (Wang et al., 2010). In rice, RDR1 orthologs are generally kept repressed by dimeric forms of MADS-box proteins, namely, OsMADS23, OsMADS27a, and OsMADS57. Following viral infections by say, RSV, miR444 is overexpressed, and this miRNA targets the aforementioned proteins for down-regulation, thus inducing RDR1 following RSV infection. The overexpression of miR444 in rice causes overexpression of RDR1, which in turn resists the invading RSV. Thus, RDR1 seems to play a major antiviral silencing role in most of the plant kingdom (Wang et al., 2016). The DCL4-generated 21-nt VsiRNAs are responsible for intracellular PTGS of viral RNAs, whereas the DCL2-produced 22-nt VsiRNAs are majorly active for intercellular silencing of viral RNAs (Qin et al., 2017; Zhang et al., 2019). Plants also harbor many AGO proteins; for example, *Arabidopsis* possesses 10 AGO proteins. However, not all of them are involved in antiviral silencing. Only AGO1 and AGO2 have been found to be important for antiviral silencing against plant RNA viruses (Carbonell and Carrington, 2015). Besides the regular RNA silencing factors, other host factors responsible for antiviral silencing, such as ALA1/ALA2/AVi-2, etc., are also known (Carbonell and Carrington, 2015).

#### Effective VsiRNAs

VsiRNAs are generated from all over the viral genome, although there could be some hot spots depending on the host–viral species pair concerned. However, all the VsiRNAs cannot be incorporated in RISC and even among the incorporated ones; only a few are active in slicing viral RNAs. Thus. only a few VsiRNAs are competent for PTGS. Thus, from the pool of VsiRNAs, the effective VsiRNAs (eVsiRNAs) need to be identified for efficient antiviral silencing. Gago-Zachert et al. (2019) successfully devised an *in vitro* TBY2-based system to identify the eVsiRNAs using the pathogen TBSV as the model virus.

The TBY2 extract contained all the Dicers, AGO, and other RNA-silencing factors to enable identification of eVsiRNAs. The *in vitro* extract can dice the dsRNA of TBSV in three siRNA forms, but 24-nt siRNAs were the predominant species. The siRNAs were allowed to be incorporated in the flag-tagged AGO1 or AGO2 in presence of the TBY2 extract. The AGOs were *in vitro* translated and supplemented in the RISC-incorporation assay. The incorporated VsiRNAs were then detected from immune-precipitated RISC by NGS technique. A substantial portion of diced VsiRNAs was not found in RISC. Some of these RISC-incorporated siRNAs were finally selected, and their synthetic forms were programmed with RISC in a slicing assay prepared with the TBY2 extract, and again, only a few of the selected ones were competent for slicing. The slicing-competent siRNAs were the eVsiRNA as these can be used efficiently for resistance against TBSV (Zachert et al., 2019). Hence, for engineering virus resistance, identification of eVsiRNA sequences is very important, which could be found only after several filtration steps as mentioned previously with an *in vitro* system. Other VsiRNA sequences are ineffective to confer virus resistance.

#### VsiRNAs for Avoidance

A few VsiRNAs are also known to affect host gene expression, causing disease symptoms in hosts. Such VsiRNAs can be predicted by bioinformatics means and should be deselected for consideration of engineering virus resistance. A 369-nt-long ssRNA genome of Y-satellite is mostly associated with the CMV virus. Hence, following infection, VsiRNAs are also generated from this satellite RNA. One such 22-bp-long VsiRNA of Y-satellite has a complementary match with the chlorophyll biosynthetic gene, CHLI, of many tobacco species. As a result, the CHL1-mRNA gets sliced following CMV infection, and this loss of CHL1 results in yellowing symptoms of infected tobacco (Smith et al., 2011). In another event, VsiRNAs derived from cotton infected with a begomovirus, cotton leaf curl Multan virus (CLCuMuV), which is associated with a betasatellite called cotton leaf curl Multan betasatellite (CLCuMuB), were examined. Many host transcripts were found to be down-regulated following slicing by a few VsiRNAs (Wang et al., 2016). Similarly, the VsiRNAs from maize infected with sugarcane mosaic virus were also examined, and approximately 42 maize transcripts were possibly cleaved by VsiRNAs. These maize transcripts are involved in chloroplast functions, as well as biotic and abiotic stresses (Xia et al., 2018). As these VsiRNAs are detrimental for host functions, these should be eliminated while choosing viral sequences for engineering virus resistance.

#### Virus-Activated Host siRNAs for Antiviral Silencing

When CMV with its 2b gene inactivated (CMVΔ2b) infects *Arabidopsis*, a set of endogenous (originating from more than a thousand of genes) siRNAs come up that are highly dependent on host RDR1 protein for biogenesis. But these expressed siRNAs vanish altogether in presence of CMV-2b RNA-silencing suppressor. These mostly 21-nt siRNAs are called vasiRNAs, which are more like VsiRNAs and are produced predominantly by DCL4. For their biogenesis, they do not require SGS3 and RDR6, and these efficiently load on AGO2 complexes, whereas a small fraction also load on AGO1 complexes. The vasiRNAs target the expression of the host genes from where these are derived, and such down-regulation leads to antiviral silencing. The overall function of these vasiRNAs is to broaden the scope antiviral silencing. Various strains of CMV, which are deficient in 2b protein, are also competent to produce vasiRNAs. TuMV also generates vasiRNAs in *Arabidopsis* with similar genetic requirements such as CMV, but the viral RNA suppressor, i.e., HC-Pro, does not have any effect on the production of vasiRNAs. The extent of accumulation of vasiRNAs depends on the siRNA degrading factors such as exoribonuclease 4/ethylene-insensitive 5, and the loss of these factors enhances the level of vasiRNAs. Such enhancement also results in higher virus resistance without having any effect on VsiRNAs (Cao et al., 2014). A host factor, namely, AVi2, has been screened, and it contributes to the biogenesis of vasiRNAs (Guo et al., 2018). In rice, RDR1 is generally repressed by MADS-box proteins, which bind to CArG containing promoter of RDR1, and these repressors are also targets of miR444. Following infection by RSV, miR444 is induced, releasing the repression of RDR1 and such induction results in generation of vasiRNAs in rice (Wang et al., 2016).

#### Transcriptional Gene Silencing of DNA Viruses

The plant viruses belonging to families *Geminiviridae*, *Nanoviridae*, and *Caulimoviridae* harbor DNA genomes. Of these, TGS of geminiviruses has been worked upon to some details, whereas the same for other family members are underreported. So, we would focus here on geminiviruses only. The single-stranded circular DNA of geminiviruses enter plant nuclei following infection that gets quickly converted to dsDNA forms known as replicative forms (RFs). DNA replication and transcription initiate with the RF templates. However, the RF templates do not remain in the naked form but complex with histones to form nucleosome-like structures. They look like a ring of 13–14 beads in electron microscopy, and biochemical assays reveal that the IR region of viral DNA and promoters of early transcripts such as Rep, AC3 etc., are somewhat free of histones (Raja et al., 2010). These structures may be called viral chromatins, and similar to plant chromatins, they also undergo modifications. The DNA and the histones of viral chromatins are modified in a manner similar to host chromatins (Raja et al., 2010; Rosa et al., 2018). Following geminivirus infections in susceptible plants, VsiRNAs of all three sizes (21-, 22-, 24-mers) are generated by cognate dicers (Aregger et al., 2012). Within the infected tissues, the abundance of the 24-nt VsiRNAs is the highest. The 21-/22-mer siRNAs are involved in PTGS of viral transcripts, whereas the 24-nt ones are used to methylate the viral DNA at the cytosine bases in RdDM pathway (Raja et al., 2010; Rosa et al., 2018). The DNA methylation at the IR region is very high, and open promoters of viral DNA are also relatively well-methylated. Such methylation causes reduction in biogenesis of viral transcripts and consequently falls in viral DNA titer. Such methylated DNA thus undergoes TGS. The TGS of viral chromatin is also accompanied by histone methylation such as histone H3K9 dimethylation, etc. (Raja et al., 2010; Rosa et al., 2018). It is worthwhile to note that the 24-nt VsiRNA biogenesis and amplification are carried out by RDR2 enzymes, and thus, TGS is also influenced by secondary siRNAs. TGS of viral chromatin is looked upon as a defense reaction of host against the viral pathogen and also causes phenomena such as recovery of emerging leaves from the viral diseases. Such recovery has been evidenced with pepper golden mosaic virus (PepGMV) infecting pepper plants, L2 mutant of beet curly top virus (BCTV) infecting *N. benthamiana* or *Arabidopsis*, and many other host–virus combinations (Rodríguez-Negrete et al., 2009; Raja et al., 2010). The L2 mutant is required for TGS as the L2 protein opposes methylation of viral DNA.

Most of the RdDM mutants relax TGS to some extent but do not abolish it totally, reflecting redundancy in the pathway. As a consequence, RdDM plant mutants are also more sensitive to viruses compared to their WT versions. For example, the methylation pathway mutants of *Arabidopsis*, including nrpd2a (deficient for both POL IV and POL V), ago4, and ddm1, show hypersensitivity to CaLCuV and BCTV. Similar increased sensitivity is observed in mutant plants lacking the non-CG methyltransferases drm1/drm2 and cmt3 and the H3K9 methyltransferase kyp2/suvh4. Hyper susceptibility is also evidenced in adenosine kinase mutants (adk1 and adk2), which are interrupted in methyl cycle, providing Sam which is the key cofactor for methyltransferase. As complete loss of Met proteins results in growth defects of host plants, experiments with met1 mutants (CG maintenance methyltransferase) are difficult to carry out. However, met1 heterozygotes exhibit normal growth but show moderately enhanced susceptibility to the geminiviruses (Raja et al., 2010; Ramirez-Prado et al., 2018). *Arabidopsis* mutants, deficient in DNA methylation, also show reduced H3K9me2 levels in the viral genome and consequent hyper-susceptibility to geminiviruses. However, it should be remembered that DNA methylation per se is not sufficient for recruitment of DNA methyltransferases (Ramirez-Prado et al., 2018).

As viruses have evolved to encode PTGS-inhibitors, they also code for TGS-inhibitory proteins. The AL2/L2 proteins that inhibit methylation of viral chromatin by blocking ADK-pathway can efficiently block TGS (Raja et al., 2010). Many other viral inhibitors of TGS have been described, such as the geminivirus Rep that down-regulates the expression of DNA methyltransferases, decreasing DNA methylation levels in the viral genome and hence TGS. The other inhibitors include tomato leaf curl Yunnan virus (TLCYNV) C4, MYMIV- AC5, tomato yellow leaf curl Sardinia virus (TYLCSV) Rep, TYLCV- V2 and cotton leaf curl Kokhran virus (CLCKV) Rep, TrAP and beta-C1 and CLCuMuV- V2 proteins, etc. Most of these proteins interact with the various components of RdDM pathways to inhibit the biochemical function of the RdDM proteins. For example, CLCuMuV-V2 protein interacts with *N. benthamiana–*AGO4 and suppresses RdDM; tomato yellow leaf curl China virus (TYLCCNV) beta-C1 interacts with *S*-adenosyl homocysteine hydrolase and blocks its activity *in vitro* (Yang et al., 2011; Wang et al., 2019). Thus, these viral inhibitors act as functional RdDM mutants.

## Genetic Engineering for Virus Resistance With Secondary siRNAs

### PDR Approach

In the PDR approach, a viral gene can be used as a transgene, and the transgenic plants are supposed to be resistant to the virus from which the transgene is borrowed. In the initial days of PDR-mediated resistance, the overexpressed protein of the viral gene has been held as the key factor for virus resistance. But later, it has been found that an untranslatable form of the same gene sequence can also confer resistance, and the transgenics that produce the least amount of transgene RNA/protein are the best for providing most robust resistance. Thus, it was established that proteins are not the important factors, but the sense RNA/siRNA/secondary siRNAs of the transgene are the key components of resistance (Rosa et al., 2018).

There are some commercial products out in the market based on this principle, for example, the Hawaiian PRSV-resistance papaya, transgenic squash, potato resistant to viruses released in the United States; transgenic pepper, tomatoes with resistance to CMV released in China (in mid-1990s); and transgenic plum with resistance to PPV released in the United States (in mid 2000s) (Rosa et al., 2018).

### Hairpin-RNA Constructs

Peter Waterhouse’s group showed that better virus resistance can be achieved when both sense RNA and antisense RNA of a viral gene are simultaneously expressed in plant cell compared to the case of expressing either sense or antisense viral RNA (Waterhouse et al., 1998). The dsRNA of the viral sequence leads to more efficient virus resistance. The same group later showed that the transgenic plants with intron splicable hairpin RNA constructs with viral gene or gene fragments are most efficient (100%) in offering the strongest resistance to RNA and DNA viruses (Smith et al., 2000). In the majority of the cases, the viral RNA-silencing suppressor gene(s) has been used as a silencing target to generate virus resistance (Gaffar and Koch, 2019). In Brazil, transgenic bean with resistance to a begomovirus, bean golden mosaic virus (BGMV), is being commercially cultivated (Rosa et al., 2018). Here the hairpin RNA of BGMV-Rep gene was used as a transgene. Tomato plants have been engineered to express hairpin transcripts corresponding to a 728-nt fragment of the TYLCV- *Rep* coding sequence. The transgenic plants overproducing 21- and 22-nt siRNAs have been found almost immune to TYLCV challenge (Fuentes et al., 2016). Cassava mosaic disease (CMD), caused by a begomovirus, is a severe constraint to cassava production. A transgene-derived RNA hairpin, homologous to an overlapping region of the South African cassava mosaic virus (SACMV)-Rep and the VSR proteins (AC1/AC4), has been found to confer tolerance in the CMD-susceptible model cassava cultivar 60444 (Walsh et al., 2019). All the hp-RNA constructs gave rise to secondary siRNAs following viral infection in the transgenics. Details of hp-RNA constructs for use in various crops and plants to generate virus resistance have been reviewed in many places (Rosa et al., 2018; Gaffar and Koch, 2019). Transitive vectors that produce secondary RNAs only have also been used for virus resistance but they have been mostly found to be a bit inferior to hp-RNA constructs.

### a-miRNA Constructs

Vectors using sense or antisense PDR constructs, hp-RNA constructs, VIGS, etc., suffer from defects such as “off-target” effects on the host chromosome that are generated by a multitude of siRNAs appearing from the vectors. To circumvent this, transgenes of artificial miRNA (amiR) have been used successfully. We have shown that tomato transgenics overproducing the amiRNAs to silence the conserved regions of ToLCV- AV2/AC2 genes can very well tolerate various leaf curl viruses of tomato (Vu et al., 2013). Earlier we mentioned effective VsiRNAs, which are very efficient for silencing viral genomes. These e-VsiRNAs can be expressed by amiR approaches for generating robust virus resistance. A list of plants/crops made resistant against the viruses using a-miR technology can be found in many reviews (Cisneros and Carbonell, 2020).

Recently, two web tools representing the systematic and high-throughput method for the simple and fast-forward design, generation and functional analysis of large numbers of artificial miRNA (amiRNAs) constructs has been described. These are WMD3 and P-SAMS, which are optimized for both effectiveness and specificity of designed amiRNAs. The web-predicted amiRNAs are transiently expressed individually in several *N. benthamiana* plants, which are subsequently inoculated with the virus of interest. The antiviral activity of each amiRNA construct is monitored by inspecting appearance of viral symptoms and carrying out molecular analyses of virus titer accumulation in infected tissues. This methodology has been used to identify highly effective amiRNAs against a viroid PSTVd and the virus tomato spotted wilt virus (TSWV) in *N. benthamiana*. A detailed account of various amiR constructs to confer virus resistance in plants has been given in many recent reviews (Rosa et al., 2018; Gaffar and Koch, 2019; Cisneros and Carbonell, 2020).

### PhasiRNA Constructs

Two types of silencing tools have been used in literature that generates Syn-tasiRNAs/PhasiRNAs to silence genes of interest. These tools rely solely on the action of secondary siRNAs and have been used extensively for both basic studies of gene function and improving agronomic traits of crops. Here we would focus only on antiviral activities of these tools.

#### atasiRNA/Syn-tasiRNA

Artificial or synthetic siRNAs (atasiRNA or syn-tasiRNA) are expressed in plants from engineered TAS sequences of *Arabidopsis*. A single or few tasiRNA sequences of precursor TAS DNA are removed and substituted by sequences of interest of equivalent sizes in the same position in the engineered TAS DNA. The engineered TAS DNA acts as a transgene and, when introduced in plants, gives rise to syn-tasiRNA primary transcript (pri-syn-tasiRNA), which eventually spawns phased syn-tasiRNA duplexes of 21 base pairs in the usual tasiRNA biogenesis pathway. When TAS1 precursor is used, miR173/AGO1 complex acts an initiating slicer. Similarly, miR390/AGO7 is the slicer when the precursor is TAS3a RNA. In all cases, the guide strands of syn-tasiRNAs are incorporated into AGO1/RISC, which functions as a site-specific endonuclease to degrade multiple sites of one or multiple viral RNAs as guided by syn-tasiRNA sequences. As such silencing processes of target genes are induced by synthetic tasiRNAs, these are also called SIGS. An account of these target genes can be found in several reviews (Carbonell, 2019). However, we would like to focus here on viral target genes only.

Chen et al. (2016) engineered the *Arabidopsis* TAS3a gene, which allowed expression of three syn-tasiRNAs to target CMV and other three syn-tasiRNAs to target TuMV simultaneously. The sequences of syn-tasiRNAs were designed following the complementary sequences of target viral RNA and by using WMD3-Web miRNA designer to have minimal off-target effects on host plants. Transgenic *A. thaliana* plants overexpressing these six syn-siRNAs showed high level of resistance to single as well as both viruses. Such encouraging results established for the first time that syn-tasiRNAs could be used for broad applications of multiple virus resistance (Chen et al., 2016).

Most of the contributions on antiviral strategies using SIGS have come from the lab of Carbonell et al. (2019a, b), Cisneros and Carbonell (2020), Lopez-Dolz et al. (2020). Carbonell’s group has worked extensively on a deadly RNA virus, namely, TSWV. TSWV belongs to the genus *Tospovirus*, which cause plant tissue necrosis post infection. Tospoviruses rank among the 10 most detrimental plant viruses worldwide, and their recent resurgence and spread into novel hosts have sparked major concerns among agriculturalists and horticulturists. TSWV is a tripartite RNA virus consisting of three genomic RNA components, namely, L (large), M (medium), and S (small) RNAs. The entire genome codes for six proteins via five different ORFs. The L component is completely antisense and encodes the RdRP; the M segment is ambisense and encodes the NSm MP and the structural Gn/Gc proteins required for vector transmission; the S component is ambisense and encodes the nucleocapsid N protein and the silencing suppressor NSs.

**FIGURE 1:**
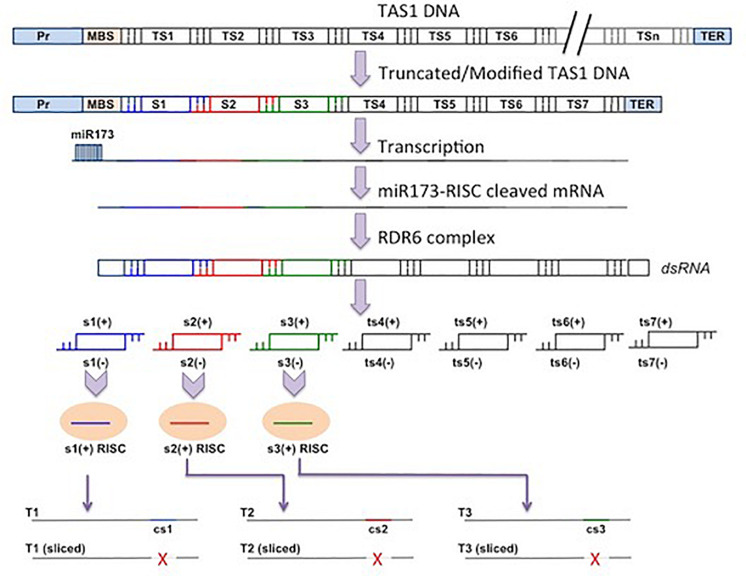
Schematics of artificial or synthetic tasiRNA (s) induced silencing (SIGS) using TAS1 DNA as vector backbone. ‘TS’ represents DNA encoding tasiRNA and ‘S’ represents DNA encoding synthetic tasiRNA, which replaces the ‘TS’ DNA. The sequence of ‘S’ is designed to slice (X) the T-mRNA. Following cleavage of mRNA, as transcribed by the engineered TAS1 DNA, by miR173-RISC, the 3′ fragment of the cleaved mRNA is converted to dsRNA by RDR6-SGS3 complex. DCL4 dicing releases the synthetic (s) tasiRNAs which eventually slice the T-mRNA. The other abbreviation used are Pr: promoter; MBS: miR173 binding site; TER: Transcription termination sequence; + and −: both strands of the tasiRNA; T: mRNA target of the synthetic tasiRNA (s); cs: complementary sequence. Synthetic tasiRNA (s) are shown in different.

For SIGS, the TAS precursors are the natural choices as the vector backbone, and these precursors possess an in-built multiplexing capability, which allows incorporation of multiple syn-tasiRNAs in a single construct (Figure 1). This characteristic combined with the availability of high-throughput syn-tasiRNA “B/c” vectors makes room for the generation of robust antiviral syn-tasiRNA constructs. Such strategy has resulted in high levels of resistance against TSWV in *N. benthamiana* and *S. lycopersicum* plants, respectively. As the atasiRNAs/syn-tasiRNAs are computationally designed with user-friendly web tools such as P-SAMS (see text footnote 1) (Fahlgren et al., 2016), the induced silencing is highly specific and devoid of the off-target effects that are usually associated with other RNAi approaches. The P-SAMS designs the synthetic sRNAs that contain (i) 5′ U for incorporation in an AGO1-RISC and (ii) a C in position 19 to generate a star strand which cannot be loaded in AGO1-RISC, thus avoiding competition for AGO1 loading.

As there are many TSWV isolates that escape the spell of resistance gene like SW5, Carbonell’s group came up with development of broad-range anti-TSWV strategies employing the SIGS technique (Carbonell et al., 2019b). They used six variants of TSWV that are spread around the world and using the P-SAMS–related analyses on these TSWV RNA sequences’ they predicted the optimal, relatively conserved and off-target free (in *N. benthamiana*) a-miR sequences for silencing the various viral genes. Five a-miRs from each of the three genomic components were selected that matched with their TSs nearly perfectly and were distributed throughout the genome. All of the L-amiRs targeted RdRP mRNA, three and two M-amiRs targeted NSm viral RNA and mRNA respectively, and one and four S-amiRs targeted N-mRNA and NSs viral RNA, respectively. These sequences were then subjected to high-throughput functional analyses for their anti-TSWV activities in agroinfiltration-based transient assays in *N. benthamiana*. Of all these 15 amiRs, only two from L segment (amiR-TSWV-L3/L5) and other two from the M component (amiR-TSWV-M1/M3) were efficient in controlling TSWV local and systemic lesions in *N. benthamiana*. These four sequences were used to construct the SIGS vector using the TAS1c DNA backbone, and the synthetic tasiRNAs were expressed in *N. benthamiana* transiently using the 35S promoter. For expression of syn-tasiRNAs, coinfiltration with miR173 was necessary as the tobacco plant do not encode the miR173. A highly anti-TSWV response was observed with the SIGS vector. Finally, the investigators examined the comparative silencing activities of amiRs and syn-tasiRNAs and concluded that the syn-tasiRNAs were much better silencing tools than the a-miRs.

Earlier the efficacies of SIGS as antiviral strategies were established in model plants such as *Arabidopsis* and tobacco, but Carbonell’s group established the same for the first time in a crop plant, namely, tomato, the natural host of TSWV. The aforementioned four amiRs and the syn-tasiRNAs were transgenically expressed in tomato independently. For syn-tasiRNA expression, the transgene also included the miR-173 expression cassette as the tomato plant does not encode this miRNA. Several transgenic lines with variable levels of expression of syn-tasiRNAs were obtained. When these lines were examined for anti-TSWV activities, it was found that low expressing two lines failed to show resistance, but in all others (10) transgenics expressing suitable amount of syn-tasiRNAs were highly resistant against challenge TSWV. In contrast, the majority of the amiRNA lines were susceptible, and only a couple of lines with higher amiRNA accumulation were resistant. A systematic analysis of the a-miR TSs in the progeny viruses from infected plant leaves revealed the emergence of TS mutations exclusively in susceptible amiRNA lines. The investigators concluded that subinhibitory amiRNA accumulation led to the emergence of TS mutations in replicating TSWV, whereas simultaneous multiplexed targeting of viral RNAs with several syn-tasiRNAs restricted the capability of escape mutations, thus establishing robust plant resistance (Carbonell et al., 2019a).

Controlling the degree of induced gene silencing to fine-tune it, keeping the promoter of the expression of silencing sRNAs invariant, is a very desirable objective but not so easy to achieve. Carbonell’s group has revealed the strategies to achieve the same by playing with the order of positioning (with respect to the cleavage point of the initiator miRNA) of the expressed syn-tasiRNAs and tweaking the sequences of the same (Lopez-Dolz et al., 2020). The investigators have shown that the level and activity of *Arabidopsis* TAS1c-based syn-tasiRNAs gradually reduce as the syn-tasiRNA is expressed from positions more distant to the trigger miR173 cleavage site. And second, syn-tasiRNA activity can also be altered by changing the amount of base-pairing between the 3′ end of the syn-tasiRNA and the 5′ end of the target RNA. Both strategies were used to finely modulate the degree of silencing of TSWV genes in *N. benthamiana* in transient assays. The target viral gene silencing was highest when the syn-tasiRNA was expressed from the position nearest to the initiator mRNA cleavage site and the degree of silencing diminished as the same syn-tasiRNA was expressed far from the aforementioned cleavage site. Moreover, zero to six mutations were introduced at the 3′ end of syn-tasiRNAs, which gradually reduce the degree of base-pairing between the syn-tasiRNA and its target gene. Gradual reduction in base-pairing caused gradual reduction in cognate viral gene silencing.

#### MiRNA-Induced Gene Silencing

The PHAS loci biosynthesize phasiRNAs with the initial trigger of 22-nt miRNAs (in a few cases with 21-nt miRNA). Besides the *Arabidopsis* miRNA 173 (for TAS1) and miR390 (for TAS3), there is a host of miRNAs that are used for fine-tuning the regulation of plant development and immunity genes. A number of 22-nt miRNAs, such as miR482/2118, miR1507, miR2109, miR5300, and miR6019, trigger biosynthesis of phasiRNAs from the nucleotide-binding leucine-rich repeat (NB-LRR) gene family containing hundreds of members in eudicots that constitute the majority of plant disease resistance (R) genes. These phasiRNAs suppress the expression of these resistance genes to avoid toll on plant growth in absence of pathogen invasion (Fei et al., 2018). *Arabidopsis* loss-of-function mutants in NB-LRR genes (triggered by mir472, a variant of the miR482 family found in *Arabidopsis*) or phasiRNA generation [with mutation in RDR6 (rdr6) genes] exhibit enhanced effector-triggered immunity–based resistance to some pathogens (Boccara et al., 2014). The miR1510 (22nt) in mungbean is responsible for phasiRNAs synthesis for suppression NBS-LRR genes. However, in soybean, miR1510 is processed as 21nt entity but quickly matures as 22-nt form by mono-uridylation, and this matured form is responsible for suppressing NB-LRR genes in soybean (Fei et al., 2018). Similarly, miR535, miR828, miR858, and miRNA N1 also control disease resistance genes in *Litchi* (Ma et al., 2018).

**FIGURE 2:**
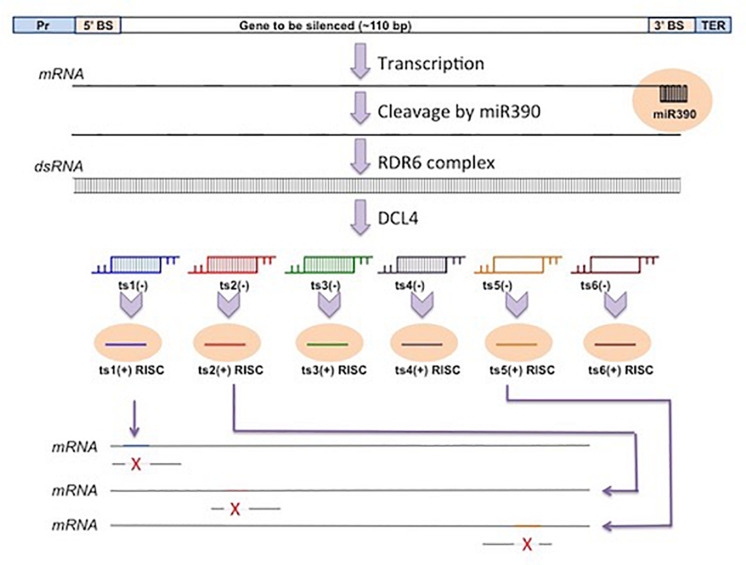
Schematics of miRNA induced silencing (MIGS) using miR390 as the initiator. A fragment of DNA is completely substituted by a hypothetical gene of 130 bp, which is flanked by 5′ binding site (5′BS) and 3′ binding site (3′BS) of the miRNA. Following cleavage by miR390-RISC, the cleaved mRNA is converted to dsRNA by RDR6 complex. This is diced by DCL4 to spawn the tasiRNAs (ts1 to ts6). The tasiRNAs slice the target mRNA (T) at appropriate site (shown as X). The other abbreviation used are Pr: promoter; TER: Transcription termination sequence; + and −: both strands of the tasiRNA; cs: complementary sequence.

These phasiRNAs generating miRNAs can also be put to biotechnological use. Felippes et al. (2012) showed that any *Arabidopsis* gene fused with miR173 binding site at the 5′ end of the gene is capable of producing phasiRNAs/tasiRNAs. In this way, he developed loss of function mutants of AG, ELF3, FT and LFY genes of *Arabidopsis* with clear phenotypes. As miR173 is specific for *Arabidopsis*, they showed that similar approach could be adopted for creation of loss of function mutants if miR173 pre-miRNA could be co-expressed in other plants such as *N. benthamiana* (Felippes et al., 2012). This approach is termed as MIGS (Figure 2) and has been used in engineering plant immunity against viruses. An account of the genes targeted by MIGS technology can be found in some reviews (Carbonell, 2019). In this approach, most of the TAS DNA vector or part of the vector can be removed and substituted by the gene or gene fragment of interest. The tasiRNAs spawned by MIGs are generally fraught with danger of showing off-target effects as they do not undergo selection processes, unlike the tasiRNAs produced by SIGS vectors. The syn-tasiRNAs are carefully designed by web tools and are validated by functional means; thus, these are mostly free of off-target effects unlike the tasiRNAs generated by MIGS that do not undergo any purifying selection.

In an experiment conducted by Zhao et al. (2015b), the TAS1c locus has been engineered by removing six native siRNAs and substituting with a 126-bp fragment of CP gene of PPV. The predicted 21-nt phasiRNAs in register with miR173-guided cleavage point appeared in transient agroinfiltration assays in *N. benthamiana*. The agroinfiltrated leaves were challenged 3 days later by mechanical inoculation with purified virions of a recombinant PPV expressing GFP. An average of 2.4 infection foci per leaf were observed on the plants expressing only miR173, but plants expressing tasiRNA-CP showed high resistance to PPV. However, when other constructs were used for tasiRNA generation, they found appearance of siRNA not only because of involvement of tasiRNA pathway but also because of PTGS of the recombinant construct (Zhao et al., 2015b).

We also made use of this approach to confer resistance to a begomovirus, namely, ToLCNDV. The viral RNA-silencing suppressor genes (either AC2 or AC4) were sandwiched between 5′ and 3′ binding sites of miR390, a 21-nt miRNA which is mostly conserved in plant species and triggers tasiRNAs from TAS3 locus. In this construct, the whole of TAS3 DNA was removed and replaced by VSR AC2/AC4. When this construct was agroinfiltrated in tobacco or tomato, phasiRNAs of 21-nt size were generated in the infiltrated leaves. A central mutation spanning 4 nt in the 3′ binding element abolished the generation of siRNAs, establishing thereby that the siRNAs did not result from PTGS of the introduced construct. To check functionality of the tasiRNAs, the ones derived from the AC2 gene were allowed to act on viral transcripts that were made from introduction of the infectious viral clone in plants. The infectious viral clone was introduced on leaves that were at the upper side of tasiRNA generating leaf. The cleavage points on the viral transcripts were mapped by degradome analysis and were found to be localized only in the AC2 transcripts and nowhere else in the entire viral transcripts. This observation showed that the tasiRNAs are functional and mobile as well. We then carried out transient agroinfiltration assays to check resistance to ToLCNDV, and the vast majority of the plants showed resistance against the virus at the systemic leaves. Subsequently, we generated stable transgenics with these constructs in both tobacco and tomato. Approximately 95% of the plants showed features of tolerant plants against ToLCNDV and similar viruses. The virus resistance was monitored by inspecting reduction of leaf curl and stunting characteristics of the transgenics, as well as molecular analyses of viral DNA formation in the systemic leaves of T0 and T1 transgenic plants. A few of the transgenic lines were almost immune to the invading ToLCNDVs at the T0 and T1 stage (Singh et al., 2015, 2019).

## Conclusion and Perspectives

RNA silencing in plants involves the movement of amplified 24-, 21-, 22-nt sRNAs for SR spread and SS, resulting in PTGS or TGS. The SR silencing spreads from cell-to-cell through the symplastic movement, whereas SS follows a phloem-based translocation pathway from (metabolic) source. In the last few years, we have witnessed substantial progress in interpreting the mechanisms of RNA-silencing spread. Such mechanisms are utilized to amplify siRNAs or transitive RNAs that have great roles to play in biotechnological work. Any gene of unknown sequence can be silenced if transitive RNA is allowed within the transcript of target gene. The secondary siRNAs can be used to silence any unrelated gene having no homology with the silencer siRNAs. Many *Arabidopsis* endo- genes have been silenced using this approach. The atasiRNA/MIGS vectors produce siRNAs based on biogenesis of secondary siRNAs that can be utilized in plant development, flower control, and plant defense against viruses. Abiotic stress-resistant plants can also be developed using such vectors.

Although the importance of secondary siRNAs for plant growth and defense has been appreciated in literature in recent years, there are lot of undiscovered, dark matters associated with their biogeneses and functions. In some organisms such as *C. elegans*, secondary siRNAs are chemically different from their primary counterparts. The 5′ ends of secondary siRNAs have di/triphosphates, whereas primary siRNAs have monophosphates. So, we need to know if plant secondary siRNAs are chemically different from the primary ones. Careful chromatographic analyses coupled with the biochemical treatments of the pool of siRNAs can resolve this question. Primary siRNAs are protected from nucleolytic degradation by addition of sugar moieties by Hen1 proteins. Are the secondary siRNAs treated similarly by Hen1 enzymes? Even if they are, do they retain the sugar moieties during their intercellular trafficking? How do the secondary siRNAs move out of the incipient cells and move in distant recipient cells? Are there channel proteins that help them in such trafficking? Do these siRNAs acquire novel chemical tags during their journey across the cells as there could be kinases and phosphates and other nucleic acid–modifying enzymes residing on the cellular membranes? In the intracellular silencing, DCL4 plays the major role, but for intercellular silencing, DCL4 is inhibitory, and DCL2 plays the major role. So the question remains: How such distinctive regulation occurs, and what are the regulatory factors? It is often said that processes of intracellular silencing are well worked out, but it is not clear if similar mechanisms are at work for intercellular silencing. Does the intercellular silencing require different participatory factors? We anticipate that these unresolved questions will be uncovered soon in the future.

Besides these dark matters, the siRNA-based technologies face challenges of different kinds. Some siRNAs turn out to be ineffective to cause silencing as the target site (TS) might have cryptic secondary structures, or the site could be inaccessible due to preoccupancy by *in vivo* factors, which are difficult to predict by bioinformatics means. It has been established that not all VsiRNAs can cause silencing of viral genes. Not all VsiRNAs are incorporated in RISC complexes, and not even all of those that are incorporated in RISC can cause silencing. Hence, it is important to find the effective e-VsiRNAs that are surely capable of causing silencing. Fortunately, the procedures prescribed by Zachert et al. (2019), as mentioned earlier within the text, pave the path to identify the eVsiRNAs. It is possible to expect development of machine learning system for prediction of eVsiRNAs from the compilations of already available eVsiRNAs in the future. Second, the TS of viral genes often gets mutated, and thus, the siRNAs targeting those sites become invalid to cause silencing. Hence, the conserved viral sites need to be targeted, and often two to three siRNAs that are locationally distributed need to be applied to silence a particular viral gene (Carbonell, 2019). Third and above all, most of the dangers are derived from the so-called “off-target” effects. The limited homology between the siRNA and non-targeted genes can cause silencing of non-targeted genes. The results of experiments involving multiple siRNAs thus become unreliable as the non-intended silencing makes the outcome of targeted silencing a noisy and dubious one (Jackson and Linsley, 2004). Excessive siRNAs may also act as miRNAs and suppress translation without affecting transcript levels (Saxena et al., 2003). These off-target effects are also sources of potential biosafety issues for the RNAi-transgenic plants that are meant for crop improvement. Such off-target effects can be minimized by proper usage of improved bioinformatics, which can predict siRNAs with relatively fewer off-target effects. Proper usages of web tools like P-SAMS, WMD-3 (mentioned within text), siRNA scan^2^, etc., can screen for desirable and effective siRNAs. Use of non-constitutive promoters (inducible, tissue-specific, etc.) to express siRNAs can also cut down the quantum of off-target effects. The sequences of syn-tasiRNAs are derived from appropriate bioinformatics followed by experimental validation, and thus, these are relatively free of off-target effects. Moreover, these syn-tasiRNAs have the advantages of regulatable silencing, which is often required (Lopez-Dolz et al., 2020). Thus, it seems that the SIGS technology might find greater biotechnological applications in the future. Despite the obvious challenges, siRNA-based technologies (knockdown) are preferred over others like CRISPR-Cas or other site-specific knockout methods as the formers are easy to handle and cost-effective and are applicable in cases where knockout techniques fail.

## Author Contributions

NS-M and SM prepared the initial draft. All authors participated in extensive editing of the manuscript and read and approved the write-up.

## Conflict of Interest

The authors declare that the research was conducted in the absence of any commercial or financial relationships that could be construed as a potential conflict of interest.

## Footnotes

1 http://p-sams.carringtonlab.org/

2 http://bioinfo2.noble.org/RNAiScan.htm

## References

[B1] Agrawal N. Dasaradhi P. V. Mohmmed A. Malhotra P. Bhatnagar R. K. Mukherjee S. K. (2003). RNA interference: biology, mechanism, and applications. *Microbiol. Mol. Biol. Rev.* 67 657–685. 10.1128/mmbr.67.4.657-685.2003 14665679 PMC309050

[B2] Agüero J. Vives M. Velázquez K. Pina J. A. Navarro L. Moreno P. (2014). Effectiveness of gene silencing induced by viral vectors based on Citrus leaf blotch virus is different in *Nicotiana benthamiana* and citrus plants. *Virology* 460-461 154–164. 10.1016/j.virol.2014.04.017 25010281

[B3] Alder M. N. Dames S. Gaudet J. Mango S. E. (2003). Gene silencing in *Caenorhabditis elegans* by transitive RNA interference. *RNA* 9 25–32. 10.1261/rna.2650903 12554873 PMC1370367

[B4] Allen E. Xie Z. Gustafson A. M. Carrington J. C. (2005). microRNA-directed phasing during trans-acting siRNA biogenesis in plants. *Cell* 121 207–221. 10.1016/j.cell.2005.04.004 15851028

[B5] Aregger M. Borah B. K. Seguin J. Rajeswaran R. Gubaeva E. G. Zvereva A. S. (2012). Primary and secondary siRNAs in geminivirus-induced gene silencing. *PLoS Pathog.* 8:e1002941. 10.1371/journal.ppat.1002941 23028332 PMC3460622

[B6] Axtell M. J. (2013). Classification and comparison of small RNAs from plants. *Annu. Rev. Plant Biol.* 64 137–159. 10.1146/annurev-arplant-050312-120043 23330790

[B7] Axtell M. J. Jan C. Rajagopalan R. Bartel D. P. (2006). A two-hit trigger for siRNA biogenesis in plants. *Cell* 127 565–577. 10.1016/j.cell.2006.09.032 17081978

[B8] Baeg K. Iwakawa H. O. Tomari Y. (2017). The poly(A) tail blocks RDR6 from converting self mRNAs into substrates for gene silencing. *Nat. Plants* 3:17036. 10.1038/nplants.2017.36 28319057

[B9] Bai S. Kasai A. Yamada K. Li T. Harada T. (2011). A mobile signal transported over a long distance induces systemic transcriptional gene silencing in a grafted partner. *J. Exp. Bot.* 62 4561–4570. 10.1093/jxb/err163 21652532 PMC3170550

[B10] Baum J. A. Bogaert T. Clinton W. Heck G. R. Feldmann P. Ilagan O. (2007). Control of coleopteran insect pests through RNA interference. *Nat. Biotechnol.* 25 1322–1326. 10.1038/nbt1359 17982443

[B11] Benkovics A. H. Timmermans M. C. (2014). Developmental patterning by gradients of mobile small RNAs. *Curr. Opin. Genet. Dev.* 27 83–91. 24929831 10.1016/j.gde.2014.04.004

[B12] Bhogale S. Mahajan A. S. Natarajan B. Rajabhoj M. Thulasiram H. V. Banerjee A. K. (2013). MicroRNA156: a potential graft-transmissible microRNA that modulates plant architecture and tuberization in *Solanum tuberosum* ssp.andigena. *Plant Physiol.* 164 1011–1027. 10.1104/pp.113.230714 24351688 PMC3912076

[B13] Bleys A. Vermeersch L. Van Houdt H. Depicker A. (2006). The frequency and efficiency of endogene suppression by transitive silencing signals is influenced by the length of sequence homology. *Plant Physiol.* 142 788–796. 10.1104/pp.106.083956 16891552 PMC1586036

[B14] Boccara M. Sarazin A. Thiébeauld O. Jay F. Voinnet O. Navarro L. (2014). The *Arabidopsis* miR472-RDR6 silencing pathway modulates PAMP- and effector-triggered immunity through the post-transcriptional control of disease resistance genes. *PLoS Pathog.* 10:e1003883. 10.1371/journal.Ppat.100388 PMC3894208 24453975

[B15] Boerjan W. Bauw G. Montagu M. V. Inze D. (1994). Distinct phenotypes generated by overexpression and suppression of S-Adenosyl-L-Methionine synthetase reveal developmental patterns of gene silencing in tobacco. *Plant Cell* 6 1401–1414. 10.2307/3869977 7994174 PMC160529

[B16] Bond D. M. Baulcombe D. C. (2015). Epigenetic transitions leading to heritable, RNA-mediated de novo silencing in *Arabidopsis thaliana*. *Proc. Natl. Acad. Sci. U.S.A.* 112 917–922. 10.1073/pnas.1413053112 25561534 PMC4311854

[B17] Borsani O. Zhu J. Verslues P. E. Sunkar R. Zhu J. K. (2005). Endogenous siRNAs derived from a pair of natural cis-antisense transcripts regulate salt tolerance in *Arabidopsis*. *Cell* 123 1279–1291. 10.1016/j.cell.2005.11.035 16377568 PMC3137516

[B18] Bouché N. Lauressergues D. Gasciolli V. Vaucheret H. (2006). An antagonistic function for *Arabidopsis* DCL2 in development and a new function for DCL4 in generating viral siRNAs. *Embo J.* 25 3347–3356. 10.1038/sj.emboj.7601217 16810317 PMC1523179

[B19] Brilli M. Asquini E. Moser M. Bianchedi P. L. Perazzolli M. Si-Ammour A. (2018). A multi-omics study of the grapevine-downy mildew (*Plasmopara viticola*) pathosystem unveils a complex protein coding- and noncoding-based arms race during infection. *Sci. Rep.* 8:757. 10.1038/s41598-018-19158-8 29335535 PMC5768699

[B20] Brosnan C. A. Mitter N. Christie M. Smith N. A. Waterhouse P. M. Carroll B. J. (2007). Nuclear gene silencing directs reception of long-distance mRNA silencing in *Arabidopsis*. *Proc. Natl. Acad. Sci. U.S.A.* 104 14741–14746. 10.1073/pnas.0706701104 17785412 PMC1964546

[B21] Brosnan C. A. Voinnet O. (2011). Cell-to-cell and long-distance siRNA movement in plants: mechanisms and biological implications. *Curr. Opin. Plant Biol.* 14 580–587. 10.1016/j.Pbi.2011.07.011 21862389

[B22] Buhtz A. Pieritz J. Springer F. Kehr J. (2010). Phloem small RNAs, nutrient stress responses, and systemic mobility. *BMC Plant Biol.* 10:64. 10.1186/1471-2229-10-64 20388194 PMC2923538

[B23] Buhtz A. Springer F. Chappell L. Baulcombe D. C. Kehr J. (2008). Identification and characterization of small RNAs from the phloem of *Brassica napus*. *Plant J.* 53 739–749. 10.1111/j.1365-313x.2007.03368.x 18005229

[B24] Buscaill P. Sanguankiattichai N. Lee Y. J. Kourelis J. Preston G. Van Der Hoorn R. A. L. (2020). Agromonas: a rapid disease assay for *Pseudomonas syringae* growth in agroinfiltrated leaves. *BioRxiv* 10.1101/2020.08.10.243808 PMC7898395 33124734

[B25] Butterbach P. Verlaan M. G. Dullemans A. Lohuis D. Visser R. G. Bai Y. (2014). Tomato yellow leaf curl virus resistance by Ty-1 involves increased cytosine methylation of viral genomes and is compromised by cucumber mosaic virus infection. *Proc. Natl. Acad. Sci. U.S.A.* 111 12942–12947. 25136118 10.1073/pnas.1400894111 PMC4156758

[B26] Cai Q. Qiao L. Wang M. He B. Lin F. M. Palmquist J. (2018). Plants send small RNAs in extracellular vesicles to fungal pathogen to silence virulence genes. *Science* 360 1126–1129. 10.1126/science.aar4142 29773668 PMC6442475

[B27] Calarco J. P. Borges F. Donoghue M. T. Van Ex F. Jullien P. E. Lopes T. (2012). Reprogramming of DNA methylation in pollen guides epigenetic inheritance via small RNA. *Cell* 151 194–205. 10.1016/j.cell.2012.09.001 23000270 PMC3697483

[B28] Cao M. Du P. Wang X. Yu Y. Q. Qiu Y. H. Li W. (2014). Virus infection triggers widespread silencing of host genes by a distinct class of endogenous siRNAs in *Arabidopsis*. *Proc. Natl. Acad. Sci. U.S.A.* 111 14613–14618. 10.1073/pnas.1407131111 25201959 PMC4209997

[B29] Carbonell A. (2019). Secondary small interfering RNA-based silencing tools in plants: an update. *Front. Plant Sci.* 10:687. 10.3389/fpls.2019.00687 31191587 PMC6547011

[B30] Carbonell A. Carrington J. C. (2015). Antiviral roles of plant ARGONAUTES. *Curr. Opin. Plant Biol.* 27 111–117. 26190744 10.1016/j.pbi.2015.06.013 PMC4618181

[B31] Carbonell A. Lisón P. Daròs J. A. (2019a). Multi-targeting of viral RNAs with synthetic trans-acting small interfering RNAs enhances plant antiviral resistance. *Plant J.* 100 720–737. 10.1111/tpj.14466 31350772 PMC6899541

[B32] Carbonell A. López C. Daròs J. A. (2019b). Fast-forward identification of highly effective artificial small RNAs against different Tomato spotted wilt virus isolates. *Mol. Plant Microbe Interact.* 32 142–156. 10.1094/MPMI-05-18-0117-TA 30070616

[B33] Carlsbecker A. Lee J.-Y. Roberts C. J. Dettmer J. Lehesranta S. Zhou J. (2010). Cell signalling by microRNA165/6 directs gene dose-dependent root cell fate. *Nature* 465 316–321. 10.1038/nature08977 20410882 PMC2967782

[B34] Castel S. E. Martienssen R. A. (2013). RNA interference in the nucleus: roles for small RNAs in transcription, epigenetics and beyond. *Nat. Rev. Genet.* 14 100–112. 10.1038/nrg3355 23329111 PMC4205957

[B35] Catalanotto C. Pallotta M. Refalo P. Sachs M. S. Vayssie L. Macino G. (2004). Redundancy of the two dicer genes in transgene-induced posttranscriptional gene silencing in *Neurospora crassa*. *Mol. Cell. Biol.* 24 2536–2545. 10.1128/mcb.24.6.2536-2545.2004 14993290 PMC355837

[B36] Chan S. W. Henderson I. R. Jacobsen S. E. (2005). Gardening the genome: DNA methylation in *Arabidopsis thaliana*. *Nat. Rev.* 6 351–360. 10.1038/nrg1601 15861207

[B37] Chen H. M. Chen L.-T. Patel K. Li Y. H. Baulcombe D. C. Wu S. H. (2010). 22-nucleotide RNAs trigger secondary siRNA biogenesis in plants. *Proc. Natl. Acad. Sci. U.S.A.* 107 15269–15274. 10.1073/pnas.1001738107 20643946 PMC2930544

[B38] Chen D. Meng Y. Ma X. Mao C. Bai Y. Cao J. (2010). Small RNAs in angiosperms: sequence characteristics, distribution and generation. *Bioinformatics* 26 1391–1394. 10.1093/bioinformatics/btq150 20378553

[B39] Chen L. Cheng X. Cai J. Zhan L. Wu X. Liu Q. (2016). Multiple virus resistance using artificial trans-acting siRNAs. *J. Virol. Methods* 228 16–20. 26562057 10.1016/j.jviromet.2015.11.004

[B40] Chitwood D. H. Nogueira F. T. Howell M. D. Montgomery T. A. Carrington J. C. Timmermans M. C. (2009). Pattern formation via small RNA mobility. *Genes Dev.* 23 549–554. 10.1101/gad.1770009 19270155 PMC2658522

[B41] Choudhary S. Thakur S. Bhardwaj P. (2019). Molecular basis of transitivity in plant RNA silencing. *Mol. Biol. Rep.* 46 4645–4660. 10.1007/s11033-019-04866-9 31098805

[B42] Christie M. Croft L. J. Carroll B. J. (2011). Intron splicing suppresses RNA silencing in *Arabidopsis*. *Plant J.* 68 159–167. 10.1111/j.1365-313X.2011.04676.x 21689169

[B43] Cisneros A. E. Carbonell A. (2020). Artificial small RNA-based silencing tools for antiviral resistance in plants. *Plants* 9:669. 10.3390/plants9060669 32466363 PMC7356032

[B44] Cogoni C. Macino G. (1999). Gene silencing in *Neurospora crassa* requires a protein homologous to RNA-dependent RNA polymerase. *Nature* 399 166–169. 10.1038/20215 10335848

[B45] Creasey K. M. Zhai J. Borges F. Van Ex F. Regulski M. Meyers B. C. (2014). miRNAs trigger widespread epigenetically activated siRNAs from transposons in *Arabidopsis*. *Nature* 508 411–415. 10.1038/nature13069 24670663 PMC4074602

[B46] Csorba T. Kontra L. Burgyán J. (2015). Viral silencing suppressors: tools forged to fine-tune host-pathogen coexistence. *Virology* 479-480 85–103. 25766638 10.1016/j.virol.2015.02.028

[B47] Cuperus J. T. Carbonell A. Fahlgren N. Garcia-Ruiz H. Burke R. T. Takeda A. (2010). Unique functionality of 22-nt miRNAs in triggering RDR6-dependent siRNA biogenesis from target transcripts in *Arabidopsis*. *Nat. Struct. Mol. Biol.* 17 997–1003. 10.1038/nsmb.1866 20562854 PMC2916640

[B48] Dalakouras A. Wassenegger M. Dadami E. Ganopoulos I. Pappas M. L. Papadopoulou K. (2019). Genetically modified organism-free RNA interference: exogenous application of RNA molecules in plants. *Plant Physiol.* 182 38–50. 10.1104/pp.19.00570 31285292 PMC6945881

[B49] Dalmay T. Hamilton A. Rudd S. Angell S. Baulcombe D. C. (2000). An RNA-dependent RNA polymerase gene in *Arabidopsis* is required for posttranscriptional gene silencing mediated by a transgene but not by a virus. *Cell* 101 543–553. 10.1016/s0092-8674(00)80864-8 10850496

[B50] Daxinger L. Kanno T. Bucher E. Winden J. V. D. Naumann U. Matzke A. J. M. (2008). A stepwise pathway for biogenesis of 24-nt secondary siRNAs and spreading of DNA methylation. *Embo J.* 28 48–57. 10.1038/emboj.2008.260 19078964 PMC2633084

[B51] de la Luz Gutiérrez-Nava M. Aukerman M. J. Sakai H. Tingey S. V. Williams R. W. (2008). Artificial trans-acting siRNAs confer consistent and effective gene silencing. *Plant Physiol.* 147 543–551. 10.1104/pp.108.118307 18441221 PMC2409013

[B52] Deng P. Muhammad S. Cao M. Wu L. (2018). Biogenesis and regulatory hierarchy of phased small interfering RNAs in plants. *Plant Biotechnol. J.* 16 965–975. 10.1111/pbi.12882 29327403 PMC5902766

[B53] Dougherty W. G. Lindbo J. A. Smith H. A. Parks T. D. Swaney S. Proebsting W. M. (1994). RNA-mediated virus resistance in transgenic plants: exploitation of a cellular pathway possibly involved in RNA degradation. *Mol. Plant Microbe Interact.* 7 544–552. 7949323

[B54] Dubrovina A. S. Kiselev K. V. (2019). Exogenous RNAs for gene regulation and plant resistance. *Int. J. Mol. Sci.* 20 2282. 10.3390/ijms20092282 31072065 PMC6539981

[B55] Dunoyer P. Brosnan C. A. Schott G. Wang Y. Jay F. Alioua A. (2010a). An endogenous, systemic RNA silencing pathway in plants. *Embo J.* 29 1699–1712. 10.1038/emboj.2010.65 20414198 PMC2876969

[B56] Dunoyer P. Schott G. Himber C. Meyer D. Takeda A. Carrington J. C. (2010b). Small RNA duplexes function as mobile silencing signals between plant cells. *Science* 328 912–916. 10.1126/science.1185880 20413458

[B57] Dunoyer P. Himber C. Ruiz-Ferrer V. Alioua A. Voinnet O. (2007). Intra- and intercellular RNA interference in *Arabidopsis thaliana* requires components of the microRNA and heterochromatic silencing pathways. *Nat. Genet.* 39 848–856. 10.1038/ng2081 17558406

[B58] Eamens A. Wang M. B. Smith N. A. Waterhouse P. M. (2008). RNA silencing in plants: yesterday, today, and tomorrow. *Plant Physiol.* 147 456–468. 10.1104/pp.108.117275 18524877 PMC2409047

[B59] Fahlgren N. Hill S. T. Carrington J. C. Carbonell A. (2016). P-SAMS: a web site for plant artificial microRNA and synthetic trans-acting small interfering RNA design. *Bioinformatics* 32 157–158. 10.1093/bioinformatics/btv534 26382195 PMC4681993

[B60] Fahlgren N. Montgomery T. A. Howell M. D. Allen E. Dvorak S. K. Alexander A. L. (2006). Regulation of AUXIN RESPONSE FACTOR3 by TAS3 tasiRNAaffects developmental timing and patterning in arabidopsis. *Curr. Biol.* 16 939–944. 10.1016/j.cub.2006.03.065 16682356

[B61] Fei Q. Yu Y. Liu L. Zhang Y. Baldrich P. Dai Q. (2018). Biogenesis of a 22-nt microRNA in *Phaseoleae* species by precursor-programmed uridylation. *Proc. Natl. Acad. Sci. U.S.A.* 115 8037–8042. 10.1073/pnas.1807403115 30012624 PMC6077734

[B62] Felippes F. F. Wang J. W. Weigel D. (2012). MIGS: miRNA-induced gene silencing. *Plant J.* 70 541–547. 22211571 10.1111/j.1365-313X.2011.04896.x

[B63] Felippes F. F. D. Ott F. Weigel D. (2010). Comparative analysis of non-autonomous effects of tasiRNAs and miRNAs in *Arabidopsis thaliana*. *Nucleic Acids Res.* 39 2880–2889. 10.1093/nar/gkq1240 21134910 PMC3074149

[B64] Feng L. Xia R. Liu Y. (2019). Comprehensive characterization of miRNA and PHAS loci in the diploid strawberry (*Fragaria vesca*) genome. *Hortic. Plant J.* 5 255–267. 10.1016/j.hpj.2019.11.004

[B65] Filichkin S. A. DiFazio S. P. Brunner A. M. Davis J. M. Yang J. K. Kalluri U. C. (2007). Efficiency of gene silencing in *Arabidopsis* : direct inverted repeats vs. transitive RNA silencing vectors. *Plant Biotechnol. J.* 5 615–626. 10.1111/j.1467-7652.2007.00267.x 17573806

[B66] Fuentes A. Carlos N. Ruiz Y. Callard D. Sánchez Y. Ochagavía M. E. (2016). Field trial and molecular characterization of RNA silencing-transgenic tomato plants that exhibit resistance to Tomato Yellow Leaf Curl Geminivirus. *Mol. Plant Microbe Interact.* 29 197–209. 10.1094/MPMI-08-15-0181-R 26713353

[B67] Gaffar F. Y. Koch A. (2019). Catch me if you can! RNA silencing-based improvement of antiviral plant immunity. *Viruses* 11:673. 10.3390/v11070673 31340474 PMC6669615

[B68] Gago-Zachert S. Schuck J. Weinholdt C. Knoblich M. Pantaleo V. Grosse I. (2019). Highly efficacious antiviral protection of plants by small interfering RNAs identified *in vitro*. *Nucleic Acids Res.* 47 9343–9357. 10.1093/nar/gkz678 31433052 PMC6755098

[B69] Garcia-Ruiz H. Takeda A. Chapman E. J. Sullivan C. M. Fahlgren N. Brempelis K. J. (2010). Arabidopsis RNA-Dependent RNA Polymerases and Dicer-Like Proteins in antiviral defense and small interfering RNA biogenesis during Turnip mosaic virus infection. *Plant Cell* 22 481–496. 10.1105/tpc.109.073056 20190077 PMC2845422

[B70] Gasciolli V. Mallory A. C. Bartel D. P. Vaucheret H. (2005). Partially redundant functions of arabidopsis DICER-like enzymes and a role for DCL4 in producing trans-acting siRNAs. *Curr. Biol.* 15 1494–1500. 10.1016/j.cub.2005.07.024 16040244

[B71] Ghag S. B. (2017). Host induced gene silencing, an emerging science to engineer crop resistance against harmful plant pathogens. *Physiol. Mol. Plant Pathol.* 100 242–254. 10.1016/j.Pmpp.2017.10.003

[B72] Ghoshal B. Sanfaçon H. (2015). Symptom recovery in virus-infected plants: revisiting the role of RNA silencing mechanisms. *Virology* 47 167–179. 10.1016/j.virol.2015.01.008 25677651

[B73] Guan X. Pang M. Nah G. Shi X. Ye W. Stelly D. M. (2014). miR828 and miR858 regulate homoeologous MYB2 gene functions in *Arabidopsis trichome* and cotton fibre development. *Nat. Commun.* 5:3050. 10.1038/ncomms4050 24430011

[B74] Guo Q. Liu Q. Smith N. A. Liang G. Wang M. B. (2016). RNA silencing in plants: mechanisms, technologies and applications in horticultural crops. *Curr. Genomics* 17 476–489. 10.2174/1389202917666160520103117 28217004 PMC5108043

[B75] Guo Z. Wang X. B. Wang Y. Li W. X. Gal-On A. Ding S. W. (2018). Identification of a new host factor required for antiviral rna silencing and amplification of viral siRNAs. *Plant Physiol.* 176 1587–1597. 10.1104/pp.17.01370 29184028 PMC5813567

[B76] Hamilton A. Voinnet O. Chappell L. Baulcombe D. (2002). Two classes of short interfering RNA in RNA silencing. *Embo J.* 21 4671–4679. 10.1093/emboj/cdf464 12198169 PMC125409

[B77] Hamilton A. J. Brown S. Yuanhai H. Ishizuka M. Lowe A. Solis A. G. A. (1998). A transgene with repeated DNA causes high frequency, post-transcriptional suppression of ACC-oxidase gene expression in tomato. *Plant J.* 15 737–746. 10.1046/j.1365-313x.1998.00251.x 29368810

[B78] Han Y. Grierson D. (2002). Relationship between small antisense RNAs and aberrant RNAs associated with sense transgene mediated gene silencing in tomato. *Plant J.* 29 509–519. 10.1046/j.1365-313x.2002.01236.x 11846883

[B79] Han Y. Zhang B. Qin X. Li M. Guo Y. (2015). Investigation of a miRNA-induced gene silencing technique in petunia reveals alterations in miR173 precursor processing and the accumulation of secondary siRNAs from endogenous genes. *PLoS One* 10:e0144909. 10.1371/journal.Pone.0144909 26658695 PMC4701714

[B80] Härtl K. Kalinowski G. Hoffmann T. Preuss A. Schwab W. (2017). RNA silencing-mediated endogene silencing in strawberry fruit: detection of primary and secondary siRNAs by deep sequencing. *Plant Biotechnol. J.* 15 658–668. 10.1111/pbi.12664 27862816 PMC5398998

[B81] Havecker E. R. Wallbridge L. M. Hardcastle T. J. Bush M. S. Kelly K. A. Dunn R. M. (2010). The arabidopsis RNA-directed DNA methylation argonautes functionally diverge based on their expression and interaction with target loci. *Plant Cell* 22 321–334. 10.1105/tpc.109.072199 20173091 PMC2845420

[B82] Henderson I. R. Zhang X. Lu C. Johnson L. Meyers B. C. Green P. J. (2006). Dissecting *Arabidopsis thaliana* DICER function in small RNA processing, gene silencing and DNA methylation patterning. *Nat. Genet.* 38 721–725. 10.1038/ng1804 16699516

[B83] Himber C. Dunoyer P. Moissiard G. Ritzenthaler C. Voinnet O. (2003). Transitivity-dependent and -independent cell-to-cell movement of RNA silencing. *Embo J.* 22 4523–4533. 10.1093/emboj/cdg431 12941703 PMC202373

[B84] Hinas A. Wright A. J. Hunter C. P. (2012). SID-5 Is an endosome-associated protein required for efficient systemic rna silencing in *C. elegans*. *Curr. Biol.* 22 1938–1943. 10.1016/j.cub.2012.08.020 22981770 PMC10518204

[B85] Hou Y. Zhai Y. Feng L. Karimi H. Z. Rutter B. D. Zeng L. (2019). A phytophthora effector suppresses trans-kingdom RNA silencing to promote disease susceptibility. *Cell Host Microb.* 25 153.e5–165.e5. 10.1016/j.chom.2018.11.007 PMC9208300 30595554

[B86] Huang G. Allen R. Davis E. L. Baum T. J. Hussey R. S. (2006). Engineering broad root-knot resistance in transgenic plants by RNA silencing silencing of a conserved and essential root-knot nematode parasitism gene. *Proc. Natl. Acad. Sci. U.S.A.* 103 14302–14306. 10.1073/pnas.0604698103 16985000 PMC1570184

[B87] Hudzik C. Hou Y. Ma W. Axtell M. J. (2020). Exchange of small regulatory RNAs between plants and their pests. *Plant Physiol.* 182 51–62. 10.1104/pp.19.00931 31636103 PMC6945882

[B88] Hunter C. Winston W. Molodowitch C. Feinberg E. Shih J. Sutherlin M. (2006). Systemic RNA silencing in *Caenorhabditis elegans*. *Cold Spring Harb. Symp. Quant. Biol.* 71 95–100. 10.1101/sqb.2006.71.060 17381285

[B89] Imin N. Mohd-Radzman N. A. Ogilvie H. A. Djordjevic M. A. (2013). The peptide-encoding CEP1 gene modulates lateral root and nodule numbers in *Medicago truncatula*. *J. Exp. Bot.* 64 5395–5409. 10.1093/jxb/ert369 24259455

[B90] Jackson A. L. Linsley P. S. (2004). Noise amidst the silence: off-target effects of siRNAs? *Trends Genet.* 20 521–524. 15475108 10.1016/j.tig.2004.08.006

[B91] Jacobs T. B. Lawler N. J. LaFayette P. R. Vodkin L. O. Parrott W. A. (2016). Simple gene silencing using the trans-acting siRNA pathway. *Plant Biotechnol. J.* 14 117–127. 10.1111/pbi.12362 25816689 PMC11389014

[B92] Jahan S. N. Åsman A. K. Corcoran P. Fogelqvist J. Vetukuri R. R. Dixelius C. (2015). Plant-mediated gene silencing restricts growth of the potato late blight pathogen *Phytophthora infestans*. *J. Exp. Bot.* 66 2785–2794. 25788734 10.1093/jxb/erv094 PMC4986879

[B93] Johnson C. Kasprzewska A. Tennessen K. Fernandes J. Nan G. L. Walbot V. (2009). Clusters and superclusters of phased small RNAs in the developing inflorescence of rice. *Genome Res.* 19 1429–1440. 10.1101/gr.089854.108 19584097 PMC2720183

[B94] Kalantidis K. Tsagris M. Tabler M. (2006). Spontaneous short-range silencing of a GFP transgene in *Nicotiana benthamianais* possibly mediated by small quantities of siRNA that do not trigger systemic silencing. *Plant J.* 45 1006–1016. 10.1111/j.1365-313x.2006.02664.x 16507090

[B95] Kanazawa A. Inaba J. I. Shimura H. Otagaki S. Tsukahara S. Matsuzawa A. (2010). Virus-mediated efficient induction of epigenetic modifications of endogenous genes with phenotypic changes in plants. *Plant J.* 65 156–168. 10.1111/j.1365-313x.2010.04401.x 21175898

[B96] Kasai A. Kanehira A. Harada T. (2010). miR172 can move long distances in *Nicotiana benthamiana*. *Open Plant Sci. J.* 4 1–7. 10.2174/1874294701004010001

[B97] Katiyar-Agarwal S. Morgan R. Dahlbeck D. Borsani O. Villegas A. Jr. Zhu J. K. (2006). A pathogen-inducible endogenous siRNA in plant immunity. *Proc. Natl. Acad. Sci. U.S.A.* 103 18002–18007. 10.1073/pnas.0608258103 17071740 PMC1693862

[B98] Kehr J. Buhtz A. (2007). Long distance transport and movement of RNA through the phloem. *J. Exp. Bot.* 59 85–92. 10.1093/jxb/erm176 17905731

[B99] Komiya R. (2017). Biogenesis of diverse plant phasiRNAs involves an miRNA-trigger and Dicer-processing. *J. Plant Res.* 130 17–23. 10.1007/s10265-016-0878-0 27900550 PMC5219027

[B100] Kørner C. J. Pitzalis N. Peña E. J. Erhardt M. Vazquez F. Heinlein M. (2018). Crosstalk between PTGS and TGS pathways in natural antiviral immunity and disease recovery. *Nat. Plants* 4 157–164. 10.1038/s41477-018-0117-x 29497161

[B101] Kumar V. Mishra S. K. Rahman J. Taneja J. Sundaresan G. Mishra N. S. (2015). Mungbean yellow mosaic Indian virus encoded AC2 protein suppresses RNA silencing by inhibiting *Arabidopsis* RDR6 and AGO1 activities. *Virology* 486 158–172. 10.1016/j.virol.2015.08.015 26433748

[B102] Kume K. Tsutsumi K. Saitoh Y. (2010). TAS1 trans-acting siRNA targets are differentially regulated at low temperature, and TAS1 trans-acting siRNA mediates temperature-controlled At1g51670 expression. *Biosci. Biotechnol. Biochem.* 74 1435–1440. 10.1271/bbb.100111 20622450

[B103] Kurihara Y. Watanabe Y. (2004). From the cover: *Arabidopsis* micro-RNA biogenesis through Dicer-like 1 protein functions. *Proc. Natl. Acad. Sci. U.S.A.* 101 12753–12758. 10.1073/pnas.0403115101 15314213 PMC515125

[B104] Lee W. S. Fu S. F. Li Z. Murphy A. M. Dobson E. A. Garland L. (2016). Salicylic acid treatment and expression of an RNA-dependent RNA polymerase 1 transgene inhibit lethal symptoms and meristem invasion during tobacco mosaic virus infection in *Nicotiana benthamiana*. *BMC Plant Biol.* 16:15. 10.1186/s12870-016-0705-8 26757721 PMC4710973

[B105] Lewsey M. G. Hardcastle T. J. Melnyk C. W. Molnar A. Valli A. Urich M. A. (2016). Mobile small RNAs regulate genome-wide DNA methylation. *Proc. Natl. Acad. Sci. U.S.A.* 113 E801–E810. 10.1073/pnas.1515072113 26787884 PMC4760824

[B106] Li S. Liu J. Liu Z. Li X. Wu F. He Y. (2014). Heat-induced *tas1* target1 mediates thermotolerance via heat stress transcription factor A1A-directed pathways in arabidopsis. *Plant Cell* 26 1764–1780. 10.1105/tpc.114.124883 24728648 PMC4036584

[B107] Liang D. White R. G. Waterhouse P. M. (2012). Gene silencing in arabidopsis spreads from the root to the shoot, through a gating barrier, by template-dependent, nonvascular, cell-to-cell movement. *Plant Physiol.* 159 984–1000. 10.1104/pp.112.197129 22582134 PMC3387722

[B108] Liang D. White R. G. Waterhouse P. M. (2014). Mobile gene silencing in Arabidopsis is regulated by hydrogen peroxide. *PeerJ* 2:e701. 10.7717/peerj.701 25551023 PMC4277490

[B109] Lin S. I. Chiang S. F. Lin W. Y. Chen J. W. Tseng C. Y. Wu P. C. (2008). Regulatory network of microRNA399 and PHO2 by systemic signaling. *Plant Physiol.* 147 732–746. 10.1104/pp.108.116269 18390805 PMC2409027

[B110] Lindbo J. A. Silva-Rosales L. Proebsting W. M. Dougherty W. G. (1993). Induction of a highly specific antiviral state in transgenic plants: implications for regulation of gene expression and virus resistance. *Plant Cell* 5 1749–1759. 10.1105/tpc.5.12.1749 12271055 PMC160401

[B111] Lipardi C. Wei Q. Paterson B. M. (2001). RNA silencing as random degradative PCR: siRNA primers convert mRNA into dsRNAs that are degraded to generate new siRNAs. *Cell* 107 297–307. 10.1016/s0092-8674(01)00537-2 11701121

[B112] Lisch D. (2009). Epigenetic regulation of transposable elements in plants. *Annu. Rev. Plant Biol.* 60 43–66. 10.1146/annurev.arplant.59.032607.092744 19007329

[B113] Liu J. He Y. Amasino R. Chen X. (2004). siRNAs targeting an intronic transposon in the regulation of natural flowering behavior in *Arabidopsis*. *Genes Dev.* 18 2873–2878. 10.1101/gad.1217304 15545622 PMC534648

[B114] Liu Y. Ke L. Wu G. Xu Y. Wu X. Xia R. (2017). miR3954 is a trigger of phasiRNAs that affects flowering time in citrus. *Plant J.* 92 263–275. 10.1111/tpj.13650 28749585

[B115] Lopez-Dolz L. Spada M. Daros J.-A. Carbonell A. (2020). Fine-tune control of targeted RNAi efficacy by plant artificial small RNAs. *Nucleic Acid Res.* 48 6234–6250. 32396204 10.1093/nar/gkaa343 PMC7293048

[B116] Lucas W. J. Ham B.-K. Kim J. Y. (2009). Plasmodesmata – bridging the gap between neighboring plant cells. *Trends Cell Biol.* 19 495–503. 10.1016/j.tcb.2009.07.003 19748270

[B117] Ma W. Chen C. Liu Y. Zeng M. Meyers B. C. Li J. (2018). Coupling of microRNA-directed phased small interfering RNA generation from long noncoding genes with alternative splicing and alternative polyadenylation in small RNA-mediated gene silencing. *New Phytol.* 217 1535–1550. 10.1111/nph.14934 29218722

[B118] Makeyev E. V. Bamford D. H. (2002). Cellular RNA-dependent RNA polymerase involved in posttranscriptional gene silencing has two distinct activity modes. *Mol. Cell* 10 1417–1427. 10.1016/s1097-2765(02)00780-3 12504016

[B119] Mao Y. B. Cai W. J. Wang J. W. Hong G. J. Tao X. Y. Wang L. J. (2007). Silencing a cotton bollworm P450 monooxygenase gene by plant-mediated RNA silencing impairs larval tolerance of gossypol. *Nat. Biotechnol.* 25 1307–1313. 10.1038/nbt1352 17982444

[B120] Marin E. Jouannet V. Herz A. Lokerse A. S. Weijers D. Vaucheret H. (2010). miR390, *Arabidopsis* TAS3 tasiRNAs, and their AUXIN RESPONSE FACTOR targets define an autoregulatory network quantitatively regulating lateral root growth. *Plant Cell* 22 1104–1117. 10.1105/tpc.109.072553 20363771 PMC2879756

[B121] Martens H. Novotny J. Oberstrass J. Steck T. L. Postlethwait P. Nellen W. (2002). RNA silencing in Dictyostelium: the role of RNA-directed RNA polymerases and double-stranded RNase. *Mol. Biol. Cell* 13 445–453. 10.1091/mbc.01-04-0211 11854403 PMC65640

[B122] Martínez, de Alba A. E. Elvira-Matelot E. Vaucheret H. (2013). Gene silencing in plants: a diversity of pathways. *Biochim. Biophys. Acta* 1829 1300–1308. 24185199 10.1016/j.bbagrm.2013.10.005

[B123] Martín-Hernández A. M. Baulcombe D. C. (2008). Tobacco rattle virus 16-kilodalton protein encodes a suppressor of RNA silencing that allows transient viral entry in meristems. *J. Virol.* 82 4064–4071. 10.1128/JVI.02438-07 18272576 PMC2292987

[B124] Masson I. L. Jauvion V. Bouteiller N. Rivard M. Elmayan T. Vaucheret H. (2012). Mutations in the *Arabidopsis* H3K4me2/3 demethylase JMJ14 suppress posttranscriptional gene silencing by decreasing transgene transcription. *Plant Cell* 24 3603–3612. 10.1105/tpc.112.103119 23001035 PMC3480290

[B125] Matzke M. A. Mosher R. A. (2014). RNA-directed DNA methylation: an epigenetic pathway of increasing complexity. *Nat. Rev. Genet.* 15 394–408. 10.1038/nrg3683 24805120

[B126] Maule A. J. Benitez-Alfonso Y. Faulkner C. (2011). Plasmodesmata – membrane tunnels with attitude. *Curr. Opin. Plant Biol.* 14 683–690. 10.1016/j.Pbi.2011.07.007 21820942

[B127] McKinney H. H. (1929). Mosaic diseases in Canary Islands west africa and Gibraltar. *J. Agric. Res.* 39 557–578.

[B128] Medzihradszky A. Gyula P. Sós-Hegedûs A. Szittya G. Burgyán J. (2019). Transcriptome reprogramming in the shoot apical meristem of CymRSV-infected *Nicotiana benthamiana* plants associates with viral exclusion and the lack of recovery. *Mol. Plant Pathol.* 20 1748–1758. 10.1111/mpp.12875 31560831 PMC6859499

[B129] Melnyk C. W. Molnar A. Baulcombe D. C. (2011a). Intercellular and systemic movement of RNA silencing signals. *Embo J.* 30 3553–3563. 10.1038/emboj.2011.274 21878996 PMC3181474

[B130] Melnyk C. W. Molnar A. Bassett A. Baulcombe D. C. (2011b). Mobile 24-nt small RNAs direct transcriptional gene silencing in the root meristems of *Arabidopsis thaliana*. *Curr. Biol.* 21 1678–1683. 10.1016/j.cub.2011.08.065 21962713

[B131] Mermigka G. Verret F. Kalantidis K. (2015). RNA silencing movement in plants. *J. Integ. Plant Biol.* 58 328–342. 10.1111/jipb.12423 26297506

[B132] Mlotshwa S. Pruss G. J. Peragine A. Endres M. W. Li J. Chen X. (2008). DICER-LIKE2 plays a primary role in transitive silencing of transgenes in arabidopsis. *PLoS One* 3:e1755. 10.1371/journal.Pone.0001755 18335032 PMC2262140

[B133] Moissiard G. Parizotto E. A. Himber C. Voinnet O. (2007). Transitivity in *Arabidopsis* can be primed, requires the redundant action of the antiviral Dicer-like 4 and Dicer-like 2, and is compromised by viral-encoded suppressor proteins. *RNA* 13 1268–1278. 17592042 10.1261/rna.541307 PMC1924903

[B134] Molnar A. Melnyk C. W. Bassett A. Hardcastle T. J. Dunn R. Baulcombe D. C. (2010). Small silencing RNAs in plants are mobile and direct epigenetic modification in recipient cells. *Science* 328 872–875. 10.1126/science.1187959 20413459

[B135] Montgomery T. A. Howell M. D. Cuperus J. T. Li D. Hansen J. E. Alexander A. L. (2008a). Specificity of ARGONAUTE7-miR390 interaction and dual functionality in TAS3 trans-acting siRNA formation. *Cell* 133 128–141. 10.1016/j.cell.2008.02.033 18342362

[B136] Montgomery T. A. Yoo S. J. Fahlgren N. Gilbert S. D. Howell M. D. Sullivan C. M. (2008b). AGO1-miR173 complex initiates phased siRNA formation in plants. *Proc. Natl. Acad. Sci. U.S.A.* 105 20055–20062. 10.1073/pnas.0810241105 19066226 PMC2598728

[B137] Mosher R. A. Schwach F. Studholme D. Baulcombe D. C. (2008). Pol IVb influences RNA-directed DNA methylation independently of its role in siRNA biogenesis. *Proc. Natl. Acad. Sci. U.S.A.* 105 3145–3150. 10.1073/pnas.0709632105 18287047 PMC2268599

[B138] Mourrain P. Béclin C. Elmayan T. Feuerbach F. Godon C. Morel J. B. (2000). Arabidopsis SGS2 and SGS3 genes are required for posttranscriptional gene silencing and natural virus resistance. *Cell* 101 533–542. 10.1016/s0092-8674(00)80863-6 10850495

[B139] Muangsan N. Beclin C. Vaucheret H. Robertson D. (2004). Geminivirus VIGS of endogenous genes requires SGS2/SDE1 and SGS3 and defines a new branch in the genetic pathway for silencing in plants. *Plant J.* 38 1004–1014. 10.1111/j.1365-313X.2004.02103.x 15165191

[B140] Mukherjee K. Campos H. Kolaczkowski B. (2012). Evolution of animal and plant dicers: early parallel duplications and recurrent adaptation of antiviral RNA binding in plants. *Mol. Biol. Evol.* 30 627–641. 10.1093/molbev/mss263 23180579 PMC3563972

[B141] Nishikura K. (2001). A short primer on RNA silencing. *Cell* 107 415–418. 10.1016/s0092-8674(01)00581-5 11719182

[B142] Nizampatnam N. R. Kumar V. D. (2011). Intron hairpin and transitive RNA silencing mediated silencing of orfH522 transcripts restores male fertility in transgenic male sterile tobacco plants expressing orfH522. *Plant Mol. Biol.* 76 557–573. 10.1007/s11103-011-9789-6 21584859

[B143] Olmedo-Monfil V. Durán-Figueroa N. Arteaga-Vázquez M. Demesa-Arévalo E. Autran D. Grimanelli D. (2010). Control of female gamete formation by a small RNA pathway in *Arabidopsis*. *Nature* 464 628–632. 10.1038/nature08828 20208518 PMC4613780

[B144] Palauqui J. C. Elmayan T. Borne F. D. D. Crete P. Charles C. Vaucheret H. (1996). Frequencies, timing, and spatial patterns of co-suppression of nitrate reductase and nitrite reductase in transgenic tobacco plants. *Plant Physiol.* 112 1447–1456. 10.1104/pp.112.4.1447 12226457 PMC158076

[B145] Palauqui J.-C. Elmayan T. Pollien J. M. Vaucheret H. (1997). Systemic acquired silencing: transgene-specific post-transcriptional silencing is transmitted by grafting from silenced stocks to non-silenced scions. *Embo J.* 16 4738–4745. 10.1093/emboj/16.15.4738 9303318 PMC1170100

[B146] Palauqui J. C. Vaucheret H. (1998). Transgenes are dispensable for the RNA degradation step of cosuppression. *Proc. Natl. Acad. Sci. U.S.A.* 95 9675–9680. 10.1073/pnas.95.16.9675 9689140 PMC21398

[B147] Pandey P. Choudhury N. R. Mukherjee S. K. (2009). A geminiviral amplicon (VA) derived from Tomato leaf curl virus (ToLCV) can replicate in a wide variety of plant species and also acts as a VIGS vector. *Virol. J.* 6:152. 10.1186/1743-422X-6-152 19788728 PMC2761890

[B148] Pant B. D. Buhtz A. Kehr J. Scheible W.-R. (2008). MicroRNA399 is a long-distance signal for the regulation of plant phosphate homeostasis. *Plant J.* 53 731–738. 10.1111/j.1365-313x.2007.03363.x 17988220 PMC2268993

[B149] Parent J.-S. Bouteiller N. Elmayan T. Vaucheret H. (2014). Respective contributions of *Arabidopsis* DCL2 and DCL4 to RNA silencing. *Plant J.* 81 223–232. 10.1111/tpj.12720 25376953

[B150] Pasumarthy K. K. Mukherjee S. K. Choudhury N. R. (2011). The presence of tomato leaf curl Kerala virus AC3 protein enhances viral DNA replication and modulates virus induced gene-silencing mechanism in tomato plants. *Virol. J.* 8:178. 10.1186/1743-422X-8-178 21496351 PMC3102638

[B151] Pennazio S. Roggero P. Conti M. (1999). Recovery of plants from viral diseases: historical and new perspectives / Erholung der Pflanzen von Viruskrankheiten: Geschichtliche und neue Perspektiven. *J. Plant Dis. Protect.* 106 128–139.

[B152] Peragine A. Yoshikawa M. Wu G. Albrecht H. L. Poethig R. S. (2004). SGS3 and SGS2/SDE1/RDR6 are required for juvenile development and the production of trans-acting siRNAs in *Arabidopsis*. *Genes Dev.* 18 2368–2379. 10.1101/gad.1231804 15466488 PMC522987

[B153] Petersen B. O. Albrechtsen M. (2005). Evidence implying only unprimed RdRP activity during transitive gene silencing in plants. *Plant Mol. Biol.* 58 575–583. 10.1007/s11103-005-7307-4 16021340

[B154] Petsch K. A. Ma C. Scanlon M. J. Jorgensen R. A. (2010). Targeted forward mutagenesis by transitive RNA silencing. *Plant J.* 61 873–882. 10.1111/j.1365-313X.2009.04104.x 20003132

[B155] Prigigallo M. I. Križnik M. Paola D. Catalano D. Gruden K. Finetti-Sialer M. M. (2019). Potato Virus Y infection alters small RNA metabolism and immune response in tomato. *Viruses* 11:1100. 10.3390/v11121100 31783643 PMC6950276

[B156] Qi Y. He X. Wang X.-J. Kohany O. Jurka J. Hannon G. J. (2006). Distinct catalytic and non-catalytic roles of ARGONAUTE4 in RNA-directed DNA methylation. *Nature* 443 1008–1012. 10.1038/nature05198 16998468

[B157] Qin C. Li B. Fan Y. Zhang X. Yu Z. Ryabov E. (2017). Roles of Dicer-Like Proteins 2 and 4 in intra- and intercellular antiviral silencing. *Plant Physiol.* 174 1067–1081. 10.1104/pp.17.00475 28455401 PMC5462052

[B158] Qu F. Ye X. Morris T. J. (2008). Arabidopsis DRB4, AGO1, AGO7, and RDR6 participate in a DCL4-initiated antiviral RNA silencing pathway negatively regulated by DCL1. *Proc. Natl. Acad. Sci. U.S.A.* 105 14732–14737. 10.1073/pnas.0805760105 18799732 PMC2567185

[B159] Raja P. Wolf J. N. Bisaro D. M. (2010). RNA silencing directed against geminiviruses: post-transcriptional and epigenetic components. *Biochim. Biophys. Acta* 1799 337–351. 10.1016/j.bbagrm.2010.01.004 20079472

[B160] Ramirez-Prado J. S. Piquerez S. Bendahmane A. Hirt H. Raynaud C. Benhamed M. (2018). Modify the histone to win the battle: chromatin dynamics in plant-pathogen interactions. *Front. Plant Sci.* 9:355. 10.3389/fpls.2018.00355 29616066 PMC5868138

[B161] Ratcliff F. G. MacFarlane S. A. Baulcombe D. C. (1999). Gene silencing without DNA- RNA-mediated cross-protection between viruses. *Plant Cell* 11 1207–1216. 10.1105/tpc.11.7.1207 10402423 PMC144281

[B162] Rodriguez-Medina C. Atkins C. A. Mann A. J. Jordan M. E. Smith P. M. (2011). Macromolecular composition of phloem exudate from white lupin (*Lupinus albus* L.). *BMC Plant Biol.* 11:36. 10.1186/1471-2229-11-36 21342527 PMC3055823

[B163] Rodríguez-Negrete E. A. Carrillo-Tripp J. Rivera-Bustamante R. F. (2009). RNA silencing against geminivirus: complementary action of posttranscriptional gene silencing and transcriptional gene silencing in host recovery. *J. Virol.* 83 1332–1340. 10.1128/JVI.01474-08 19019951 PMC2620903

[B164] Rosa C. Kuo Y. W. Wuriyanghan H. Falk B. W. (2018). RNA interference mechanisms and applications in plant pathology. *Annu. Rev. Phytopathol.* 56 581–610. 10.1146/annurev-phyto-080417-050044 29979927

[B165] Rosas-Diaz T. Zhang D. Fan P. Wang L. Ding X. Jiang Y. (2018). A virus-targeted plant receptor-like kinase promotes cell-to-cell spread of RNA silencing. *Proc. Natl. Acad. Sci. U.S.A.* 115 1388–1393. 10.1073/pnas.1715556115 29363594 PMC5819414

[B166] Sammons R. Ivashuta S. Liu H. Wang D. Feng P. Kouranov A. (2011). *Polynucleotide Molecules for Gene Regulation in Plants. U.S. Patent 2011/0296556 A1.*

[B167] Sanan-Mishra N. Chakraborty S. Gupta D. Mukherjee S. K. (2017). “RNA silencing suppressors: biology and mechanisms,” in *Plant Epigenetics RNA Technologies*, eds Rajewsky N. Jurga S. Barciszewski J. (Cham: Springer International Publishing), 199–230.

[B168] Santovito E. Mascia T. Siddiqui S. A. Minutillo S. A. Valkonen J. P. Gallitelli D. (2014). Infection cycle of Artichoke Italian latent virus in tobacco plants: meristem invasion and recovery from disease symptoms. *PLoS One* 9:e99446. 10.1371/journal.Pone.0099446 24911029 PMC4050035

[B169] Sarkies P. Miska E. A. (2014). Small RNAs break out: the molecular cell biology of mobile small RNAs. *Nat. Rev. Mol. Cell Biol.* 15 525–535. 10.1038/nrm3840 25053358

[B170] Sastry K. S. Mandal B. Hammond J. Scott S. W. Briddon R. W. (2019). *Encyclopedia of Plant Viruses and Viroids.* Berlin: Springer.

[B171] Saunders K. Bedford I. D. Stanley J. (2002). Adaptation from whitefly to leafhopper transmission of an autonomously replicating nanovirus-like DNA component associated with ageratum yellow vein disease. *J. Gen. Virol.* 83(Pt 4), 907–913. 10.1099/0022-1317-83-4-907 11907341

[B172] Saxena S. Jónsson Z. Dutta A. (2003). Small RNAs with imperfect match to endogenous mRNA repress translation. *J. Biol. Chem.* 278 44312–44319. 12952966 10.1074/jbc.M307089200

[B173] Schiebel W. Haas B. Marinkoviæ S. Klanner A. Sänger H. L. (1993a). RNA-directed RNA polymerase from tomato leaves.I. Purification and physical properties. *J. Biol. Chem.* 268 11851–11857. 7685022

[B174] Schiebel W. Haas B. Marinkoviæ S. Klanner A. Sänger H. L. (1993b). RNA-directed RNA polymerase from tomato leaves.II.Catalytic in vitro properties. *J. Biol. Chem.* 268 11858–11867. 7685023

[B175] Schiebel W. Pélissier T. Riedel L. Thalmeir S. Schiebel R. Kempe D. (1998). Isolation of an RNA-Directed RNA Polymerase-specific cDNA clone from tomato. *Plant Cell* 10 2087–2101. 10.1105/tpc.10.12.2087 9836747 PMC143969

[B176] Schwab R. Maizel A. Ruiz-Ferrer V. Garcia D. Bayer M. Crespi M. (2009). Endogenous TasiRNAs mediate non-cell autonomous effects on gene regulation in *Arabidopsis thaliana*. *PLoS One* 4:e5980. 10.1371/journal.Pone.0005980 19543387 PMC2694355

[B177] Schwach F. Vaistij F. E. Jones L. Baulcombe D. C. (2005). An RNA-dependent RNA polymerase prevents meristem invasion by potato virus X and is required for the activity but not the production of a systemic silencing signal. *Plant Physiol.* 138 1842–1852. 10.1104/pp.105.063537 16040651 PMC1183376

[B178] Searle I. R. Pontes O. Melnyk C. W. Smith L. M. Baulcombe D. C. (2010). JMJ14, a JmjC domain protein, is required for RNA silencing and cell-to-cell movement of an RNA silencing signal in *Arabidopsis*. *Genes Dev.* 24 986–991. 10.1101/gad.579910 20478993 PMC2867213

[B179] Senshu H. Yamaji Y. Minato N. Shiraishi T. Maejima K. Hashimoto M. (2011). A dual strategy for the suppression of host antiviral silencing: two distinct suppressors for viral replication and viral movement encoded by potato virus M. *J. Virol.* 85 10269–10278. 10.1128/JVI.05273-11 21752911 PMC3196401

[B180] Shahid S. Kim G. Johnson N. R. Wafula E. Wang F. Coruh C. (2018). MicroRNAs from the parasitic plant *Cuscuta campestris* target host messenger RNAs. *Nature* 553 82–85. 10.1038/nature25027 29300014

[B181] Shao F. Lu S. (2014). Identification, Molecular cloning and expression analysis of five RNA-dependent RNA polymerase genes in *Salvia miltiorrhiza*. *PLoS One* 9:e95117. 10.1371/journal.Pone.0095117 24733018 PMC3986363

[B182] Shi M. Z. Xie D. Y. (2014). Biosynthesis and metabolic engineering of anthocyanins in *Arabidopsis thaliana*. *Recent Patents Biotechnol.* 8 47–60. 10.2174/1872208307666131218123538 24354533 PMC4036305

[B183] Si-Ammour A. Windels D. Arn-Bouldoires E. Kutter C. Ailhas J. Meins F. (2011). miR393 and secondary siRNAs regulate expression of the TIR1/AFB2 auxin receptor clade and auxin-related development of *Arabidopsis* leaves. *Plant Physiol.* 157 683–691. 10.1104/pp.111.180083 21828251 PMC3192580

[B184] Sijen T. Fleenor J. Simmer F. Thijssen K. L. Parrish S. Timmons L. (2001). On the role of RNA amplification in dsRNA-triggered gene silencing. *Cell* 107 465–476. 10.1016/s0092-8674(01)00576-1 11719187

[B185] Singh A. Mohorianu I. Green D. Dalmay T. Dasgupta I. Mukherjee S. K. (2019). Artificially induced phased siRNAs promote virus resistance in transgenic plants. *Virology* 537 208–215. 10.1016/j.virol.2019.08.032 31513956

[B186] Singh A. Taneja J. Dasgupta I. Mukherjee S. K. (2015). Development of plants resistant to tomato geminiviruses using artificial trans-acting small interfering RNA. *Mol. Plant Pathol.* 16 724–734. 10.1111/mpp.12229 25512230 PMC6638473

[B187] Singh N. Mukherjee S. K. Rajam M. V. (2020). Silencing of the ornithine decarboxylase gene of *Fusarium oxysporum* f.sp.lycopersici by host-induced RNA silencing confers resistance to *Fusarium* wilt in tomato. *Plant Mol. Biol. Rep.* 38 419–429. 10.1007/s11105-020-01205-2

[B188] Skopelitis D. S. Benkovics A. H. Husbands A. Y. Timmermans M. C. (2017). Boundary formation through a direct threshold-based readout of mobile small RNA gradients. *Dev. Cell* 43 265.e–273.e. 10.1016/j.devcel.2017.10.003 29107557

[B189] Slotkin R. K. Vaughn M. Borges F. Tanurdžiæ M. Becker J. D. Feijó J. A. (2009). Epigenetic reprogramming and small RNA silencing of transposable elements in pollen. *Cell* 136 461–472. 10.1016/j.cell.2008.12.038 19203581 PMC2661848

[B190] Smardon A. Spoerke J. M. Stacey S. C. Klein M. E. Mackin N. Maine E. M. (2000). EGO-1 is related to RNA-directed RNA polymerase and functions in germ-line development and RNA interference in *C. elegans*. *Curr. Biol.* 10 169–178. 10.1016/s0960-9822(00)00323-7 10704412

[B191] Smith L. M. Pontes O. Searle I. Yelina N. Yousafzai F. K. Herr A. J. (2007). An SNF2 protein associated with nuclear RNA silencing and the spread of a silencing signal between cells in arabidopsis. *Plant Cell* 19 1507–1521. 10.1105/tpc.107.051540 17526749 PMC1913737

[B192] Smith N. A. Eamens A. L. Wang M. B. (2011). Viral small interfering RNAs target host genes to mediate disease symptoms in plants. *PLoS Pathog.* 7:e1002022. 10.1371/journal.ppat.1002022 21573142 PMC3088724

[B193] Smith N. A. Singh S. P. Wang M. B. Stoutjesdijk P. A. Green A. G. Waterhouse P. M. (2000). Total silencing by intron-spliced hairpin RNAs. *Nature* 407 319–320. 10.1038/35030305 11014180

[B194] Song C. Yu M. Han J. Wang C. Liu H. Zhang Y. (2012a). Validation and charactrisation of *Citrus sinesis* microRNAs and their target genes. *BMC Res. Notes* 5:235. 10.1186/1756-0500-5-235 22583737 PMC3436860

[B195] Song X. Li P. Zhai J. Zhou M. Ma L. Liu B. (2012b). Roles of DCL4 and DCL3b in rice phased small RNA biogenesis. *Plant J.* 69 462–474. 10.1111/j.1365-313X.2011.04805.x 21973320

[B196] Song X. Wang D. Ma L. Chen Z. Li P. Cui X. (2012c). Rice RNA-dependent RNA polymerase 6 acts in small RNA biogenesis and spikelet development. *Plant J.* 71 378–389. 10.1111/j.1365-313X.2012.05001.x 22443269

[B197] Sosa-Valencia G. Palomar M. Covarrubias A. A. Reyes J. L. (2017). The legume miR1514a modulates a NAC transcription factor transcript to trigger phasiRNA formation in response to drought. *J. Exp. Bot.* 68 2013–2026. 10.1093/jxb/erw380 28338719 PMC5429018

[B198] Srikant T. Wibowo A. Schwab R. Weigel D. (2019). Position-dependent effects of cytosine methylation on FWA expression in *Arabidopsis thaliana*. *BioRxiv* 10.1101/774281

[B199] Tang G. Reinhart B. J. Bartel D. P. Zamore P. D. (2003). A biochemical framework for RNA silencing in plants. *Genes Dev.* 17 49–63. 10.1101/gad.1048103 12514099 PMC195971

[B200] Taochy C. Gursanscky N. R. Cao J. Fletcher S. J. Dressel U. Mitter N. (2017). A genetic screen for impaired systemic RNA silencing highlights the crucial role of DICER-LIKE 2. *Plant Physiol.* 175 1424–1437. 10.1104/pp.17.01181 28928141 PMC5664484

[B201] Tijsterman M. Ketting R. F. Okihara K. L. Sijen T. Plasterk R. H. (2002). RNA helicase MUT-14-dependent gene silencing triggered in *C. elegans* by short antisense RNAs. *Science* 295 694–697. 10.1126/science.1067534 11809977

[B202] Vaistij F. E. Jones L. (2009). Compromised virus-induced gene silencing in RDR6-deficient plants. *Plant Physiol.* 149 1399–1407. 10.1104/pp.108.132688 19129420 PMC2649407

[B203] Vaistij F. E. Jones L. Baulcombe D. C. (2002). Spreading of RNA targeting and DNA methylation in RNA silencing requires transcription of the target gene and a putative RNA-dependent RNA polymerase. *Plant Cell* 14 857–867. 10.1105/tpc.010480 11971140 PMC150687

[B204] Vargas-Asencio J. A. Perry K. L. (2020). A small RNA-mediated regulatory network in *Arabidopsis thaliana* demonstrates connectivity between phasiRNA regulatory modules and extensive co-regulation of transcription by miRNAs and phasiRNAs. *Front. Plant Sci.* 10:1710. 10.3389/fpls.2019.01710 32082334 PMC7001039

[B205] Vazquez F. Hohn T. (2013). Biogenesis and biological activity of secondary siRNAs in plants. *Scientifica* 2013:783253. 10.1155/2013/783253 24278785 PMC3820352

[B206] Vazquez F. Vaucheret H. Rajagopalan R. Lepers C. Gasciolli V. Mallory A. C. (2004). Endogenous trans-Acting siRNAs regulate the accumulation of *Arabidopsis* mRNAs. *Mol. Cell* 16 69–79. 10.1016/j.molcel.2004.09.028 15469823

[B207] Verlaan M. G. Hutton S. F. Ibrahem R. M. Kormelink R. Visser R. G. Scott J. W. (2013). The Tomato yellow leaf curl virus resistance genes Ty-1 and Ty-3 are allelic and code for DFDGD-class RNA-dependent RNA polymerases. *PLoS Genet.* 9:e1003399. 10.1371/journal.Pgen.1003399 23555305 PMC3610679

[B208] Vermeersch L. De Winne N. Nolf J. Bleys A. Kovaøík A. Depicker A. (2013). Transitive RNA silencing signals induce cytosine methylation of a transgenic but not an endogenous target. *Plant J.* 74 867–879. 10.1111/tpj.12172 23480471

[B209] Vogler H. Kwon M. O. Dang V. Sambade A. Fasler M. Ashby J. (2008). Tobacco mosaic virus movement protein enhances the spread of RNA silencing. *PLoS Pathog.* 4:e1000038. 10.1371/journal Ppat.1000038 PMC2270343 18389061

[B210] Voinnet O. (2005). Non-cell autonomous RNA silencing. *FEBS Lett.* 579 5858–5871. 10.1016/j.febslet.2005.09.039 16242131

[B211] Voinnet O. Baulcombe D. C. (1997). Systemic signalling in gene silencing. *Nature* 389 553–553. 10.1038/39215 9335491

[B212] Voinnet O. Lederer C. Baulcombe D. C. (2000). A Viral Movement protein prevents spread of the gene silencing signal in *Nicotiana benthamiana*. *Cell* 103 157–167. 10.1016/s0092-8674(00)00095-7 11051555

[B213] Voinnet O. Vain P. Angell S. Baulcombe D. C. (1998). Systemic spread of sequence-specific transgene RNA degradation in plants is initiated by localized introduction of ectopic promoterless DNA. *Cell* 95 177–187. 10.1016/s0092-8674(00)81749-3 9790525

[B214] Volpe T. A. Kidner C. Hall I. M. Teng G. Grewal S. I. Martienssen R. A. (2002). Regulation of heterochromatic silencing and histone H3 lysine-9 methylation by RNA silencing. *Science* 297 1833–1837. 10.1126/science.1074973 12193640

[B215] Voorburg C. M. Yan Z. Bergua-Videl M. Wolters A. A. Bai Y. Kormelink R. (2020). *Ty-1*, a universal resistance gene against geminiviruses that is compromised by co-replication of a betasatellite. *Mol. Plant Pathol.* 21 160–172. 31756021 10.1111/mpp.12885 PMC6988424

[B216] Vu T. V. Choudhury N. R. Mukherjee S. K. (2013). Transgenic tomato plants expressing artificial microRNAs for silencing the precoat and coat proteins of a begomovirus, Tomato leaf curl New Delhi virus, show tolerance to virus infection. *Virus Res.* 172 35–45. 10.1016/j.virusres.2012.12.008 23276684

[B217] Walsh H. A. Vanderschuren H. Taylor S. Rey M. (2019). RNA silencing of South African cassava mosaic virus in transgenic cassava expressing AC1/AC4 hp-RNA induces tolerance. *Biotechnol. Rep.* 24:e00383. 10.1016/j.btre.2019.e00383 31763196 PMC6864324

[B218] Wang H. Jiao X. Kong X. Hamera S. Wu Y. Chen X. (2016). A signaling cascade from miR444 to RDR1 in rice antiviral rna silencing pathway. *Plant Physiol.* 170 2365–2377. 10.1104/pp.15.01283 26858364 PMC4825140

[B219] Wang T. Deng Z. Zhang X. Wang H. Wang Y. Liu X. (2018). Tomato *DCL2b* is required for the biosynthesis of 22-nt small RNAs, the resulting secondary siRNAs, and the host defense against ToMV. *Hortic. Res.* 5:62. 10.1038/s41438-018-0073-7 30181890 PMC6119189

[B220] Wang K. Su X. Cui X. Du Y. Zhang S. Gao J. (2018). Identification and characterization of microRNA during *Bemisia tabaci* infestations in *Solanum lycopersicum* and *Solanum habrochaites*. *Hortic. Plant J.* 4 62–72. 10.1016/j.hpj.2018.03.002

[B221] Wang X. B. Jovel J. Udomporn P. Wang Y. Wu Q. Li W. X. (2011). The 21-nucleotide, but Not 22-nucleotide, viral secondary small interfering RNAs direct potent antiviral defense by two cooperative argonautes in *Arabidopsis thaliana*. *Plant Cell* 23 1625–1638. 10.1105/tpc.110.082305 21467580 PMC3101545

[B222] Wang X. B. Wu Q. Ito T. Cillo F. Li W. X. Chen X. (2010). RNA silencing-mediated viral immunity requires amplification of virus-derived siRNAs in *Arabidopsis thaliana*. *Proc. Natl. Acad. Sci. U.S.A.* 107 484–489. 10.1073/pnas.0904086107 19966292 PMC2806737

[B223] Wang Y. Wu Y. Gong Q. Ismayil A. Yuan Y. Lian B. (2019). Geminiviral V2 protein suppresses transcriptional gene silencing through interaction with AGO4. *J. Virol.* 93 e1675–e1618. 10.1128/JVI.01675-18 PMC6401443 30626668

[B224] Wassenegger M. Krczal G. (2006). Nomenclature and functions of RNA-directed RNA polymerases. *Trends Plant Sci.* 11 142–151. 10.1016/j.tplants.2006.01.003 16473542

[B225] Waterhouse P. M. Graham M. W. Wang M. B. (1998). Virus resistance and gene silencing in plants can be induced by simultaneous expression of sense and antisense RNA. *Proc. Natl. Acad. Sci. U.S.A.* 95 13959–13964. 10.1073/pnas.95.23.13959 9811908 PMC24986

[B226] Weiberg A. Wang M. Lin F. M. Zhao H. Zhang Z. Kaloshian I. (2013). Fungal small RNAs suppress plant immunity by hijacking host RNA interference pathways. *Science* 342 118–123. 24092744 10.1126/science.1239705 PMC4096153

[B227] Wen M. Lin X. Xie M. Wang Y. Shen X. Liufu Z. (2016). Small RNA transcriptomes of mangroves evolve adaptively in extreme environments. *Sci. Rep.* 6:27551. 10.1038/srep27551 27278626 PMC4899726

[B228] Wierzbicki A. T. Ream T. S. Haag J. R. Pikaard C. S. (2009). RNA polymerase V transcription guides ARGONAUTE4 to chromatin. *Nat. Genet.* 41 630–634. 10.1038/ng.365 19377477 PMC2674513

[B229] Willmann M. R. Endres M. W. Cook R. T. Gregory B. D. (2011). The functions of RNA-dependent RNA polymerases in *Arabidopsis*. *Arabid. Book* 9:e0146. 10.1199/tab.0146 PMC3268507 22303271

[B230] Windels D. Vazquez F. (2011). miR393: integrator of environmental cues in auxin signaling? *Plant Signal. Behav.* 6 1672–1675. 10.4161/psb.6.11.17900 22067993 PMC3329333

[B231] Wingard S. A. (1928). Hosts and symptoms of ring spot a virus disease of plants. *J. Agric. Res.* 37 127–153.

[B232] Won S. Y. Yumul R. E. Chen X. (2014). “Small RNAs in plants,” in *Molecular Biology. The Plant Sciences*, Vol. 2 ed. Howell S. (New York, NY: Springer), 95–127.

[B233] Wu H. Qu X. Dong Z. Luo L. Shao C. Forner J. (2020). WUSCHEL triggers innate antiviral immunity in plant stem cells. *Science* 370 227–231. 33033220 10.1126/science.abb7360

[B234] Wu L. Mao L. Qi Y. (2012). Roles of dicer-like and argonaute proteins in TAS-derived small interfering RNA-triggered DNA methylation. *Plant Physiol.* 160 990–999. 10.1104/pp.112.200279 22846193 PMC3461571

[B235] Xia J. Wang X. Perroud P. F. He Y. Quatrano R. Zhang W. (2016). Endogenous small-noncoding RNAs and potential functions in desiccation tolerance in *Physcomitrella patens*. *Sci. Rep.* 6:30118. 10.1038/srep30118 PMC4957126 27443635

[B236] Xia R. Xu J. Meyers B. C. (2017). The emergence, evolution, and diversification of the miR390-TAS3-ARF pathway in land plants. *Plant Cell* 29 1232–1247. 10.1105/tpc.17.00185 28442597 PMC5502456

[B237] Xia R. Ye S. Liu Z. Meyers B. C. Liu Z. (2015). Novel and recently evolved microRNA clusters regulate expansive F-Box gene networks through phased small interfering RNAs in wild diploid strawberry. *Plant Physiol.* 169 594–610. 10.1104/pp.15.00253 26143249 PMC4577376

[B238] Xia Z. Zhao Z. Jiao Z. Xu T. Wu Y. Zhou T. (2018). Virus-derived small interfering RNAs affect the accumulations of viral and host transcripts in maize. *Viruses* 10:664. 10.3390/v10120664 30477197 PMC6315483

[B239] Xie Z. Allen E. Wilken A. Carrington J. C. (2005). DICER-LIKE 4 functions in trans-acting small interfering RNA biogenesis and vegetative phase change in *Arabidopsis thaliana*. *Proc. Natl. Acad. Sci. U.S.A.* 102 12984–12989. 10.1073/pnas.0506426102 16129836 PMC1200315

[B240] Xie Z. Johansen L. K. Gustafson A. M. Kasschau K. D. Lellis A. D. Zilberman D. (2004). Genetic and functional diversification of small RNA pathways in plants. *PLoS Biol.* 2:E104. 10.1371/journal.Pbio.0020104 15024409 PMC350667

[B241] Yang X. Xie Y. Raja P. Li S. Wolf J. N. Shen Q. (2011). Suppression of methylation-mediated transcriptional gene silencing by βC1-SAHH protein interaction during geminivirus-betasatellite infection. *PLoS Pathog.* 7:e1002329. 10.1371/journal.ppat.1002329 22028660 PMC3197609

[B242] Yoo B.-C. Kragler F. Varkonyi-Gasic E. Haywood V. Archer-Evans S. Lee Y. M. (2004). A systemic small RNA signaling system in plants. *Plant Cell* 16 1979–2000. 10.1105/tpc.104.023614 15258266 PMC519190

[B243] Yoshikawa M. Iki T. Numa H. Miyashita K. Meshi T. Ishikawa M. (2016). A short open reading frame encompassing the microRNA173 target site plays a role in trans-acting small interfering RNA biogenesis. *Plant Physiol.* 171 359–368. 10.1104/pp.16.00148 26966170 PMC4854708

[B244] Yoshikawa M. Peragine A. Park M. Y. Poethig R. S. (2005). A pathway for the biogenesis of trans-acting siRNAs in *Arabidopsis*. *Genes Dev.* 19 2164–2175. 10.1101/gad.1352605 16131612 PMC1221887

[B245] Yu D. Fan B. MacFarlane S. A. Chen Z. (2003). Analysis of the involvement of an inducible *Arabidopsis* RNA-dependent RNA polymerase in antiviral defense. *Mol. Plant Microbe Interact.* 16 206–216. 10.1094/MPMI.2003.16.3.206 12650452

[B246] Zachert S. Schuck J. Weinholdt C. Knoblich M. Pantaleo V. Grosse I. (2019). Highly efficacious antiviral protection of plants by small interfering RNAs identified *in vitro*. *Nucleic Acids Res.* 47 9343–9357. 31433052 10.1093/nar/gkz678 PMC6755098

[B247] Zhai J. Jeong D.-H. Paoli E. D. Park S. Rosen B. D. Li Y. (2011). MicroRNAsas master regulators of the plant NB-LRR gene family via the production of phased, trans-acting siRNAs. *Genes Dev.* 25 2540–2553. 10.1101/gad.177527.111 22156213 PMC3243063

[B248] Zhai J. Zhang H. Arikit S. Huang K. Nan G. L. Walbot V. (2015). Spatiotemporally dynamic, cell-type-dependent premeiotic and meiotic phasiRNAs in maize anthers. *Proc. Natl. Acad. Sci. U.S.A.* 112 3146–3151. 10.1073/pnas.1418918112 25713378 PMC4364226

[B249] Zhang R. Huang S. Li S. Song G. Li Y. Li W. (2020). Evolution of PHAS loci in the young spike of allohexaploid wheat. *BMC Genomics* 21:200. 10.1186/s12864-020-6582-4 PMC7057497 32131726

[B250] Zhang W. Kollwig G. Stecyk E. Apelt F. Dirks R. Kragler F. (2014). Graft-transmissible movement of inverted-repeat-induced siRNA signals into flowers. *Plant J.* 80 106–121. 10.1111/tpj.12622 25039964

[B251] Zhang X. Lai T. Zhang P. Zhang X. Yuan C. Jin Z. (2019). Mini review: revisiting mobile RNA silencing in plants. *Plant Sci.* 278 113–117. 10.1016/j.Plantsci.2018.10.025 30471724 PMC6556431

[B252] Zhang X. Lii Y. Wu Z. Polishko A. Zhang H. Chinnusamy V. (2013). Mechanisms of small RNA generation from cis-NATS in response to environmental and developmental cues. *Mol. Plant* 6 704–715. 10.1093/mp/sst051 23505223 PMC3660955

[B253] Zhang X. Xia J. Lii Y. E. Barrera-Figueroa B. E. Zhou X. Gao S. (2012). Genome-wide analysis of plant nat-siRNAs reveals insights into their distribution, biogenesis and function. *Genome Biol.* 13:R20. 10.1186/gb-2012-13-3-r20 22439910 PMC3439971

[B254] Zhang X. P. Liu D. S. Yan T. Fang X. D. Dong K. Xu J. (2017). Cucumber mosaic virus coat protein modulates the accumulation of 2b protein and antiviral silencing that causes symptom recovery *in planta*. *PLoS Pathog.* 13:e1006522. 10.1371/journal.Ppat.1006522 28727810 PMC5538744

[B255] Zhang Z. J. (2014). Artificial trans-acting small interfering RNA: a tool for plant biology study and crop improvements. *Planta* 239 1139–1146. 10.1007/s00425-014-2054-x 24643516

[B256] Zhao J. H. Fang Y. Y. Duan C. G. Fang R. X. Ding S. W. Guo H. S. (2016). Genome-wide identification of endogenous RNA-directed DNA methylation loci associated with abundant 21-nucleotide siRNAs in *Arabidopsis*. *Sci. Rep.* 6:36247. 10.1038/srep36247 27786269 PMC5081565

[B257] Zhao M. Cai C. Zhai J. Lin F. Li L. Shreve J. (2015a). Coordination of MicroRNAs, PhasiRNAs and NB-LRR genes in response to plant pathogen: insights from analyses of a set of Soybean *rps* gene near-isogenis lines. *Plant Genome* 8 1–13. 10.3835/plantgenome 2014.09.0044 33228285

[B258] Zhao M. San León D. Mesel F. García J. A. Simón-Mateo C. (2015b). Assorted processing of synthetic trans-acting sirnas and its activity in antiviral resistance. *PLoS One* 10:e0132281. 10.1371/journal.Pone.0132281 26147769 PMC4492489

[B259] Zheng X. Yang L. Li Q. Ji L. Tang A. Zang L. (2018). MIGS as a simple and efficient method for gene silencing in rice. *Front. Plant Sci.* 9:662. 10.3389/fpls.2018.00662 29868104 PMC5964998

[B260] Zheng X. Zhu J. Kapoor A. Zhu J.-K. (2007). Role of *Arabidopsis* AGO6 in siRNA accumulation, DNA methylation and transcriptional gene silencing. *EMBO J.* 26 1691–1701. 10.1038/sj.emboj.7601603 17332757 PMC1829372

[B261] Zheng Y. Wang Y. Wu J. Ding B. Fei Z. (2015). A dynamic evolutionary and functional landscape of plant phased small interfering RNAs. *BMC Biol.* 13:32. 10.1186/s12915-015-0142-4 25980406 PMC4457045

[B262] Zilberman D. Cao X. Jacobsen S. E. (2003). ARGONAUTE4 control of locus-specific siRNA accumulation and DNA and histone methylation. *Science* 299 716–719. 10.1126/science.1079695 12522258

